# ER-misfolded proteins become sequestered with mitochondria and impair mitochondrial function

**DOI:** 10.1038/s42003-021-02873-w

**Published:** 2021-12-02

**Authors:** Adrián Cortés Sanchón, Harshitha Santhosh Kumar, Matilde Mantovani, Ivan Osinnii, José María Mateos, Andres Kaech, Dimitri Shcherbakov, Rashid Akbergenov, Erik C. Böttger

**Affiliations:** 1 Institut für Medizinische Mikrobiologie, Universität Zürich, 8006 Zurich, Switzerland; 2 Center for Microscopy and Image Analysis, University of Zurich, 8057 Zurich, Switzerland

**Keywords:** ER-associated degradation, ER-associated degradation

## Abstract

Proteostasis is a challenge for cellular organisms, as all known protein synthesis machineries are error-prone. Here we show by cell fractionation and microscopy studies that misfolded proteins formed in the endoplasmic reticulum can become associated with and partly transported into mitochondria, resulting in impaired mitochondrial function. Blocking the endoplasmic reticulum-mitochondria encounter structure (ERMES), but not the mitochondrial sorting and assembly machinery (SAM) or the mitochondrial surveillance pathway components Msp1 and Vms1, abrogated mitochondrial sequestration of ER-misfolded proteins. We term this mitochondria-associated proteostatic mechanism for ER-misfolded proteins ERAMS (ER-associated mitochondrial sequestration). We testify to the relevance of this pathway by using mutant α-1-antitrypsin as an example of a human disease-related misfolded ER protein, and we hypothesize that ERAMS plays a role in pathological features such as mitochondrial dysfunction.

## Abstract (web summary)

Sanchón et al find that misfolded proteins formed in the ER can become associated with mitochondria, both in mammalian cells and in yeast, resulting in impaired mitochondrial function. They further discover that components of ERMES-mediated ER-mitochondria contacts are needed for this mechanism, which they name ERAMS, for ER-associated mitochondrial sequestration.

## Introduction

Protein homeostasis depends on a delicate balance between maintaining protein conformation, refolding misfolded proteins and degrading damaged proteins. This process is most finely controlled in the endoplasmic reticulum (ER). The ER is a main hub of protein production and serves as the synthesis site for secretory and membrane proteins. More than one-third of a cell’s proteome is typically synthesized in the ER^1^. Proteins enter the ER unfolded and are processed by a complex protein folding machinery consisting of foldases, oxidoreductases, chaperones, lectins and oligosaccharide processing enzymes to facilitate proper folding^2^. In general, proteins only leave the ER after they have reached their native states. However, protein folding in the ER is relatively slow and inefficient and requires the continuous removal of polypeptides that fail to attain their native structure^2,3^. To offset the harmful consequences of misfolded protein accumulation, ER-retained misfolded proteins are most commonly destroyed. The primary mechanism of disposal is referred to as ER-associated degradation (ERAD). During ERAD, terminally misfolded proteins are delivered to the proteasome, which resides in the cytoplasm. Destruction of ERAD substrates thus requires delivery from the ER by retrotranslocation into the cytoplasm, where they become subsequently processed and degraded by the ubiquitin-proteasome system^4,5^.

ER and mitochondria form physical contact sites, resulting in electron-dense structures that bridge these two organelles^6,7^. These membrane contact sites are also referred to as mitochondria-associated membranes (MAMs)^8^. More recently, a protein complex termed ERMES (ER-mitochondria encounter structure) has been identified that tethers ER and mitochondria and that facilitates the exchange of Ca^++^ and lipids between these two organelles^9^. ER-mitochondria tethers have been implicated in lipid homeostasis, the formation of autophagosomes, and apoptotic signalling^10–13^, contributing to the view that ER-mitochondria connections are hubs for integrating interorganelle crosstalk. Here, we addressed the question of whether further to these functions, mitochondria play a role in ER proteostasis.

## Results

### ER-Fluc is targeted to ER, processed, and misfolded

**Fig. 1:**
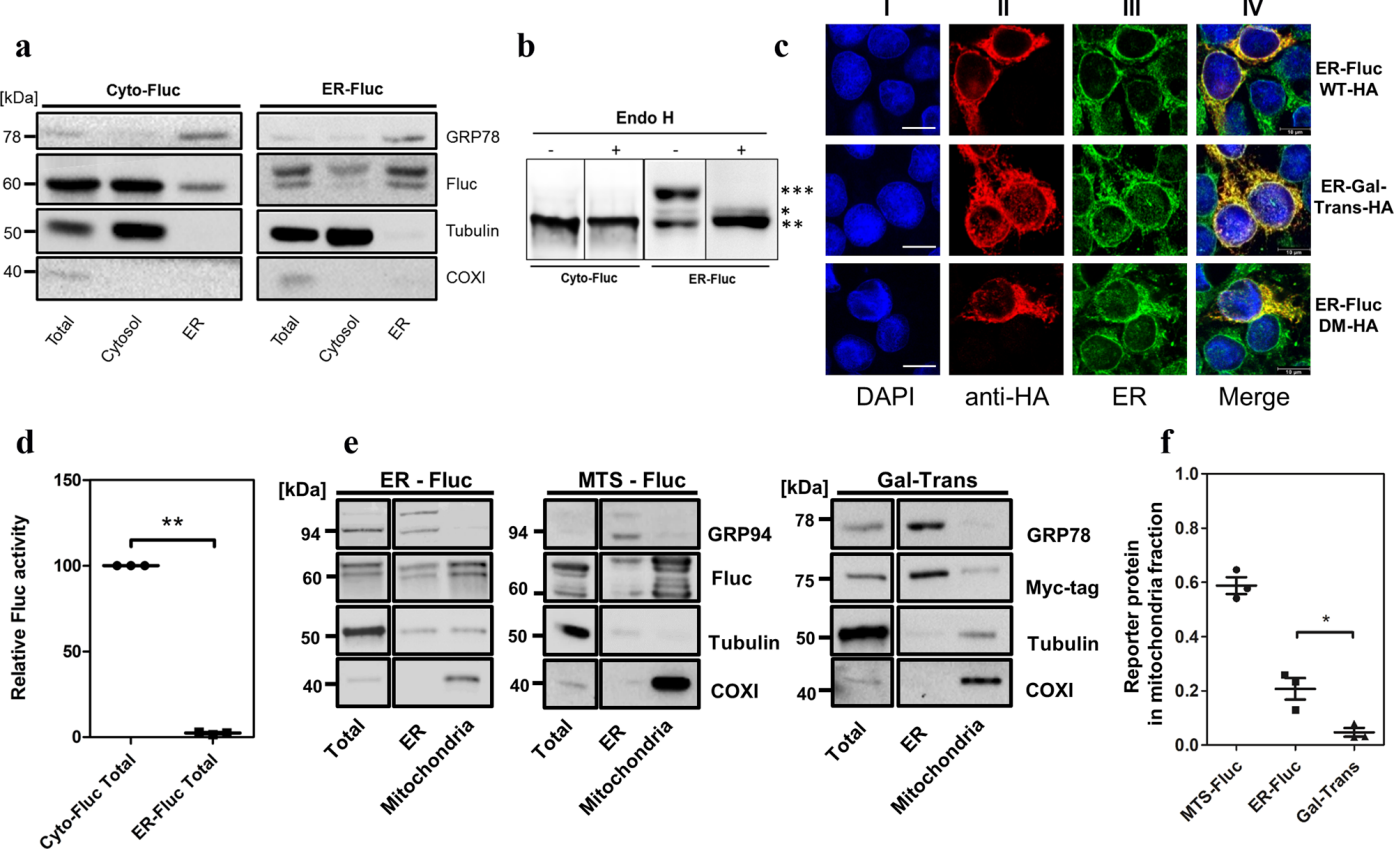
Subcellular localization of ER-Fluc. **a** Representative Western blot analysis of cellular fractions isolated from HEK293 cells expressing cyto-Fluc and ER-Fluc; marker proteins to follow fractionation were: GRP78 (ER), COX1 (mitochondria), tubulin (cytosol). **b** Representative Western blot of cyto-Fluc and ER-Fluc samples treated with endoglycosidase H; three different bands are identified for ER-Fluc: *** ER-localized cleaved, glycosylated Fluc (upper band), ** ER-localized cleaved, non-glycosylated Fluc (lower band), * not cleaved cytosolic-localized Fluc (middle band). As per construction of ER-Fluc, the cleaved and non-glycosylated ER-Fluc has the same molecular weight as cyto-Fluc. **c** Confocal microscopy to demonstrate ER localization of HA-tagged ER-Fluc WT and HA-tagged ER-FlucDM; transfection with HA-tagged resident ER protein galactosyltransferase (Gal-Trans) was used as positive control. **I—**DAPI, blue; **II—**anti-HA-tag, red; **III—**anticalreticulin (ER-marker), green; **IV—**overlay I), II) and III), co-localization is indicated by yellow colour. Scale bar = 10 µm. **d**) Firefly luciferase activities of cyto-Fluc and ER-Fluc; luciferase activity was normalized per quantification of Western blots, cyto-Fluc was set as 100 (*N* = 3), **p = 0.004. For details see Supplementary Fig. 1. **e** Representative Western blots of total cellular lysate, ER and mitochondrial fractions isolated from HEK293 cells expressing ER-Fluc, MTS-Fluc, and myc-tagged Gal-Trans. GRP94 or GRP78 were used as marker for ER. **f** Semiquantitative assessment of MTS-Fluc, ER-Fluc and Gal-Trans present in isolated mitochondrial fractions. (*N* = 3, error bars SD). *p < 0.05. N - number of independent experiments. Reporter proteins (MTS-Fluc, ER-Fluc, Gal-Trans) were normalized to COXI and their recovery in mitochondrial fraction relative to total calculated using the formula: $\frac{{{{{{\rm{reporter}}}}}}\,{{{{{\rm{protein}}}}}}\,{{{{{\rm{mitochondrial}}}}}}\,{{{{{\rm{fraction}}}}}}}{{{{{{\rm{COXI}}}}}}\,{{{{{\rm{mitochondrial}}}}}}\,{{{{{\rm{fraction}}}}}}}:\frac{{{{{{\rm{reporter}}}}}}\,{{{{{\rm{protein}}}}}}\,{{{{{\rm{total}}}}}}}{{{{{{\rm{COXI}}}}}}\,{{{{{\rm{total}}}}}}}$, (see Supplementary Fig. 3). See Supplementary Fig 16A–F for uncropped gel scans.

To assess and follow the trafficking of ER-localized misfolded proteins we used a modified misfolding-prone firefly luciferase reporter (Fluc)^14^. We targeted Fluc to the ER with the help of the prolactin signal peptide (PLN) placed upstream, and a KDEL ER-retention sequence placed downstream of the luciferase coding sequence (ER-Fluc construct). As a control, we used a similar construct without the PLN ER-targeting and the KDEL ER-retention signals, so that Fluc remains in the cytoplasm (cyto-Fluc construct). To assess the subcellular localization of the ER-Fluc constructs, HEK293 cells were transiently transfected with ER-Fluc and cyto-Fluc constructs and the cytosolic and ER fractions were isolated and analyzed by Western blot. Compared to cyto-Fluc which was found predominantly in the cytosolic fraction, ER-Fluc was significantly enriched in the ER fraction (Fig. 1a). Detection of Fluc by Western blot demonstrated two different molecular weight forms of ER-Fluc and we hypothesized that the higher molecular weight form reflects ER-mediated N-glycosylation. To corroborate the identity of the higher molecular weight form of ER-Fluc we used an endoglycosidase H (Endo H) treatment to deglycosylate the protein. Subsequent high-resolution SDS-gel analysis confirmed the identity of the higher molecular weight form as N-glycosylated Fluc and the lower molecular weight form as unglycosylated Fluc, both with cleavage of the signal peptide, demonstrating that ER-Fluc had passed into the ER (Fig. 1b). Apparently, while cleavage of the signal peptide is complete, glycosylation of the Fluc reporter in the ER is partial, most likely as a result of its non-ER nature. Further evidence for expression of the ER-Fluc reporter in the ER was provided by confocal microscopy, which revealed co-localization of ER-Fluc with the ER-marker protein calreticulin. Transfection with the HA-tagged ER-resident protein β-galactosyltransferase (Gal-Trans) served as a positive control (Fig. 1c).

As a possible indicator of protein misfolding we studied the activity of Fluc as enzymatic activity is reduced when Fluc is misfolded. The enzymatic activity of ER-Fluc was dramatically reduced compared to cyto-Fluc (Fig. 1d). This loss of activity cannot be explained by N-glycosylation of the ER-Fluc reporter or by the PLN-signal interfering with luciferase’s enzymatic activity, as a significant part of ER-Fluc is cleaved and not glycosylated (see Fig. 1b). We hypothesize that the inactivity of the ER-Fluc most likely reflects incorrect folding inside the endoplasmic reticulum.

### Mitochondrial localization of misfolded ER-Fluc

We studied the cellular localization of ER-Fluc using cell fractionation experiments. Surprisingly, Western blot analysis demonstrated that a substantial part of ER-Fluc is present in purified mitochondrial fractions (Fig. 1e). Transfections with mitochondria-targeted firefly luciferase (MTS-Fluc) and myc-tagged ER-resident protein Gal-Trans were used as positive and negative controls, respectively (Fig. 1e). Evidence for expression of mitochondria-targeted firefly luciferase (MTS-Fluc) in mitochondria was provided by immunofluorescence studies, which revealed co-localization with the mitochondrial marker protein TOM20 (Supplementary Fig. 2). Using the mitochondrial matrix protein COX1 for normalization, we estimated the relative amount of transfected reporters enriched in the mitochondrial fraction. Compared to the native ER-resident protein Gal-Trans, a significant amount of misfolded ER-Fluc was recovered in the mitochondrial fraction, for quantification see Fig. 1f and Supplementary Fig. 3.

The detection of the two major forms of ER-Fluc in the mitochondrial fraction, representing the ER-associated modifications of cleavage of the signal peptide and N-glycosylation, indicates that the ER-Fluc present in the mitochondrial fraction has passed through the ER. To further assess the association of misfolded ER-Fluc with mitochondria, we treated purified mitochondrial fractions with proteinase K to digest ER-Fluc attached to the outside of mitochondria. In addition, mitochondrial samples were subjected to proteinase K treatment following permeabilization of the outer mitochondrial membrane by hypotonic shock (20 mM HEPES). The ER-Fluc associated with the mitochondrial fraction was partially digested upon treatment with proteinase K. However, a significant part of the ER-Fluc stayed intact after treatment with proteinase K alone or following permeabilization of the outer membrane by hypotonic shock and was degraded only after treatment with proteinase K in combination with Triton X-100, dissolving both outer and inner mitochondrial membrane (Supplementary Fig. 4A, for quantification by densitometry analysis see Supplementary Fig. 4B). As control we used cells expressing mitochondria-targeted Fluc. MTS-Fluc in the mitochondrial fractions was protected from treatment with proteinase K alone or in combination with hypotonic shock and was degraded only after a combined treatment with proteinase K and Triton X-100 (Supplementary Fig. 4A, B). As controls for mitochondrial integrity and intramitochondrial localization we used proteins TOM70, TIM23, and COX1 for outer membrane, intermembrane space, and mitochondrial matrix, respectively.

### Microscopy studies in ER-Fluc expressing cells

The results of the cell fractionation experiments suggested that misfolded ER-Fluc is associated with mitochondria and becomes partially imported. However, cell fractionation experiments inherently are compromised by some pollution of the mitochondrial fraction with ER proteins. In addition, we cannot exclude that small amounts of protein aggregates accumulating in ER membrane inclusions may fragment away from the main ER network and potentially co-fractionate with mitochondria. To address these limitations, we decided to use a previously described split-GFP mito-localization system combined with confocal and electron microscopy and FACS analysis^15^. Split-GFP has the advantage of low off rates allowing for augmentation of even small signals. Briefly, this system consists of two vectors; one carrying the first ten β-strands of GFP fused to mitochondria-targeted mCherry (MTS-mCherry-GFP_1–10_) and one carrying the eleventh β-strand of GFP (GFP_11_) fused to a reporter protein. Manders’ correlation coefficient was used to demonstrate co-localization of the mitochondria-targeted mCherry-GFP_1–10_ with TOM20, a mitochondrial protein of the outer membrane (Supplementary Fig. 5). Mitochondria-localized GFP fluorescence is observed only when the GFP_11_-fused reporter associates with mitochondria and complements the mitochondrial-targeted mCherry-GFP_1–10_. In our experiments we used the ER-localized wild-type luciferase (ER-FlucWT-HA-GFP_11_), a double mutant aggregation-prone luciferase variant (FlucDM) targeted to ER (ER-FlucDM-HA-GFP_11_), the non-misfolding ER-resident protein Gal-Trans, and MTS-HA-GFP_11_ (as a positive control)^14^. Staining with antibodies against calreticulin was used to demonstrate ER localization of the ER-Fluc constructs and of ER-Gal-Trans (Fig. 1c). Alongside each reporter, cells were transiently co-transfected with MTS-mCherry-GFP_1–10_ and the resulting mitochondria-associated GFP fluorescence was measured by FACS and visualized by confocal microscopy. We further included co-transfection with MTS-Fluc-HA-GFP_11_ together with MTS-mCherry-GFP_1–10_ in this analysis to fully represent the reporters used in the cell fractionation experiments, i.e., ER-Fluc, MTS-Fluc, and ER-Gal-Trans. In addition to subcellular localization, expression levels of the reporter protein were monitored by staining with anti-HA antibodies.

**Fig. 2:**
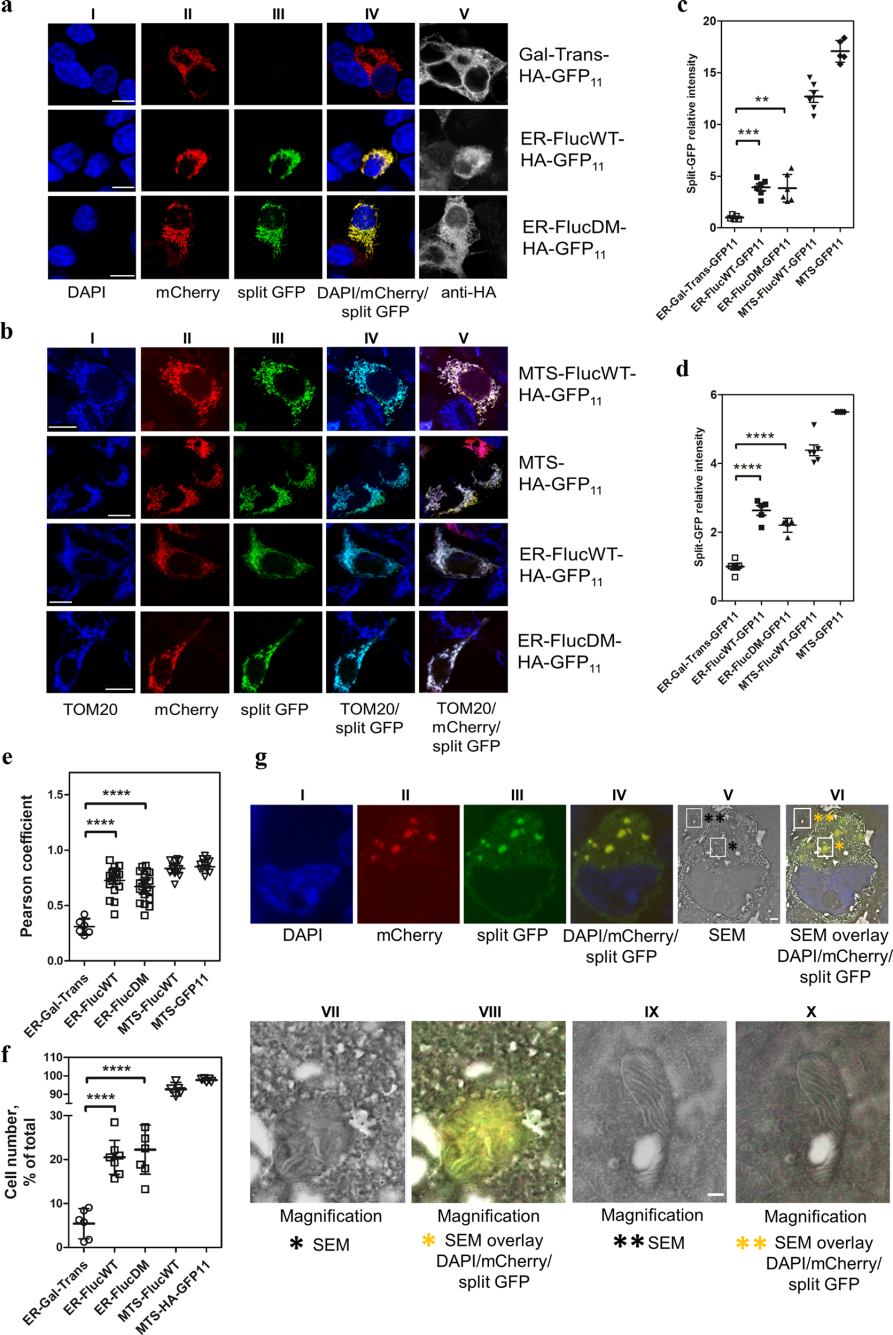
Association of misfolded ER-proteins with mitochondria. Cells were co-transfected with MTS-mCherry-GFP_1–10_ and the indicated HA-tagged GFP_11_ vectors: ER-Gal-Trans-GFP_11_ (control ER-resident protein), ER-FlucWT-GFP_11_, ER-FlucDM-GFP_11_, MTS-GFP_11_ (positive control), MTS-FlucWT-GFP_11_ (positive control). **a** Split-GFP signals for Gal-Trans-GFP_11_, ER-FlucWT-GFP_11_, and ER-FlucDM-GFP_11_. **I—**DAPI, blue; **II—**mCherry, red; **III—**split-GFP, green; **IV—**overlay I), II) and III), co-localized signal – yellow, **V—**anti-HA-tag, white. **b** Split-GFP signal for MTS-FlucWT-GFP_11_, MTS-GFP_11,_ ER-FlucWT-GFP_11_, and ER-FlucDM-GFP_11_. **I—**anti-TOM20, blue; **II—**mCherry, red; **III—**split-GFP, green; **IV—**overlay I) and III), co-localized signal turquois; **V—**overlay I), II), and III), co-localized signal white. Scale bar = 10 µm. **c** Quantification of split-GFP signal associated with mitochondria by confocal microscopy. Normalized split-GFP/mCherry ratio relative to protein expression (median fluorescence ± SEM; *N* ≥ 5). *N* = number of independent transient transfections; for each transfection three coverslips were analyzed. **d** Quantification of split-GFP signal associated with mitochondria by FACS analysis. Normalized split-GFP/mCherry ratio (median fluorescence ± SEM; N = 5). *N* = number of independent transient transfections; for each transfection three technical replicates were done. **e** Pearson’s correlation coefficients for co-localization analysis of split-GFP and MTS-mCherry fluorescence signals. The average Pearson’s correlation coefficients ± SEM were calculated from >10 randomly selected regions with transfected cells (>100 cells/region) in each group. Representative examples of microscopy areas used for calculations are shown in Supplementary. Fig. 6. **f** Quantification of percentage of cells showing split-GFP co-localized with MTS-mCherry signal related to total number of cells expressing both HA-tag and mCherry. Median ± interquartile range. Total 573, 712, 706, and 487 HA/mCherry expressing cells were counted from three different experiments. **p < 0.01, ***p < 0.001, ****p < 0.0001. **g** Correlative light and electron microscopy images of ultrathin sections from cells co-transfected with MTS-mCherry-GFP_1–10_ and ER-FlucDM-HA-GFP_11_ vectors. **I–IV:** Representative images acquired with wide-field microscopy. **I—**DAPI, blue; **II—**mCherry, red; **III—**split-GFP, green**, IV—**overlay of I), II), and III), co-localized signal for red and green-yellow; **V:** Scanning electron microscope (SEM) image from the identical ultrathin section as in I–IV. **VI** – overlay of IV) and V). **VII–X:** magnification of mitochondria of SEM images indicated in V) and VI). Two areas were chosen for magnification, split-GFP / mCherry positive mitochondria (*) and split-GFP / mCherry negative mitochondria (**). VII –* magnification of mitochondria positive for split-GFP **/** mCherry (SEM); VIII -* magnification of mitochondria positive for split-GFP **/** mCherry (overlay SEM with split-GFP / mCherry / DAPI), IX -** magnification of mitochondria negative for split**-**GFP / mCherry (SEM); X -** magnification of mitochondria negative for split**-**GFP / mCherry (overlay SEM with split-GFP / mCherry / DAPI). Scale bar: for **I–VI**: 1000 nm, for **VII–X**: 200 nm.

The positive controls MTS-HA-GFP_11_ and MTS-Fluc-HA-GFP_11_ localized exclusively to mitochondria and cells co-expressing MTS-HA-GFP_11_ or MTS-Fluc-HA-GFP_11_ together with MTS-mCherry-GFP_1–10_ showed a prominent split-GFP signal (Fig. 2b, for quantification Fig. 2c, d). The misfolding ER reporters ER-Fluc-WT-HA-GFP_11_ and ER-FlucDM-HA-GFP_11_ showed a split-GFP signal of varying intensity in cells co-expressing MTS-mCherry-GFP_1–10_. In contrast, the non-misfolding ER reporter ER-Gal-Trans-HA-GFP_11_ showed only a minor, if any, split-GFP signal (Fig. 2a, b, for quantification Fig. 2c, d). These data were further corroborated using Pearson’s correlation coefficient for co-localization analysis of split-GFP and MTS-mCherry fluorescent signals (Fig. 2e). To control for the transfection and expression efficiency of the reporters, we used antibodies specific to the HA-tag, and quantified the percentage of cells showing co-localization of split-GFP with MTS-mCherry signals relative to the total number of cells expressing both HA-tag and mCherry. This revealed that for the positive controls MTS-HA-GFP_11_ and MTS-Fluc-HA-GFP_11_ more than 90% of cells co-expressing MTS-HA-GFP_11_ or MTS-Fluc-HA-GFP_11_ together with MTS-mCherry-GFP_1–10_ showed a split-GFP signal. In cells co-expressing one of the misfolding ER-Fluc-HA-GFP_11_ reporters together with MTS-mCherry-GFP_1–10_ we observed 15–20% split-GFP positive cells. In contrast, very little, if any, cells expressing split-GFP were found among cells co-expressing the non-misfolding ER-Gal-Trans-HA-GFP_11_ reporter together with MTS-mCherry-GFP_1–10_ (Fig. 2f, for illustration see low magnification images Supplementary Fig. 6). Together, the data from the split-GFP experiments support the conclusions from the cell fractionations that, in contrast to the non-misfolded ER reporter protein ER-Gal-Trans, a significant fraction of the misfolded ER reporter protein ER-Fluc becomes associated with mitochondria.

To further demonstrate the mitochondrial localization of the split-GFP signal and to exclude aberrant mislocalization of MTS-mCherry-GFP_1–10_, we used staining with the mitochondrial marker TOM20. The mitochondrial split-GFP signal of the positive controls, MTS-HA-GFP_11_ and MTS-Fluc-HA-GFP_11_, co-localized with TOM20 and mCherry fluorescence, as did the split-GFP signal of ER-FlucWT-GFP_11_ and ER-FlucDM-GFP_11_ (Fig. 2b). Compared to ER-FlucWT, the split-GFP fluorescence was not further increased by the DM mutations as assessed by fluorescence microscopy quantification and FACS analysis (Fig. 2c, d). This observation indicates that misfolding of the FlucWT protein inside the ER is not further aggravated by the additional DM mutations.

We finally used a correlative light and electron microscopy approach in cells co-transfected with ER-FlucWT-GFP_11_ and MTS-mCherry-GFP_1–10_ to demonstrate the localization of split-GFP and mCherry within mitochondria^16^. Both fluorescent signals (mCherry, split-GFP) co-localized and were found within mitochondria as revealed by images of ultrathin sections (110 nm) acquired from correlative light and electron microscopy (Fig. 2g). These data largely exclude the possibility that the co-localization of split-GFP and mitochondrial-targeted mCherry observed by confocal microscopy in cells co-transfected with ER-FlucWT-GFP_11_ and MTS-mCherry-GFP_1–10_ is a mere result of the association of ER-Fluc with the mitochondrial surface. Rather, these data demonstrate that the co-localization of the split-GFP signal with mCherry is at least in part a result of localization of ER-Fluc within mitochondria.

### Expression of ER-Fluc impairs mitochondrial function

**Fig. 3:**
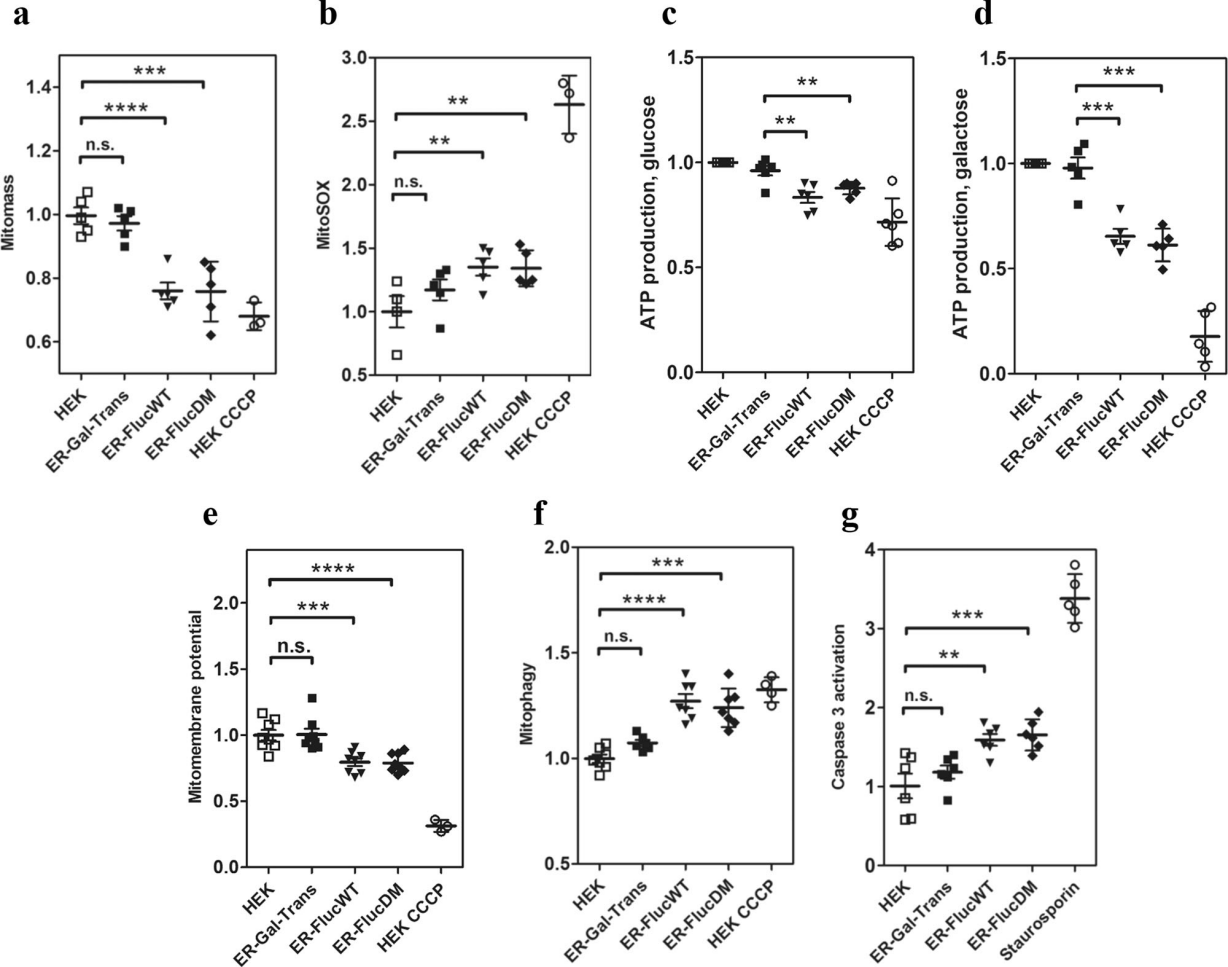
Mitochondrial markers in ER-Fluc expressing cells. **a** Mitochondrial mass measured by Mitotracker Deep Red/FACS (mean fluorescence ± SEM; *N* = 5). **b** Mitochondrial ROS formation measured by MitoSOX/FACS (mean fluorescence ± SEM; *N* = 5). **c** Cellular ATP production assessed by colorimetric/fluorometric assay (mean fluorescence ± SEM; *N* = 6). Cells were incubated in DMEM supplemented with glucose, 4.5 g/L and FBS, 10%. **d** ATP production in cells with suppressed glycolysis (mean fluorescence ± SEM; *N* = 5). Cells were incubated in DMEM supplemented with galactose, 2.5 g/L and no FBS). **e** Mitochondrial membrane potential measured by indicator DilC1(5)/FACS (mean fluorescence ± SEM; *N* = 8). **f** Mitophagy measured by pKeimaRed co-transfection followed by FACS (mean fluorescence ± SEM; *N* = 5). **g** Apoptosis marker – Caspase 3 – assessed by CellEvent Caspase 3/7 cytometry assay/FACS (mean fluorescence ± SEM; *N* = 5). *p < 0.05, **p < 0.01, ***p < 0.001, ****p < 0.0001. *N* - number of independent transient transfections; for each transfection 2 technical replicates were done. Negative control: cells transfected with ER-Gal-Trans; positive control: cells treated with CCCP or staurosporin for 2–4 h. See Supplementary Fig. 15A–E for raw data.

We next investigated whether the accumulation of misfolded ER-Fluc (ER-FlucWT, ER-FlucDM) in mitochondria affects mitochondrial function compared to transfection with the non-misfolding ER-reporter ER-Gal-Trans. For most of these assays, treatment with the ionophore CCCP served as positive control. We first assessed total mitochondrial content using staining with Mitotracker Deep Red. Decreased Mitotracker DR staining indicated a reduced mitochondrial mass in the ER-Fluc transfected cells (Fig. 3a). We also observed that ROS formation, as assessed by FACS analysis of cells stained with MitoSOX Red, was significantly elevated (Fig. 3b). Mitochondrial membrane potential was determined using the red fluorescence indicator DilC1(5) and found to be decreased in cells expressing ER-Fluc (Fig. 3e). Further, ATP content was significantly lower in cells expressing ER-FlucWT or ER-FlucDM (Fig. 3c). We next shifted the cellular ATP synthesis from glycolysis to oxidative phosphorylation by replacing glucose with galactose in the growth media^17^. Replacing glucose with galactose augmented the decrease in ATP content in both ER-Fluc transfected cells and in wild-type cells treated with CCCP (Fig. 3d, Supplementary Fig. 7). Alongside these changes in mitochondrial mass, ROS production, membrane potential, and ATP content we found significantly increased mitophagy in ER-Fluc transfected cells. Mitophagy was assessed using co-transfection with pKeimaRed vector followed by FACS analysis (Fig. 3f). Elevated levels of ROS and mitophagy were further accompanied by increased apoptosis, indicated by increased Caspase 3 activity (Fig. 3g). In contrast, in cells expressing the non-misfolding ER-Gal-Trans reporter none of the markers used to assess mitochondrial function (mitotracker, mitoSOX, membrane potential, ATP content, ATP content in the presence of galactose, mitophagy, Caspase 3, for original data underlying the graphs in Fig. 3 see Supplementary Fig. 15A–E) were significantly different from the wild-type HEK293 controls.

### Mutant form of α-1-antitrypsin becomes sequestered with mitochondria

Our findings with the misfolded ER protein reporter ER-Fluc prompted us to search for endogenous misfolded ER-proteins which could possibly become directed to mitochondria. α-1-antitrypsin (AT) is one of the most abundant serum glycoproteins produced and secreted by the liver. In α-1-antitrypsin (α1AT) deficiency, an E342K mutant of α1AT, termed ATZ, is retained in the endoplasmic reticulum of liver cells rather than secreted. The substitution E342K results in abnormal folding of the mutant protein and frequently comes along with liver injury and hepatocellular carcinoma. In addition, the mutant protein has an increased propensity to polymerize, forming insoluble aggregates within the rough ER^18^.

**Fig. 4:**
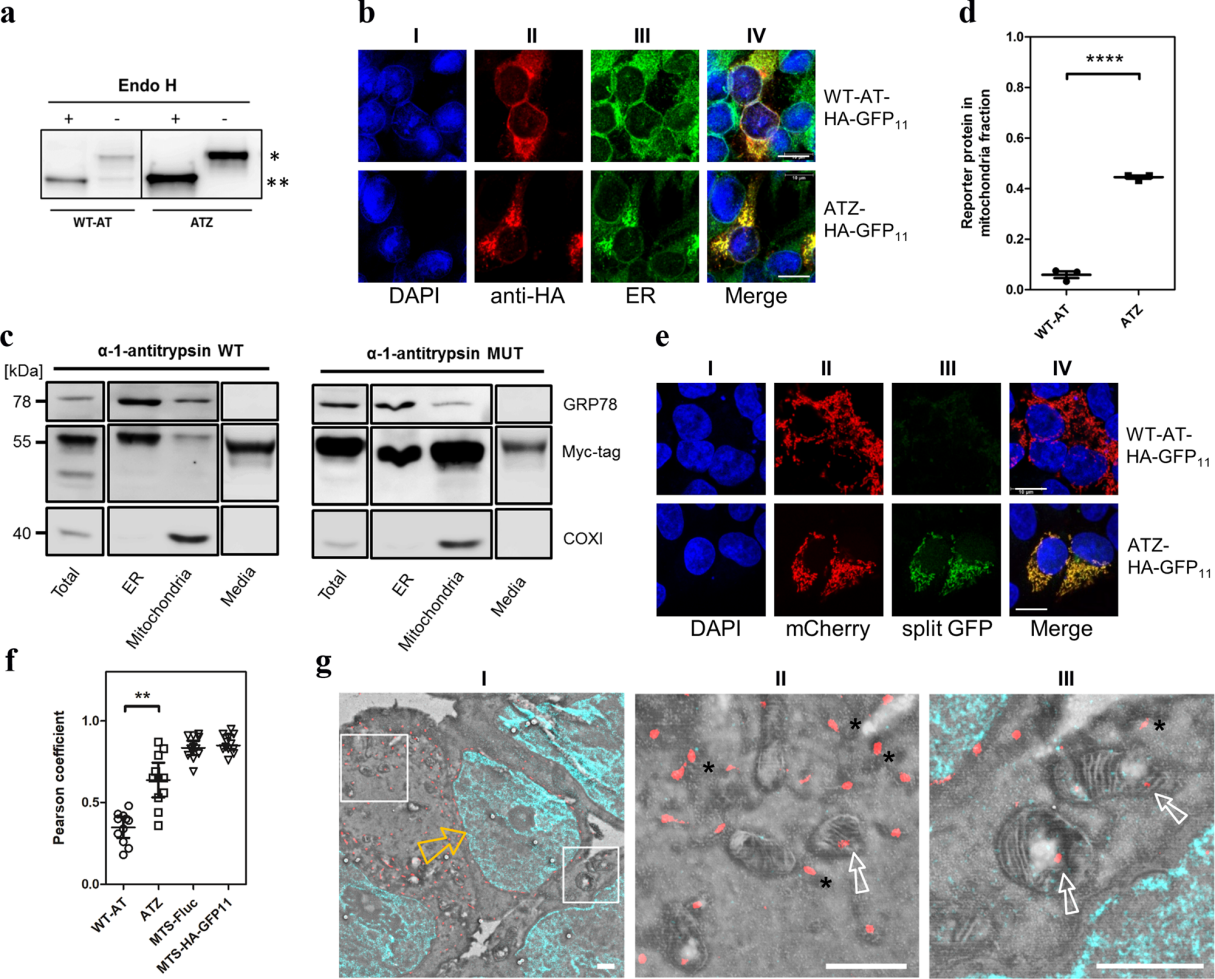
Mitochondrial localization of mutant α1-antitrypsin. **a** Representative Western blot of α-1-antitrypsin-WT (WT-AT) and α-1-antitrypsin mutant (ATZ) samples treated with endoglycosidase H. * = untreated, glycosylated WT-AT and ATZ. ** = upon treatment with Endo H, α-1-antitrypsin is found in its non-glycosylated form for both WT-AT and ATZ. **b** Confocal microscopy image of HEK293 cells expressing WT-AT and ATZ to demonstrate ER localisation. **I** – DAPI, blue; **II** – anti-HA-tag, red; **III** – anti-calreticulin (ER-marker), green; **IV** – overlay I), II), and III), co-localized signal yellow. **c** Representative Western blot of total cellular lysate, ER, mitochondrial and growth medium fractions isolated from HEK293 cells expressing myc-tagged WT-AT and ATZ. GRP78 and COX1 were used as markers for ER and mitochondria, respectively. **d** Semiquantitative assessment of WT-AT and ATZ present in isolated mitochondrial fractions. (*N* = 3). Reporter proteins (WT-AT, ATZ) were normalized to COXI and their recovery in mitochondrial fraction relative to total calculated using the formula: $\frac{{{{{{\rm{reporter}}}}}}\,{{{{{\rm{protein}}}}}}\,{{{{{\rm{mitochondrial}}}}}}\,{{{{{\rm{fraction}}}}}}}{{{{{{\rm{COXI}}}}}}\,{{{{{\rm{mitochondrial}}}}}}\,{{{{{\rm{fraction}}}}}}}:\frac{{{{{{\rm{reporter}}}}}}\,{{{{{\rm{protein}}}}}}\,{{{{{\rm{total}}}}}}}{{{{{{\rm{COXI}}}}}}\,{{{{{\rm{total}}}}}}}$, (see Supplementary Fig. 8). ****p < 0.0001. **e** Split-GFP assay confocal microscopy image of HEK293 cells expressing GFP_11_-tagged WT-AT and ATZ together with MTS-mCherry-GFP_1–10_ to demonstrate mitochondrial localisation of ATZ. **I** – DAPI, blue; **II** – MTS-mCherry, red; **III** – split-GFP, green; **IV** – overlay I), II) and III), co-localized signal yellow. N - number of independent experiments. Scale bar = 10 µm. **f** Pearson’s correlation coefficients for co-localization analysis of split-GFP and MTS-mCherry. The average Pearson’s correlation coefficients ± SEM were calculated from >10 randomly selected regions with transfected cells (>100 cells/region) in each group. MTS-Fluc-GFP_11_ and MTS-GFP_11_ were used as positive controls. **p < 0.01. **g** Correlative light and electron microscopy images of ultrathin sections from HEK293 cells expressing ATZ-Myc. **I** - overlay of representative images acquired with scanning electron microscopy and super-resolution radial fluctuations (SRRF) microscopy: DAPI – blue, anti-Myc-tag (ATZ) – red, the open yellow arrow points at the nucleus. **II** and **III** – magnification of selected regions in **I**, demonstrating mitochondrial (open white arrow) and ER (asterisk) localization of ATZ. Scale bar: for **I**, **II** and **III**: 1000 nm. See Supplementary Fig. 17A–C for uncropped gel scans.

To examine a possible mitochondrial localization of misfolded ATZ, we cloned WT-AT and ATZ fused to a myc-tag in an expression vector and transiently transfected HEK293 cells with these constructs. Treatment of total cell lysates with endoglycosidase H followed by Western blot analysis showed that both WT-AT and ATZ are almost completely glycosylated (Fig. 4a), testifying to ER processing. Visualization of the WT-AT and ATZ reporters by confocal microscopy demonstrated ER localization of both WT-AT and ATZ (Fig. 4b). Fractionation experiments followed by Western blot analysis showed the presence of WT-AT and ATZ proteins in ER fractions, as well as in the growth media, where they were secreted. As expected, mutant ATZ is secreted with less efficiency compared to WT-AT. Most notably, mutant ATZ—but not WT-AT—extensively accumulated in the mitochondrial fraction (Fig. 4c), for quantification see Fig. 4d and Supplementary Fig. 8. Treatment of mitochondrial fractions isolated from ATZ expressing cells with proteinase K either alone or in combination with hypotonic shock (20 mM HEPES) revealed that the majority of misfolded ATZ is subject to proteinase K digestion, with a limited amount of ATZ being protected both from treatment with proteinase K and a combined hypotonic shock/proteinase K treatment (Supplementary Fig. 9). To visualize mitochondrial localization, cells were co-transfected with GFP_11_ tagged WT-AT or ATZ together with MTS-mCherry-GFP_1–10_. This demonstrated significant mitochondrial GFP fluorescence for cells expressing ATZ, but only a weak, if any, split-GFP signal for cells expressing WT-AT (Fig. 4e). These data were corroborated by calculation of the Pearson’s correlation coefficient which was used to quantify the co-localization of split-GFP and MTS-mCherry signals for WT-AT and ATZ compared to the positive controls MTS-HA-GFP_11_ and MTS-Fluc-GFP_11_ (Fig. 4f).

We wished to corroborate the confocal microscopy results obtained with the split-GFP system by direct tagging of ATZ with a fluorescent protein. Transfection of HEK293 with an ATZ reporter labelled with BFP showed co-localization of BFP with both mitochondrial reporter MTS-mCherry and mitochondrial marker TOM20 (Supplementary Fig. 10A). Further studies combining the BFP-tag with the split-GFP system using a double BFP- and HA-tagged ATZ GFP_11_ reporter and MTS-mCherry-GFP_1–10_ together with staining of the mitochondrial marker TOM20 demonstrated that while the majority of BFP-labelled ATZ was localized outside mitochondria, i.e., in the ER, a significant part of BFP-labelled ATZ showed a split-GFP signal, which co-localized with TOM20 and MTS-mCherry. Similar results were seen when staining the HA-tag of ATZ GFP_11_ with HA-antibodies (Supplementary Fig. 10C). For more precise localization of transfected ATZ, we combined immunolabelling with electron microscopy (EM) in ultrathin sections of HEK cells transfected with ATZ-myc vector. EM-immunolabelling using anti-myc antibodies revealed the presence of ATZ inside mitochondria, in addition to the expected majority of ATZ being associated with ER (Fig. 4g).

### Mitochondrial localization of misfolded ER-Fluc in yeast *Saccharomyces cerevisiae* requires ERMES

**Fig. 5:**
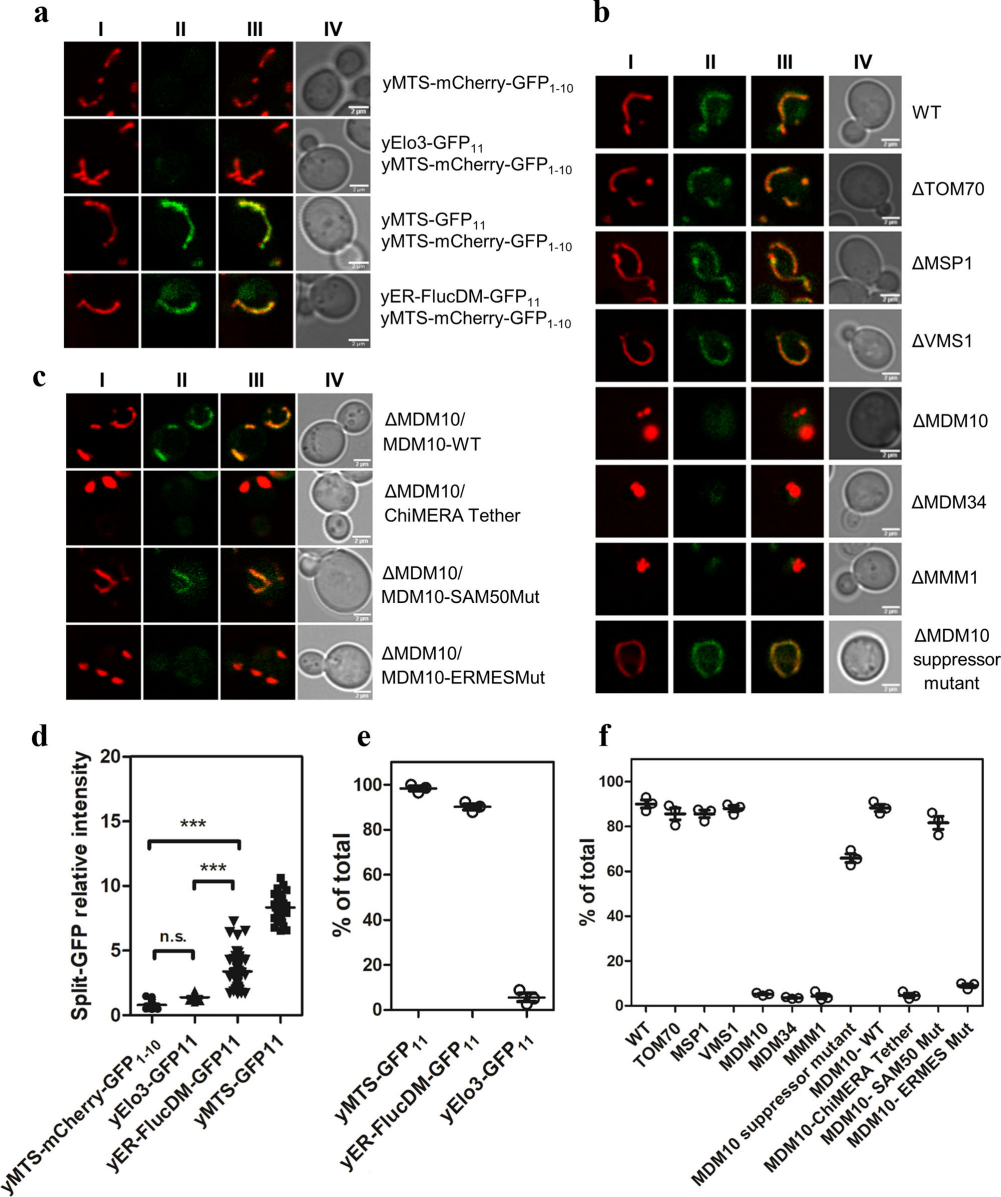
Localization of yER-FlucDM in yeast *Saccharomyces cerevisiae*. **a** Representative confocal images showing co-localization of yER-FlucDM with mitochondria. Cells expressing yMTS-mCherry-GFP_1–10_ together with indicated GFP_11_ construct; yMTS-GFP_11_ (positive control), yElo3-GFP_11_ (negative control), yER-FlucDM-GFP_11_. **I** – yMTS-mCherry, red; **II** – split-GFP, green; **III** – overlay I) and II), co-localized signal is shown in yellow, **IV** – bright field. Scale bar = 2 µm. **b** Representative confocal images showing co-localization of yER-FlucDM with mitochondria. Cells expressing yER-FlucDM-GFP_11_ and yMTS-mCherry-GFP_1–10_ were subjected to the indicated targeted gene deletions, i.e. ΔΤΟΜ70, ΔMSP1, ΔVMS1, ΔMDM10, ΔMDM34, ΔMMM1, and ΔMDM10 adapted suppressor mutant. **I** – yMTS-mCherry, red; **II** – split-GFP, green; **III** – overlay I) and II), co-localized signal is shown in yellow, **IV** – bright field. Scale bar = 2 µm. **c** Representative confocal images showing co-localization of yER-FlucDM with mitochondria. Deletion mutants of MDM10 (ΔMDM10) expressing yER-FlucDM-GFP_11_, and yMTS-mCherry-GFP_1–10_ were complemented with MDM10-WT (ΔMDM10/MDM10-WT), artificial ChiMERA tether (ΔMDM10/ChiMERA Tether), MDM10-SAM50 mutant (ΔMDM10/MDM10-SAM50 Mut), and MDM10-ERMES mutant (ΔMDM10/MDM10-ERMES Mut). **I** – yMTS-mCherry, red; **II** – split-GFP, green; **III** – overlay I) and II), co-localized signal is shown in yellow, **IV** – bright field. Scale bar = 2 µm. **d** Quantification of normalized split-GFP/mCherry ratio by confocal microscopy in cells transfected with yMTS-mCherry-GFP_1–10_ and indicated construct. Median ± interquartile range; 23, 23, 48, 33 frames quantified (*N* = 4, number of independent experiments). Mann–Whitney two-tailed test, non-parametric. ***p < 0.001. **e** Quantification of the percentage of cells showing co-localization of mCherry and split-GFP in WT cells transfected with yMTS-mCherry-GFP_1–10_ and indicated construct. Median ± interquartile range. Total 217, 253, 204 cells were counted from three different experiments. **f** Quantification of percentage of cells showing co-localization of mCherry and split-GFP in deletion mutants expressing yMTS-mCherry-GFP_1–10_ and yER-FlucDM-GFP_11_. Median ± interquartile range. Total of 230, 205, 227, 300, 134, 112, 126, 134, 126, 125, 140, 166 cells were counted from three independent experiments.

To study whether mitochondrial sequestering of misfolded ER proteins is evolutionary conserved, we used split-GFP constructs adapted to the yeast system. To target Fluc to the ER, the signal peptide of the native yeast ER protein KAR2 was placed upstream and the HDEL ER-retention sequence downstream of luciferase resulting in yeast yER-FlucDM-GFP. Co-transformation of yER-FlucDM-GFP with yElo3-mCherry, Elo3 is a yeast ER-resident protein, revealed localization of yER-FlucDM-GFP in the ER (Supplementary Fig. 11)^19^. Compared to the homogeneous yElo3-mCherry signal, the yER-Fluc signal additionally revealed irregular blobs indicative of protein aggregates. For the split-GFP assay, performed to assess localization in mitochondria, the yER-FlucDM-GFP_11_ fusion protein was used as a reporter and yMTS-GFP_11_ and yElo3-GFP_11_ were used as positive and negative controls respectively. The reporters were co-transformed along with yMTS-mCherry-GFP_1–10_. Mitochondria-associated GFP fluorescence was visualized and quantified using confocal microscopy. Yeast cells transformed with yMTS-mCherry-GFP_1–10_ were used as a control for autofluorescence. Confocal microscopy demonstrated absence of a mitochondrial split-GFP signal for cells expressing the GFP_11_-tagged ER-resident protein yElo3-GFP_11_, but a prominent mitochondrial split-GFP signal for yER-FlucDM-GFP_11_, indicating trafficking of the misfolded ER reporter to mitochondria (Fig. 5a, d and e).

Mitochondria have specialized surveillance systems, which target faulty proteins designed for mitochondrial import. MitoCPR (Compromised Protein Import Response) removes stalled precursor proteins accumulated at the mitochondrial outer membrane, while VMS1 is part of a mitochondrial quality pathway that acts on mitochondrial polypeptides stalled on ribosomes at the mitochondrial surface^20,21^. Neither deletion of mitoCPR components MSP1 and TOM70 or of VMS1 in yeast co-expressing yER-FlucDM-GFP_11_ and yMTS-mCherry-GFP_1–10_, measurably affected the intensity of the split-GFP signal or its co-localization with the mitochondrial mCherry marker (Fig. 5b, f). Together this suggests that classical quality control pathways acting on faulty proteins designed for mitochondrial import do not affect trafficking of yER-FlucDM to mitochondria.

ER and mitochondria form physical contact sites that facilitate the cross-talk between these two organelles. In yeast, a protein complex termed endoplasmic reticulum-mitochondria encounter structure (ERMES) has been identified which tethers ER and mitochondria^9^. Deletion of MDM10, an integral mitochondrial membrane protein and structural component of ERMES, lead to the previously shown alteration in mitochondrial morphology with the characteristic tubular mitochondrial network being replaced by condensed mitochondria (Fig. 5b)^22,23^. Staining with MitoTracker Green confirmed these condensed structures as mitochondria (Supplementary Fig. 12). Notably, deletion of MDM10 in cells expressing yER-FlucDM-GFP_11_ together with yMTS-mCherry-GFP_1–10_ completely abrogated the split-GFP signal associated with mitochondria. In addition to MDM10, deletion of further core components of the ERMES structure, i.e., MMM1 and MDM34, in cells expressing yER-FlucDM-GFP_11_ together with yMTS-mCherry-GFP_1–10_, besides resulting in a condensed mitochondrial structure, abrogated the split-GFP signal associated with mitochondria (Fig. 5b, f). Western blots were used to corroborate the expression of yER-FlucDM-GFP_11_ and yMTS-mChery-GFP_1–10_ in the yeast deletion mutants (Supplementary Fig. 13). None of the genetic deletions affected the split-GFP signal of the positive control yMTS-GFP_11_ co-transformed with yMTS-mCherry-GFP_1–10_.

The artificial ER-mitochondria tether ChiMERA^9^ has been used previously to compensate for the tethering defects associated with ERMES mutants. We expressed the ChiMERA tether in MDM10 mutants and controlled expression by Western blot (Supplementary Fig. 14). In contrast to complementation with MDM10 (see below), MDM10 deletion mutants expressing yER-FlucDM-GFP_11_ and yMTS-mCherry-GFP_1–10_ and transformed with the ER-mitochondria tether ChiMERA showed continued absence of the mitochondrial split-GFP signal together with condensed mitochondria (Fig. 5c, f). Besides recapitulating the observation that ChiMERA cannot rescue mitochondrial morphology in MDM10 mutants^9^, this finding indicates that expression of the ChiMERA tether in MDM10 mutants is insufficient for translocation of misfolded ER-Fluc into mitochondria. ERMES mutants frequently lose their phenotype over time^24^ due to appearance of suppressor alleles in the endosomal vascular protein sorting 13 (VPS13), which suppress the phenotypic consequences of ERMES deficiency^25^. We wondered whether this suppression may extend to the mitochondrial split-GFP signal absent in MDM10 mutants expressing yER-FlucDM-GFP_11_ together with yMTS-mCherry-GFP_1–10_. Indeed, over time we observed adapted MDM10 mutants, which showed restoration of normal mitochondrial morphology together with rescue of the split-GFP signal (see Fig. 5b and f). Sequencing of the VPS13 gene in two adapted MDM10 mutants revealed that adaptation was associated with dominant VPS13 mutations known to restore mitochondrial morphology in ΔMDM10 mutants, *i.e*., D716Y and G820R^25,26^.

In addition to ERMES, MDM10 is part of the mitochondrial sorting and assembly machinery (SAM). Point mutants of MDM10 have been used to separate the function of MDM10 in the biogenesis of outer membrane proteins and protein assembly (SAM) from its function in morphology and phospholipid homeostasis (ERMES)^22^. We employed these point mutants to address the role of MDM10 in mitochondrial localization of misfolded ER-Fluc. Expression levels of SAM mutant MDM10 and ERMES mutant MDM10 were controlled by Western blot (Supplementary Fig. 14). Complementation of MDM10 deletion mutants expressing yER-FlucDM-GFP_11_ and yMTS-mCherry-GFP_1–10_ with wild-type MDM10 or dysfunctional SAM mutant MDM10 resulted in recovery of the mitochondrial split-GFP signal along with restored tubular mitochondrial morphology. In contrast, cells complemented with dysfunctional ERMES mutant MDM10 showed continued absence of the mitochondrial split-GFP signal together with condensed mitochondria (Fig. 5c, f). These results suggest that interaction between ER and mitochondria through ERMES is essential for mitochondrial sequestration of misfolded ER-Fluc.

## Discussion

In general, the formation and organization of protein aggregates is not random. Recently, misfolded cytosolic proteins have been reported to accumulate at ER-mitochondria contact regions, where they eventually become transported into mitochondria^14,15,27–29^. The findings we describe here add an additional layer to the recently proposed role for mitochondria in proteostasis. Using two different reporters, ER-Fluc and mutant α-1-antitrypsin, and a combination of cell fractionation, biochemistry, confocal microscopy, FACS, and electron microscopy studies, we here demonstrated that misfolded aberrant proteins formed in the ER can become associated with mitochondria and partially imported into the mitochondrial matrix, resulting in impaired mitochondrial function. Demonstration of this trafficking pathway for misfolded ER proteins both in mammalian cells and in yeast, while suggesting that mitochondrial sequestration of aberrant ER proteins is evolutionary ancient, also allowed us to in part address mechanistic questions.

Inactivation of mitochondrial surveillance pathways which act on import of faulty mitochondrial proteins by genetic deletion of MSP1, TOM70, and VMS1 did not affect trafficking of the ER proteins into mitochondria. However, we found that MDM10, MMM1 and MDM34, all components of ERMES-mediated ER-mitochondria contacts, are essential for this pathway. Multiple mitochondrial functions have been linked to ERMES, including biogenesis of outer membrane proteins, lipid homeostasis, membrane dynamics and maintenance of mitochondrial morphology^22,30^. In addition to being a part of ERMES, MDM10 is also present in SAM complexes. The MDM10-SAM interaction is required for biogenesis of the mitochondrial outer membrane transporters TOM22 and TOM40^22,23^. Using mutants of MDM10, which separate the MDM10-ERMES function from the MDM10-SAM function, we show that the mitochondrial sequestration of misfolded ER-Fluc is critically dependent on the ERMES function of MDM10. Our data further show that this function of ERMES can be bypassed by dominant mutations in the conserved eukaryotic endosomal protein VPS13.

The involvement of mitochondria in the pathogenesis of AT-deficiency with mutant gain-of-function α1-AT has been a mystery, despite detailed studies of liver pathology in the PiZ mouse model demonstrating significant mitochondrial damage and mitochondrial injury, as well as caspase-3-induced apoptosis in situ^31^. Our investigations suggest that aberrantly folded ATZ in the ER becomes associated with mitochondria. While our fractionation experiments cannot separate mitochondria-associated ATZ from ATZ possibly accumulating in inclusions of ER membrane or turned over by ER-phagy, our imaging experiments using confocal microscopy and a combined light and electron microscopy approach demonstrate that at least part of misfolded ATZ becomes associated with the mitochondrial matrix.

Our findings have clear implications for further understanding the pathogenesis of protein misfolding disorders. There is compelling evidence for two apparently unrelated phenomena in neurodegenerative diseases (NDDs): ER stress and unfolded protein response *versus* mitochondrial dysfunction^32–35^. A number of pathogenic disease-specific rogue proteins, e.g. Aβ in Alzheimer’s disease (AD), α-synuclein in Parkinson’s disease, either pass through the ER or have been shown to accumulate as oligomers or aggregates inside the ER^36–38^. ER stress may affect mitochondrial function through interorganelle signalling^39–41^. However, the herein described role for mitochondria in sequestering ER-misfolded proteins hints to a possible direct structural link connecting protein misfolding in ER and mitochondrial dysfunction. NDDs have recently been proposed to be disorders of ER-mitochondria connectivity^42^. E.g., some features of AD, particularly those manifesting early in the disease process, are apparently unrelated to Aβ plaque and tangle formation, but imply functions related to mitochondria-associated membranes (MAMs)^43^. The missing mechanistic link in this hypothesis was the cause for perturbations in ER-mitochondrial apposition. Our results, which demonstrate that ER-misfolded proteins are sequestered by mitochondria and that this sequestration requires the ERMES function of ER-mitochondrial contact sites, provide a possible answer to this question.

While we realize the many unknowns and the mechanistic details which remain to be elucidated in this mitochondrial sequestration of glycosylated ER-misfolded proteins, we note that it is not without precedence. Most notably, previous reports largely unrecognized by the field have described the mitochondrial import of glycoproteins previously processed in the endoplasmic reticulum^44,45^. In summary, our data provide evidence of a novel degradation pathway for ER-misfolded proteins which become sequestered with mitochondria, highlighting a novel mechanism of cellular proteostasis. We term this mitochondria-associated proteostatic mechanism for ER-misfolded proteins ERAMS, for ER-associated mitochondrial sequestration.

## Materials and methods

### Luciferase assay

Enzymatic activity of Fluc was determined using the Luciferase Assay System (Promega) as recently described^14^. In brief, HEK293 cells were lysed, the different fractions (total, cytosol, ER, and mitochondria) obtained and their protein content measured using Micro BCA Protein Assay Kit (Thermo Scientific). Equal protein amount was incubated with Passive Lysis Buffer (total volume 20 μl/well in 96 well plate) for 15 min at RT, mixed with 100 μl of Luciferase Assay Reagent and bioluminescence was measured using plate reader Cytation 5 (BioTek). The specific luciferase activity was calculated by relating the enzymatic activity of each fraction to the corresponding band intensity quantification by Western blot.

### Cell culture and preparation of organelle-enriched fractions

HEK293 cells cultivated in DMEM supplemented with 10% FBS were harvested, resuspended in MTE-P buffer (270 mM D-mannitol, 10 mM Tris base, 0.1 mM EDTA, pH = 7.4, supplemented with 1 mM PMSF), and homogenized using a Dounce homogenizer. Homogenates were centrifuged at 1400 × *g* for 10 min at 4 °C to remove cellular debris. An aliquot of supernatant was kept as total cellular protein fraction, the remaining supernatant was centrifuged at 15,000 × *g* for 10 min at 4 °C, resulting in supernatant A and pellet B.

For preparation of ER-enriched fractions, supernatant A was loaded on a sterile 14 × 89–mm polyallomer tube (Beckman) containing a sucrose gradient (3 ml 1.3 M sucrose, 3 ml 1.5 M sucrose, 2 ml 2.0 M sucrose; 10 mM Tris base, 0.1 mM EDTA, pH = 7.6) and centrifuged at 152,000 × g for 70 min at 4 °C, acceleration/deceleration = 1. The cytosolic fraction was taken from the topmost sucrose layer, and the interface containing the ER was collected using a 20-G needle and 1 ml syringe. The ER containing fraction was transferred to a 5.0 ml, 13 × 51 mm polyallomer tube (Beckman) and supplemented with additional ice-cold MTE-P to a final volume of 4 ml. Tubes were covered with parafilm, mixed by inversion until the suspension was homogeneous and ultracentrifuged at 126,000 × *g* for 45 min at 4 °C. Supernatant was discarded, the pellet was resuspended in ice-cold MTE-P and used as ER-enriched fraction.

For preparation of mitochondria-enriched fractions, pellet B was washed twice with 0.5 ml of ice-cold MTE-P, resuspended in 0.8 ml of ice-cold MTE-P buffer and loaded slowly on top of a sucrose gradient (1 ml of 1.7 M sucrose overlaid with 1.6 ml of 1.0 M sucrose in a sterile 5.0 ml, 13 × 51 mm Beckman polyallomer tube). The tube was filled up with an additional 0.8 ml of ice-cold MTE-P buffer and centrifuged for 22 min at 40,000 × *g*, 4 °C (20600 rpm in an SW50.1 rotor). The interface between the 1.7 M and 1.0 M sucrose layers was collected (0.4 ml), mixed with 1.1 ml of ice-cold MTE-P buffer and centrifuged for 10 min at 15,000 × *g*, 4 °C. The resulting pellet was resuspended in 100 μl of MTE buffer without PMSF and used as mitochondrial fraction.

### Western blot

Cells were grown to 90% confluency in DMEM with 10% FBS, lysed and fractionated as described above and protein concentration measured using Micro BCA Protein Assay Kit (Thermo Scientific). Fractions were resolved by SDS-PAGE and blotted onto nitrocellulose membrane (Amersham) for Western blot analysis. The specific antibodies used in this study were: polyclonal anti-Myc (Abcam ab9106), rabbit polyclonal anti-Firefly luciferase (Abcam ab21176), rabbit polyclonal anti-Tubulin (Abcam ab6046), Total OXPHOS antibody cocktail (Abcam ab110413), anti-GRP94 (Abcam ab3674), anti-GRP78 (Abcam ab21685), anti-TOM70 (Abcam 89624) and anti-TIM23 (Abcam 116329). Signal intensity of the protein bands was quantified using ImageQuant (GE Healthcare).

### Construction of mammalian vectors and cell transfection

For a list of vectors used see Supplementary Table 1. For mitochondrial targeting, we used the mitochondrial targeting sequence (MTS) from vector MTS-mCherry-GFP_1–10_^15^. For construction of ER-luciferase reporters, we used WT or mutant DM firefly luciferase fused to an N-terminal prolactin signal sequence for ER transport (MNIKGSPWKGSLLLLLVSNLLLCQSVAP) and a C-terminal KDEL retention sequence^15^. Alpha-1-antitrypsin vectors were constructed by amplifying the alpha-1-antitrypsin sequence from pHAGE-EF1alphaL-hAAT-W (Addgene) using primers containing a Myc-tag coding sequence (GAACAAAAACTCATCTCAG AAGAGGATCTG). The alpha-1-antitrypsin and the alpha-antitrypsin-Myc genes were then introduced into the hFluc-GFP_11_ vector replacing hFluc and generating alpha-1-antitrypsin-WT-GFP_11_ and alpha-1-antitrypsin-WT-Myc constructs^15^. The mutant versions of the alpha-1-antitrypsin-WT constructs were obtained by substituting glutamate (GAG) at position 342 with lysine (AAG) by site-directed mutagenesis PCR (Q5^®^ Site-Directed Mutagenesis Kit, NEB). Vector Gal-Trans-Myc-GFP_11_-KDEL was constructed by replacing hFluc with the galactosyltransferase sequence in the PLN-hFluc-GFP_11_-KDEL plasmid; Gal-Trans was amplified from GLT25D1 (COLGALT1) Human Untagged Clone (OriGene) and a Myc-tag was linked by using primers 5’-HindIII-GATCACTAGAAGCTTGGTACCGAGGAGATCTGCC-3’and 3’-RsrII-TGGCGACCGGACCGATCAGATCCTCTTCTGAGATGAGTTTCTGCTCGAGC GGCCGCGTACGCGTGCTGAGTTCATCCCGGGCAGCACTGTCCAGTGGGG-5’. Cells were transiently transfected with corresponding plasmid using X-fect reagent (TaKaRa). Forty-two hours post-transfection cells were harvested for analysis.

### Endo H treatment

Endo H glycosidase (Promega) treatment was done according to the manufacturer’s protocol. 60 μl of total cellular protein extract were denatured at 95 °C for 5 min using denaturing solution, cooled down to room temperature for 5 min, Endo H buffer (Promega) and 2000 units of Endo H glycosidase were added. Samples were incubated for 3 h at 37 °C. The resulting samples were resolved by SDS-PAGE and analyzed by Western blot using HRP-conjugated specific anti-Fluc and anti-Myc antibodies (Abcam). The signal intensities of the Fluc and AAT-Myc bands were quantified using ImageQuant.

### Proteinase K treatment

Mitochondrial fractions were prepared as described above and OD at 600 nm was measured. Mitochondrial fractions were adjusted to OD600 1.0–1.5 and 10 μl volumes were used per reaction. Mitochondria were permeabilized using hypotonic shock with 20 mM HEPES solution or treatment with 2% Triton X-100. After 30 min incubation at 4 °C, proteinase K was added (200 μg/ml final concentration) and incubated for 30 min and 60 min at 4 °C. Input control samples were kept with cold MTE buffer w/o detergents and proteinase K. Finally, 10 μg of insulin (Sigma) were added for protein co-precipitation, followed by TCA precipitation for 30 min at 4 °C (final concentration −20% TCA). Following TCA precipitation samples were centrifuged at 21,000 × g for 20 min at 4 °C, and the resulting pellets were dissolved in 30 μl of 1 M Tris-HCl pH 7.5. Samples were resolved by SDS-PAGE and analyzed by Western blotting. Anti-Fluc and anti-Myc antibodies were used to visualize the target proteins; outer membrane TOM70 antibody, inner membrane TIM23 antibody and matrix COX1 antibody were used to control for mitochondrial integrity and loading.

### Mitochondrial mass measurement

For estimation of mitochondrial mass, Mitotracker™ DeepRed FM (M22426, Invitrogen) staining was used according to manufacturer instructions. Cells transfected with vectors pGal-Trans-eGFP, pER-FlucWT-eGFP or pER-FlucDM-eGFP were stained with Mitotracker™ DeepRed 40–48 h after transfection and analyzed by flow cytometry using the BD FACS Canto II and the FlowJo software. GFP signal was used to gate the transfected cells. Treatment of wild-type HEK293 cells with CCCP (10 µM, 12 h) was used as a positive control.

### Mitochondrial membrane potential measurement

For measurement of mitochondrial membrane potential, far-red fluorescent indicator DilC1(5) (M34151, ThermoFisher) was used, following manufacturer instructions. In brief, cells transfected with vectors pGal-Trans-eGFP, pER-FlucWT-eGFP or pER-FlucDM-eGFP were stained with DilC1(5) 40–48 h after transfection and analyzed by flow cytometry using the BD FACS Canto II and the FlowJo software. GFP signal was used to gate the transfected cells. Treatment of wild-type HEK293 cells with CCCP (20 µM, 1 h) was used as a positive control.

### Mitophagy assay

Mitophagy was assessed using co-transfection with vector pKeimaRed carrying mitochondria-targeted fluorescent protein Keima-Red (Amalgaam MBL AM-V0259M). The fluorescent protein Keima has a pH-dependent excitation spectrum that changes depending on pH. A short excitation wavelength (440 nm) is predominant in a neutral environment, whereas a long excitation wavelength (586 nm) is predominant in an acidic environment. pH change upon fusion of mitophagosome with lysosome leads to corresponding shift of excitation spectrum. Mitophagy was monitored by FACS analysis using the BD FACS Fortessa and the FlowJo data analysis software. Keima-Red fluorescent signal was used to gate transfected cells. A violet laser 405 nm and filter set 610/20 was applied to measure the short excitation wavelength fluorescence (SE-fluo), a yellow-green laser 561 nm and the same filter set was applied to measure the long excitation wavelength fluorescence (LE-fluo). “Mitophagy index” proportional to the percentage of mitochondria undergoing mitophagy was calculated as a LE-fluo to SE-fluo+LE-fluo. Treatment with CCCP (10 µM, 16 h) was used as a positive control for mitophagy.

### Caspase 3/7 activation assay

Caspase 3/7 activity was measured using the CellEvent™ Caspase-3/7 Green Flow Cytometry Assay Kit (ThermoFisher) according to manufacturer instructions. Non-transfected HEK WT cells and cells transfected with vectors pGal-Trans, pER-FlucWT or pER-FlucDM were co-transfected with pTag-BFP, stained with CellEvent™ Caspase-3/7 assay 40–48 h after transfection and analyzed by flow cytometry using the BD FACS Canto II and the FlowJo software. Tag-BFP signal was used to gate the transfected cells. Treatment with staurosporin (1 µM, 6 h) was used as a positive control. The number of the CellEvent™ Caspase-3/7 positive cells in % to all transfected (BFP-positive) cells represents the caspase 3/7 activation level.

### ROS measurements

To detect mitochondria-specific superoxide anion in transfected HEK293 cells, MitoSOX™ Red staining (Life Technologies) was performed in live cells. Non-transfected HEK WT cells and cells transfected with vectors pGal-Trans, pER-FlucWT or pER-FlucDM were co-transfected with pTag-BFP, grown to ~80% confluency and stained with MitoSOX™ Red (5 μM) in FACS buffer (1 x PBS, 2% FBS) for 30 min at 37 °C. Buffer with MitoSOX was removed, cells detached using Accutase and resuspended in FACS buffer. Fluorescence was analyzed by flow cytometry using the BD FACS Fortessa and the FlowJo data analysis software. Tag-BFP signal was used to gate transfected cells. Treatment with CCCP (10 µM, 4 h) was used as a positive control for ROS induction.

### Cellular ATP production

Intracellular levels of ATP were measured using colorimetric/fluorometric ATP assay kit (ab83355, Abcam). Cells transfected with vectors pGal-Trans-eGFP, pER-FlucWT-eGFP or pER-FlucDM-eGFP were harvested, suspended in specific ATP-assay buffer, lysed by shock-freezing and ATP levels determined according to manufacturer instructions using fluorimeter Cytation 5 (BioTek). The level of transfection monitored by FACS using GFP signal as a marker was around 80%. Treatment with CCCP (20 µM, 2 h) was used as a positive control. To assess mitochondrial OXPHOS-dependent ATP production transfected cells were rinsed with PBS and incubated in DMEM medium supplemented with 2.5 g/L galactose (w/o glucose and FBS) for 4–5 h and extensive aeration before harvesting.

### Split-GFP assay

For split-GFP assay HEK293 cells were transiently co-transfected with vectors MTS-mCherry-GFP_1–10_ and one of the following plasmids: pPLN-hFluc-WT-HA-GFP_11_-KDEL, pPLN-hFluc-DM-HA-GFP_11_-KDEL, pAT-WT-HA-GFP_11_, pATZ-HA-GFP_11_, pGal-Trans-Myc-HA-GFP_11_-KDEL (negative control), pMTS-HA-GFP_11_ (positive control), or pMTS-hFluc-HA-GFP_11_ (positive control). 24 h and 48 h after transfection GFP and mCherry fluorescence intensities were assessed by flow cytometry using FACS LSR Fortessa (BD Biosciences) and FlowJo analysis software or by confocal fluorescent microscopy using Leica SP8 confocal microscope with 63x oil-immersion NA = 1.4 objective. For immunofluorescent microscopy cells were fixed with 3% PFA, permeabilized with 0.1% TX100 and stained with anti-HA-tag antibodies (ab137838, rabbit) and/or anti-TOM20 antibodies (ab56783, mouse). Anti-mouse (ab175661, Alexa-405) and anti-rabbit (ab97077, Cy5) antibodies were used as secondary antibody. For quantification 30 randomly selected frames (each covering at least 20 cells) from 3 independent transfection events (10 frames/event) were collected. Microscopic images were processed using ImageJ (NIH) software and quantified with embedded ImageJ function Analyze Particles after segmentation of the images based on mCherry (mitochondrial) signal. GFP signal was calculated within mitochondrial areas and adjusted to protein-of-interest expression level (assessed by HA-tag immunofluorescence). GFP/mCherry ratio was calculated and used as a measure of ER-protein mistargeted to mitochondria. Pearson’s correlation coefficient and Manders’ co-localization coefficient were calculated using ImageJ Coloc2 plug-in.

### Correlative light and electron microscopy

HEK293 cells transiently co-transfected with MTS-mCherry-GFP_1–10_ and ER-FlucDM-HA-GFP_11_-KDEL vectors were fixed with 4% formaldehyde and 0.1% glutaraldehyde in cacodylate buffer 0.1 M. Cells were pelleted by centrifugation, embedded in 12% agarose and cryoprotected with 2.3 M sucrose prior to cryosectioning. Ultrathin (110 nm) sections were mounted on silicon wafers^16^. Wafers were washed with PBS (0.1 M, pH 7.4, 0 °C), followed by incubation with 0.15% glycine in PBS (3 ×1 min), and washed with PBS (3 ×1 min). Dapi (4’, 6-diamidino-2-phenylindole dihydrochloride, Sigma, 1:250) was shortly applied (10 sec) and washed with PBS. For light microscopy, we imaged the ultrathin sections with a 100 × 1.44 NA objective on an inverted wide-field fluorescent microscope (Thunder, Leica microsystem) equipped with LED illumination and a sCMOS camera (DFC9000 GTC, Leica microsystems). For electron microscopy, the silicon wafers were embedded in methylcellulose, centrifuged and mounted on an aluminium stub for imaging by in a scanning electron microscope (Auriga 40, Zeiss) at an acceleration voltage of 0.8 keV using an In Lens secondary electron detector, a pixel dwell time of 100 μs and a working distance of 5 mm. Images were acquired at 4 nm pixel size. Light and electron microscope images were registered and correlated with TrakEM2 within the open-source platform Fiji^46,47^.

HEK293 cells transiently transfected with ATZ-myc vector were processed as described above, with following modifications. Fluorescent heads (PS-Speck, #P7220, ThermoFisher, 1:3 in ethanol absolute) were added as fiducial markers prior to collecting the ultrathin sections on wafers. Following washing, a 5 min pre-incubation with PBG (PBS with 0.5% bovine serum albumin and 0.2% gelatin type B) was followed by incubation with rabbit anti-myc-tag (Abcam ab9106, 1:100) in PBG for 1 h. After washes with PBG (6 ×1 min and 1 ×5 min), wafers were incubated with a secondary antibody anti-rabbit Alexa 568 (Molecular Probes A21069, 1:100) in PBG for 1 h. For SRRF (Super-resolution radial fluctuations) 500 images per channel were acquired at 15 ms exposure time. These images were further processed with the Plugin Nano-J-SRRF^48^ within the open-source platform Fiji^47^. The following parameters were applied: Ring radius: 0.5; Radiality magnification: 7 and Axes in Ring: 7. For electron microscopy, the silicon wafers were processed as described above.

### Yeast strains and plasmids

Plasmids containing split-GFP constructs and Elo3-mCherry were ordered from Addgene and the split-GFP constructs were adapted to the current study by subcloning into p403 and p406 vector backbones under control of the TDH3 promoter^15,19^. yER-FlucDM-GFP_11_ was targeted to ER by cloning native ER protein KAR2 signal sequence (KAR2SS) upstream FlucDM-GFP_11_, HDEL was added downstream for ER retention. yMTS-mCherry-GFP_1–10_ was targeted to mitochondria using mitochondrial targeting sequence of Su9 upstream to mCherry. Artificial ChiMERA tether^9^ was modified for the study by replacing GFP with Myc, and cloned into pM methionine centromeric plasmid. For a list of plasmids used in this study see Supplementary Table 2.

Yeast strains used in this study are based on the BY4741 background as listed in Supplementary Table 3. Yeast cells were grown in synthetic complete or drop-out media containing 2% dextrose at 30 °C.

All constructs were integrated into wild-type BY4741 genome by LiAc method and selected on corresponding synthetic drop-out agar plates, correct integration was confirmed by colony PCR. MSP1, TOM70, MDM10, MDM34, or MMM1 deletion mutants were created in yeast cells expressing yER-FlucDM-GFP_11_ and yMTS-mCherry-GFP_1–10_ by replacing the corresponding gene with a leucine cassette, and were grown on plates without Leucine. The Leucine cassette was PCR amplified with homologous overhangs corresponding to the UTR of gene to be deleted for targeted gene deletion. The deletions were confirmed by colony PCR, followed by sequencing. ΔMDM10 strains were complemented with Myc-tagged MDM10 point mutants or artificial ChiMERA tether cloned into methionine centromeric plasmid (pM) and selected on plates without methionine. The point mutants used were MDM10 Y73, 75 A mutant which shows impaired SAM50 function and MDM10 Y296, F298A mutant which exhibits impaired ERMES function^20^. The expression levels of yER-FlucDM-GFP_11_, yMTS-mCherry-GFP_1–10_ and complementing mutant MDM10 or ChiMERA tether were corroborated using Western blot.

### Yeast microscopy

Live-cell images of yeast were acquired using Leica SP8 confocal microscope with white laser line (WLL) and 63x oil-immersion NA = 1.4 objective. GFP and mCherry were excited at 488 nm and 581 nm respectively. The emission was collected onto HyD detectors in photon counting mode. The emission filter for GFP was set to 500–550 nm and for mCherry to 591–700 nm. Multi-frame alternating excitation configuration was used to avoid GFP spillover into mCherry channel. Processing of images was done using ImageJ (NIH) software. For signal quantification, we used embedded ImageJ function Analyze Particles after we segmented our images based on mCherry-mitochondrial signal. GFP signal was calculated only within mitochondrial areas to avoid interference with possible nonspecific cytoplasmic signals. Median fluorescent signal was then averaged in between technical and biological repeats. For quantification of cells exhibiting co-localization, we manually counted the cells where both markers were expressed and collocate when we overlay the GFP and mCherry channels. The ratio of the cells displaying the co-localization divided by the total number of cells was averaged between biological repeats.

### Statistics and reproducibility

The number of independent biological replicates (transfection events) used in every experiment is specified in the corresponding figure legend.

Statistical analysis was performed with Prism 5.0 or Excel software. For all assays, except quantification of wide-field fluorescent microscopy, unpaired Student’s t-test was used to determine significant difference between analyzed samples. The significance of wide-field fluorescent microscopy quantification was calculated using Mann–Whitney test.

### Reporting Summary

Further information on research design is available in the Nature Research Reporting Summary linked to this article.

## Supplementary information

Supplementary Material

Description of Additional Supplementary Files

Supplementary Data

Reporting Summary

## Data Availability

Plasmids generated in this study have been submitted to Addgene. Corresponding ID numbers are present in the Supplementary Tables 1–3. Data used for generating the charts are provided in Supplementary Data.
