# Supplementary material for: ER-misfolded proteins become sequestered with mitochondria and impair mitochondrial function
Source: Commun Biol. 2021 Dec 2;4:1350. doi: 10.1038/s42003-021-02873-w (PMC8640021; doi:10.1038/s42003-021-02873-w)
Supplement: Supplementary file 1 — Supplementary Material [file 42003_2021_2873_MOESM1_ESM.pdf]

	Cyto fluc	ER fluc
Fluc activity in each fraction	Total	Total
Exp1	512072.2	14135.6
Exp2	594397.2	11556.6
Exp3	553234.7	12846.1

	Cyto fluc	ER fluc
WB Fluc signal	Total	Total
Exp1	3172140	2916756
Exp2	2075535	1587762
Exp3	2556398	3835000

	Cyto fluc	ER fluc
Fluc activity/WB signal * 100	Total	Total
Exp1	16.14	0.48
Exp2	28.64	0.73
Exp3	21.64	0.33
Average	22	0.5
	p = 0.004	

Normalized to cyto-Fluc

	Cyto fluc	ER fluc
	Total	Total
	100	3.00
	100	2.54
	100	1.55
Average	100	2.4

Supplementary Figure 1. Semi-quantitative estimation of relative F-luc activity in total extracts of HEK293 cells transfected with Cyto-Fluc and ER-Fluc vectors. Enzymatic F-luc activity and densitometry data of 3 independent experiments presented in Fig. 1d. For a representative Western blot, see Fig. 1a.

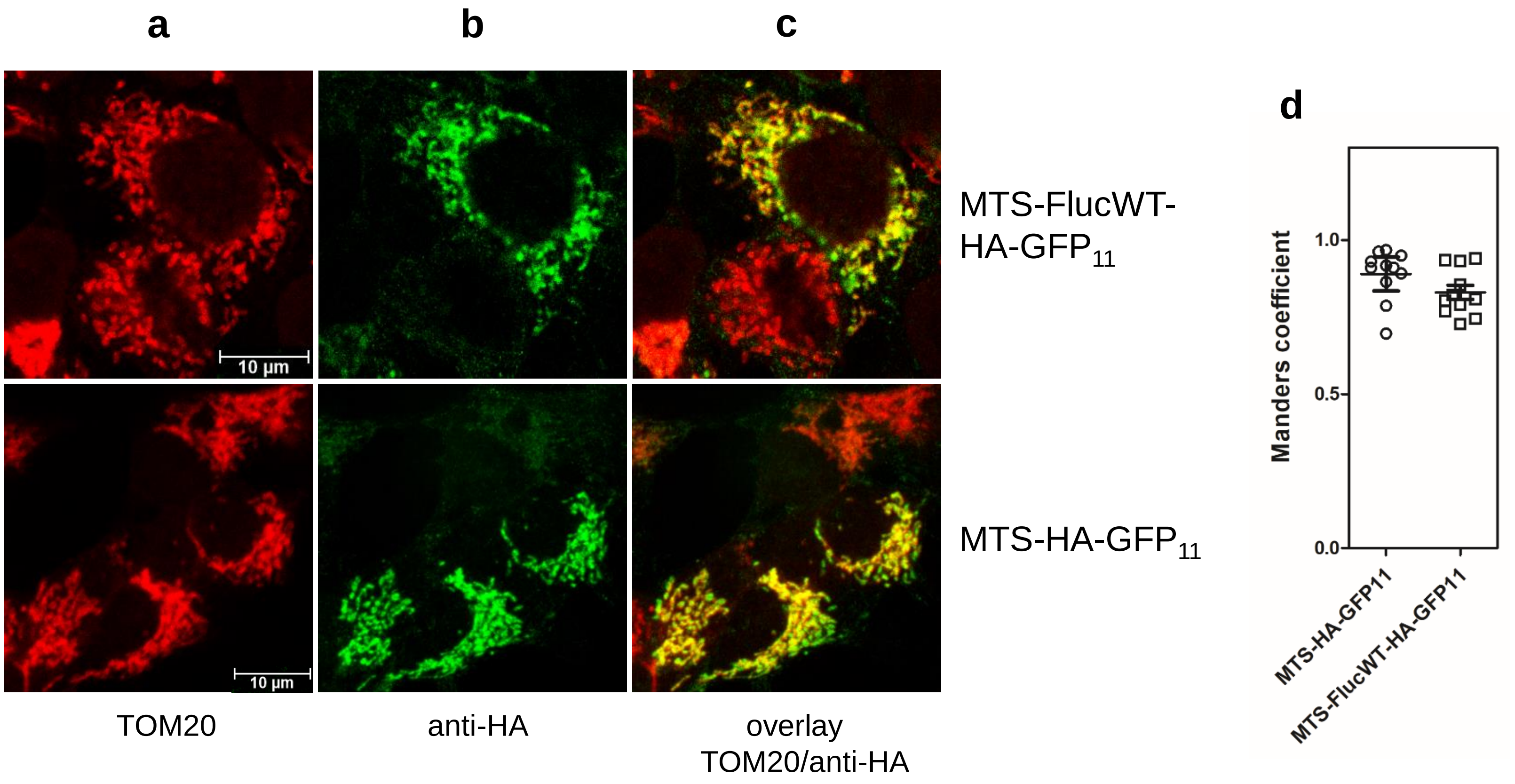

Supplementary Figure 2. Mitochondrial localization of MTS-FlucWT-HA-GFP$_{11}$ and MTS-HA-GFP$_{11}$ visualized by fluorescent confocal microscopy. Representative microscopy image showing co-localization of MTS-FlucWT-HA-GFP$_{11}$ or MTS-HA-GFP$_{11}$ with mitochondrial TOM20. a) – anti-TOM20 antibody, red; b) – anti-HA antibody, green; c) – overlay a) and b), co-localized signal – yellow, d) Manders correlation coefficient M1 for colocalization analysis of anti-TOM20 and anti-HA fluorescence signals. Scale bar = 10µm.

Gal-Trans

		Total	Mito
Exp.1	**Myc-tag**	18080084	10931461
	**COXI**	415008	7982339
Exp.2	**Myc-tag**	5219130	5022240
	**COXI**	356985	6018499
Exp.3	**Myc-tag**	3074700	2773300
	**COXI**	253952	2885592

ER-Fluc

		Total	Mito
Exp.1	**Fluc**	1963878	5424332
	**COXI**	331452	7050692
Exp.2	**Fluc**	1882636	6949980
	**COXI**	78876	985650
Exp.3	**Fluc**	3171616	6774066
	**COXI**	342474	3128192

MTS-Fluc

		Total	Mito
Exp.1	**Fluc**	699754	2043810
	**COXI**	599616	2708712
Exp.2	**Fluc**	3249922	8121216
	**COXI**	526620	2434792
Exp.3	**Fluc**	4221854	6758832
	**COXI**	1116780	3068226

	Gal-Trans	ER-Fluc	MTS-Fluc
Exp.1	**0.03**	**0.13**	**0.65**
Exp.2	**0.06**	**0.30**	**0.54**
Exp.3	**0.08**	**0.23**	**0.58**

p = 0.03

Supplementary Figure 3. Semi-quantitative estimation of reporter protein localized in the mitochondrial fraction.
Densitometry data of 3 independent experiments for Gal-Trans, ER-Fluc, and MTS-Fluc. For a representative Western blot, see Fig. 1e, for densitometry analysis see Fig. 1f. Reporter proteins (ER-Fluc, MTS-Fluc, Gal-Trans) were normalized to COXI and the recovery in the mitochondrial fraction relative to total protein quantified using the following formula: $\frac{\text{Reporter protein mitochondria}}{\text{COXI mitochondria}} : \frac{\text{Reporter protein total}}{\text{COXI total}}$.

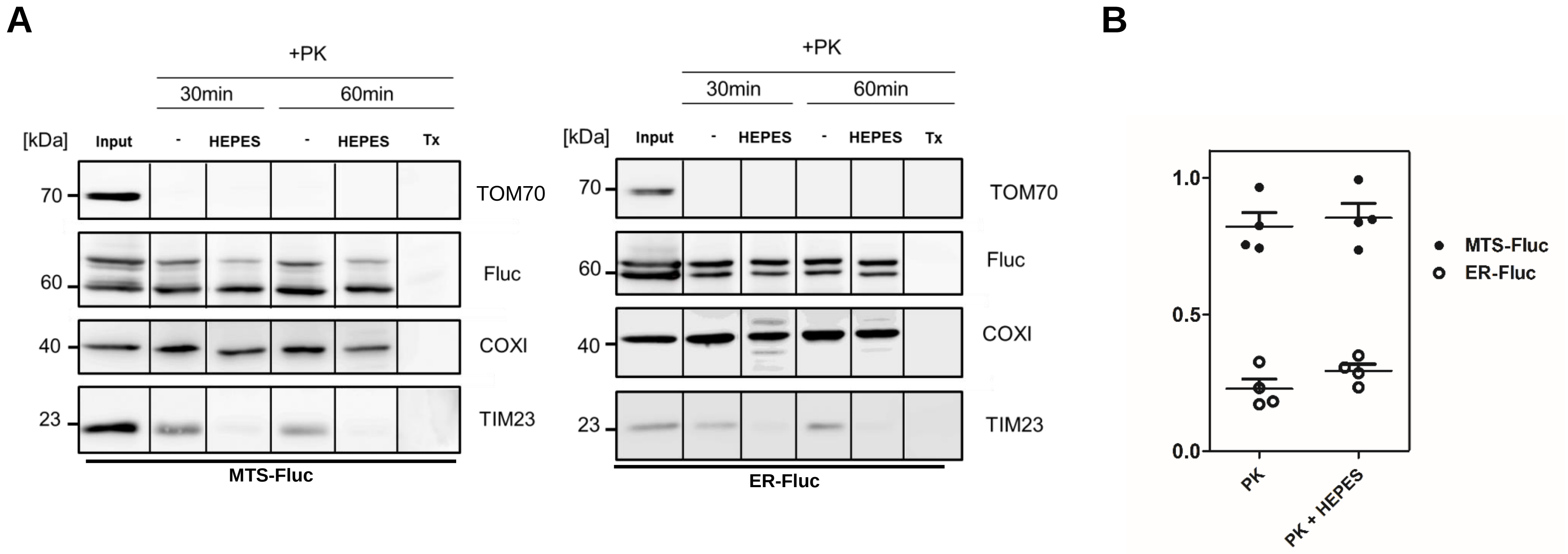

Supplementary Figure 4. A) Representative Western blot of mitochondrial fractions isolated from HEK293 cells expressing ER-Fluc and mitochondria targeted MTS-Fluc. Fractions were treated with proteinase K for indicated time with or without prior treatment with 20 mM HEPES or 2% Triton X-100 (Tx). Left line: input control. Following marker proteins were used: TOM70 (outer membrane), TIM23 (inner membrane), COX1 (matrix). **B)** Quantification of Fluc protected from PK following normalization to COX1, samples were treated with proteinase K for 30 min with or without hypotonic shock by 20 mM HEPES. MTS-Fluc ●, ER-Fluc **O** (N =4, number of independent experiments). See Supplementary Fig. 18A, B for uncropped gel scans.

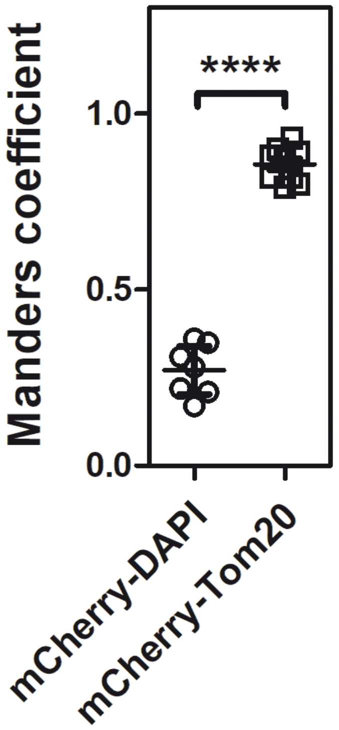

Supplementary Figure 5. Manders correlation coefficient M1 for colocalization analysis of MTS-mCherry-GFP_{1-10}/MTS-GFP_{11} and TOM20 fluorescence signals. Nuclear-localized DAPI fluorescence was used as a negative control for MTS-mCherry colocalization analysis. ****$p<0.0001$.

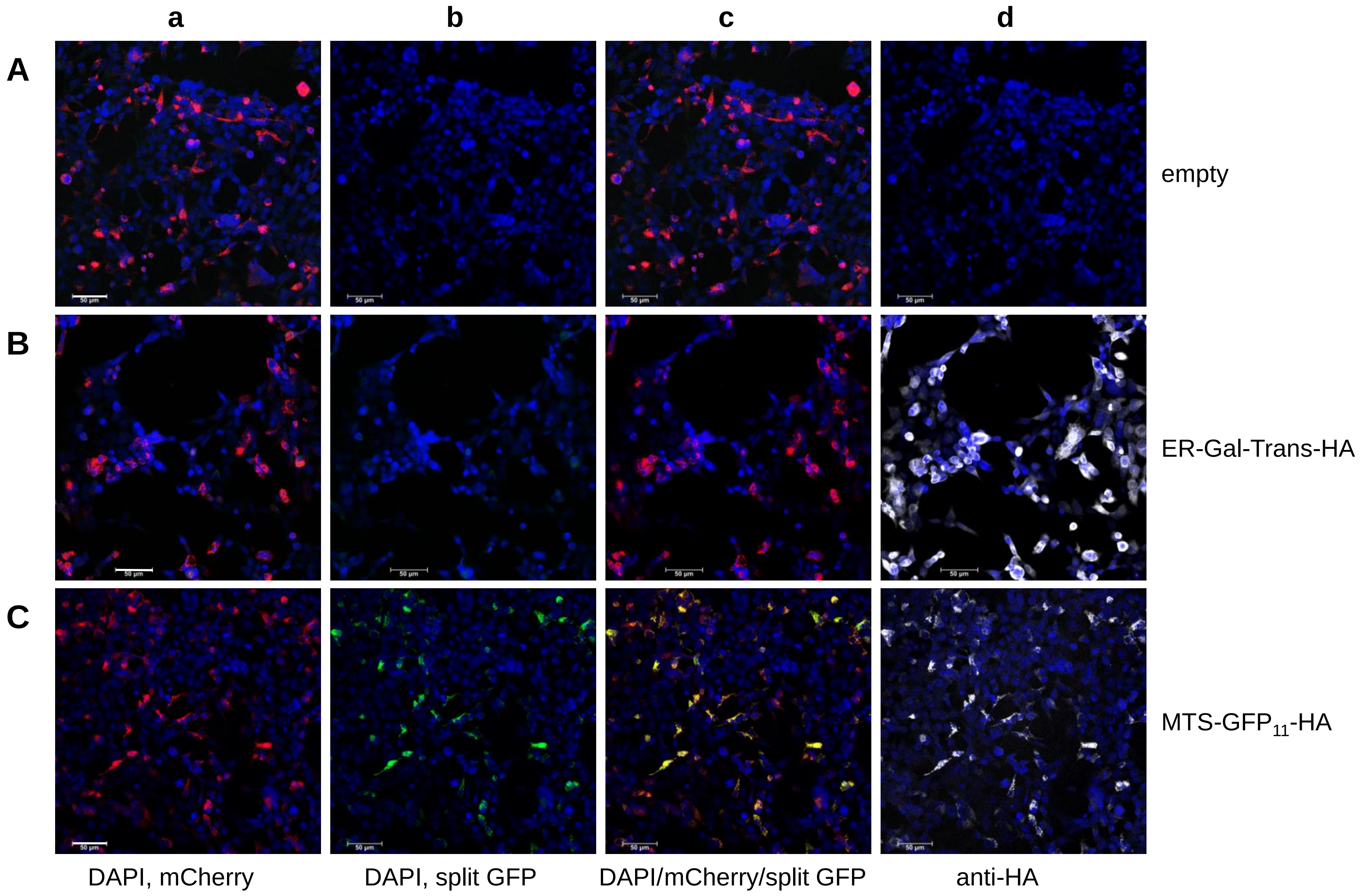
a
b
c
d
A
B
C
empty
ER-Gal-Trans-HA
MTS-GFP11-HA
50 µm
DAPI, mCherry
DAPI, split GFP
DAPI/mCherry/split GFP
anti-HA

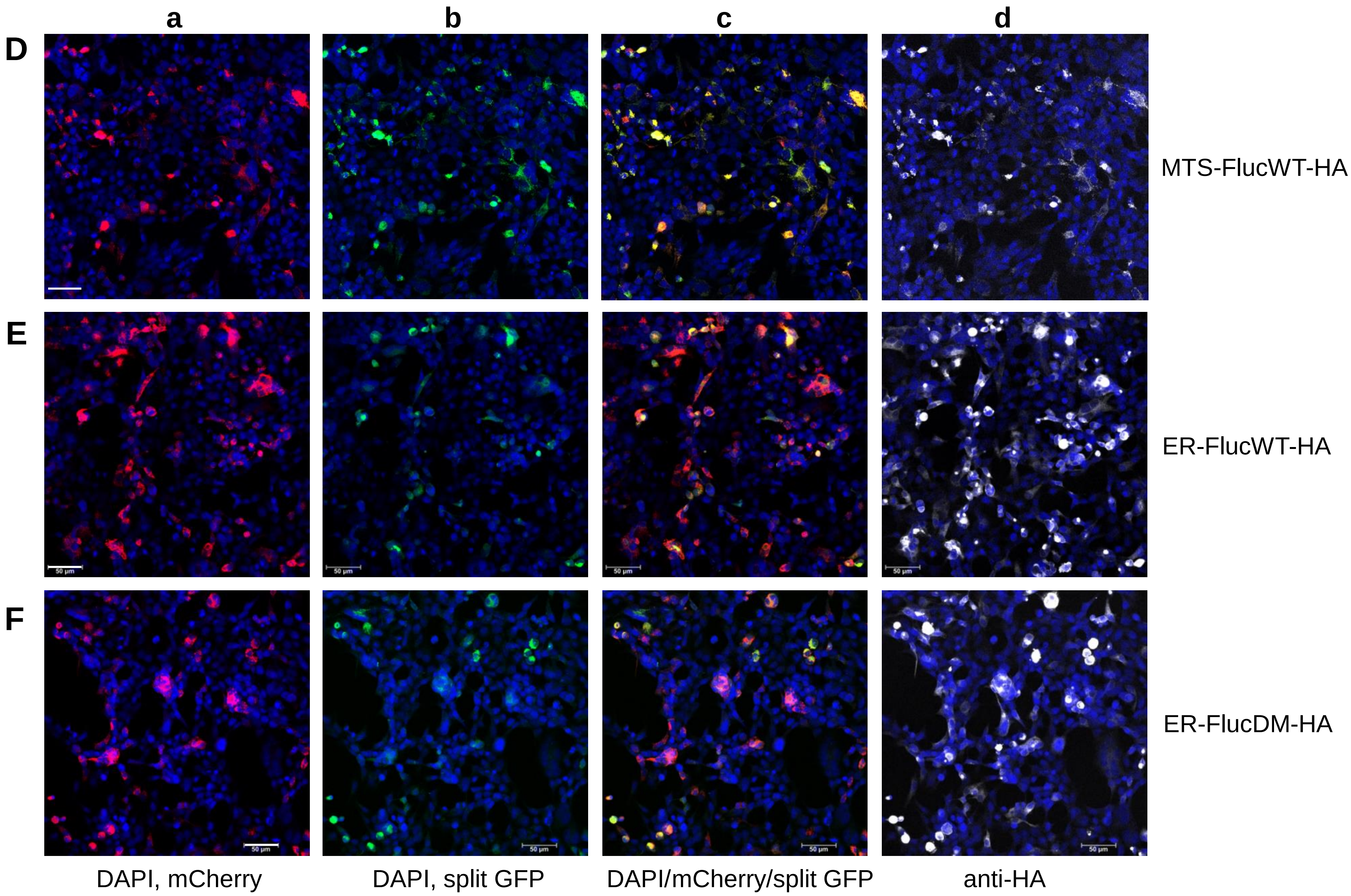

Supplementary Figure 6. Association of ER-FlucWT and ER-FlucDM with mitochondria, representative low magnification images. A) Cells transfected with MTS-mCherry-$GFP_{1\text{-}10}$ only, **B)** cells co-transfected with MTS-mCherry-$GFP_{1\text{-}10}$ / ER-Gal-Trans-HA-GFP_{11}, **C)** cells co-transfected with MTS-mCherry-$GFP_{1\text{-}10}$ / MTS-HA-GFP_{11}, **D)** cells co-transfected with MTS-mCherry-$GFP_{1\text{-}10}$ / MTS-FlucWT-HA-GFP_{11}, **E)** cells co-transfected with MTS-mCherry-$GFP_{1\text{-}10}$ / ER-FlucWT-HA-GFP_{11}, **F)** cells co-transfected with MTS-mCherry-$GFP_{1\text{-}10}$ / ER-FlucDM-HA-GFP_{11}. Overlay with DAPI, blue: **a** – MTS-mCherry, red; **b** – split-GFP, green; **c** – overlay a) and b), colocalized yellow; **d** – anti-HA-tag, white. Scale bar = 50µm.

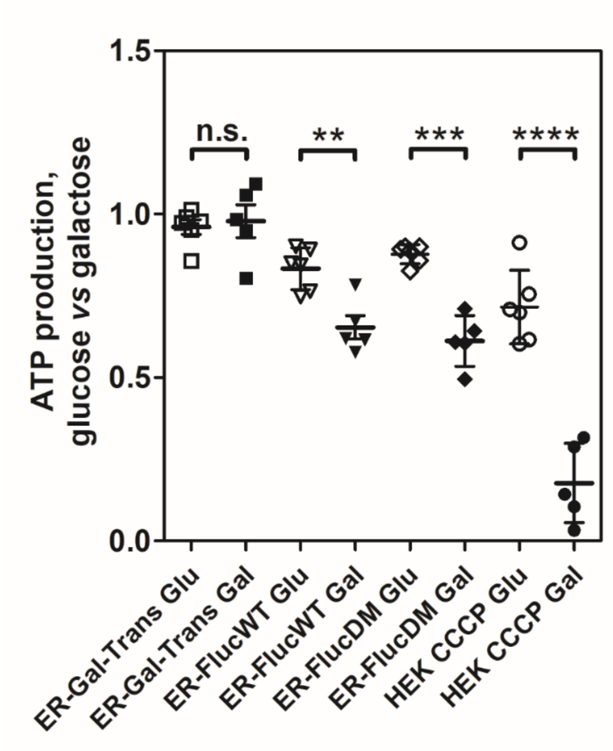

Supplementary Figure 7. ATP production in cells with normal glycolysis compared to ATP production in cells with suppressed glycolysis (mean fluorescence ± SEM; N=5). Cells were incubated in DMEM supplemented with glucose (Glu), 4.5g/L and FBS, 10%; or in DMEM supplemented with galactose (Gal), 2.5g/L and no FBS. **p<0.01, ***p<0.001, ****p<0.0001. N - number of independent transient transfections; for each transfection 2 technical replicates were done.

AAT WT

Exp.1

	Total	Mito
**Myc Tag**	247592	83329
**OXPHOS**	37161	381081

Exp.2

	Total	Mito
**Myc Tag**	3822254	1339308
**OXPHOS**	937412	4373584

Exp.3

	Total	Mito
**Myc Tag**	1519800	799876
**OXPHOS**	391680	2927484

ATZ

Exp.1

	Total	Mito
**Myc Tag**	954843	2357478
**OXPHOS**	39454	215611

Exp.2

	Total	Mito
**Myc Tag**	29638508	46451399
**OXPHOS**	826556	2996138

Exp.3

	Total	Mito
**Myc Tag**	2181648	5878010
**OXPHOS**	94416	562629

	AAT WT	ATZ
Exp.1	0.03	0.45
Exp.2	0.08	0.43
Exp.3	0.07	0.45

$p = 1.3 \times 10^{-5}$

Supplementary Figure 8. Semi-quantitative estimation of WT-AT and ATZ protein localized in the mitochondrial fraction. Densitometry data of 3 independent experiments. For a representative Western blot, see Fig. 4c, for densitometry analysis see Fig. 4d. Reporter proteins (WT-AT, ATZ) were normalized to COXI and the recovery in the mitochondrial fraction relative to total protein quantified using the following formula: $\frac{\text{Reporter protein mitochondria}}{\text{COXI mitochondria}} : \frac{\text{Reporter protein total}}{\text{COXI total}}$.

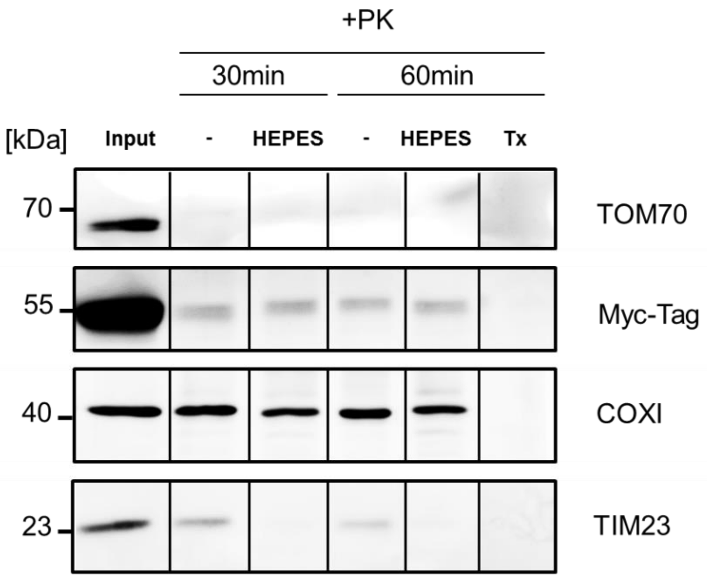

Supplementary Figure 9. Representative Western blot of mitochondrial fractions isolated from HEK293 cells expressing myc-tagged ATZ. Input control: left. Fractions were treated with proteinase K for the indicated times with or without prior treatment with 20 mM HEPES or 2% Triton X-100. Following marker proteins were used: TOM70 (outer membrane), TIM23 (inner membrane), COX1 (matrix). See Supplementary Fig. 19 for uncropped gel scans.

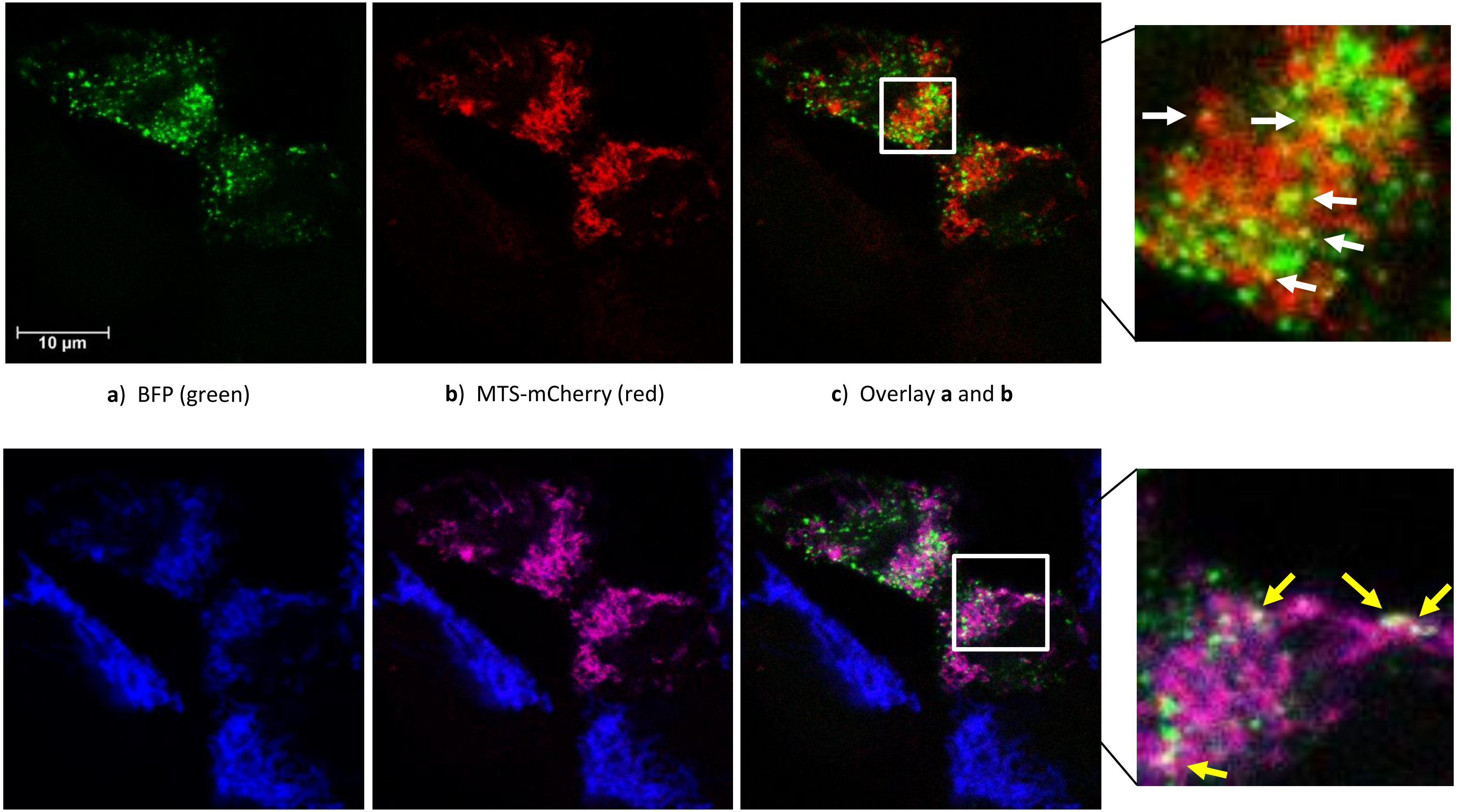

a) BFP (green) b) MTS-mCherry (red) c) Overlay **a** and **b**

d) TOM20 antibody (blue) e) Overlay **b** and **d** f) Overlay **a**, **b** and **d**

Supplementary Figure 10A. **Association of ATZ with mitochondria assessed by confocal microscopy**. HEK293 cells were co-transfected with pATZ-BFP and pMTS-mCherry (as a mitochondrial marker). Colocalization of ATZ-BFP (green) and MTS-mCherry (red) is shown in yellow and marked with white arrows. Colocalization of ATZ-BFP (green), MTS-mCherry (red) and mito-TOM20 (blue) is shown in white and marked with yellow arrows.

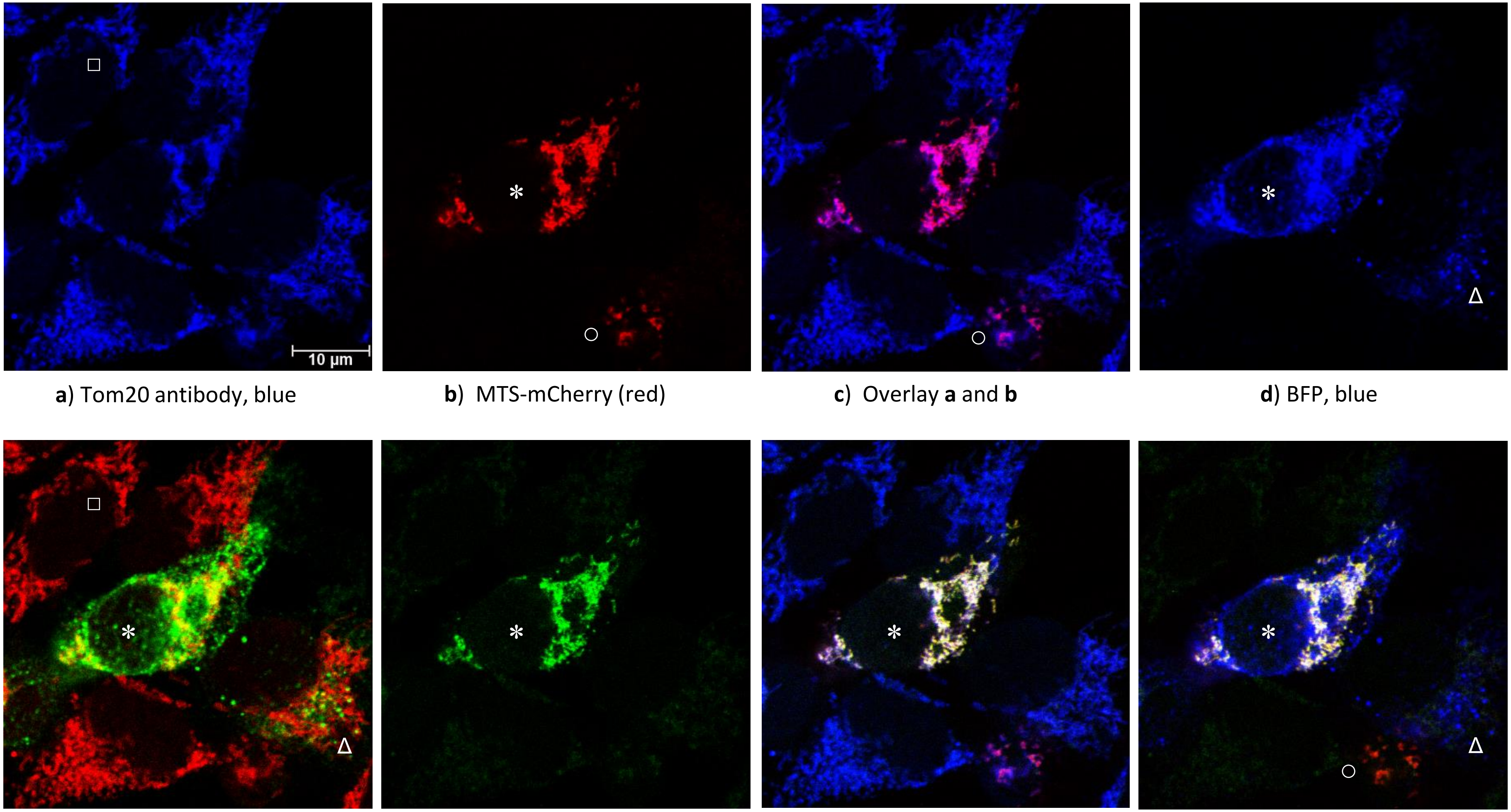

a) Tom20 antibody, blue

b) MTS-mCherry (red)

c) Overlay **a** and **b**

d) BFP, blue

e) Overlay **Tom20** (red) and **BFP** (green)

f) Split-GFP (green)

g) Overlay **a**, **b** and **f**

h) Overlay **b**, **d** and **f**

Supplementary Figure 10B. **Association of ATZ with mitochondria assessed by confocal microscopy**. HEK293 cells were co-transfected with pATZ-BFP-HA-GFP_{11} and pMTS-mCherry-$GFP_{1\text{-}10}$. **a** – anti-TOM20 antibodies, blue; **b** – mCherry, red; **c** – overlay of a and b; **d** – BFP, blue; **e** – overlay of Tom20 (red) and BFP (green); **f** – split-GFP, green ; **g** – overlay of a, b, and f (overlay of TOM20, mCherry, and split-GFP will result in a white signal); **h** – overlay of b, d, and f (overlay of mCherry, BFP, and split-GFP will result in a white signal).

□ - non-transfected WT cell; Δ- cell transfected with pATZ-BFP-HA-GFP_{11} only; ○ - cell transfected with pMTS-mCherry-$GFP_{1\text{-}10}$ only;

∗ - cell transfected with pATZ-BFP-HA-GFP_{11} and pMTS-mCherry-$GFP_{1\text{-}10}$.

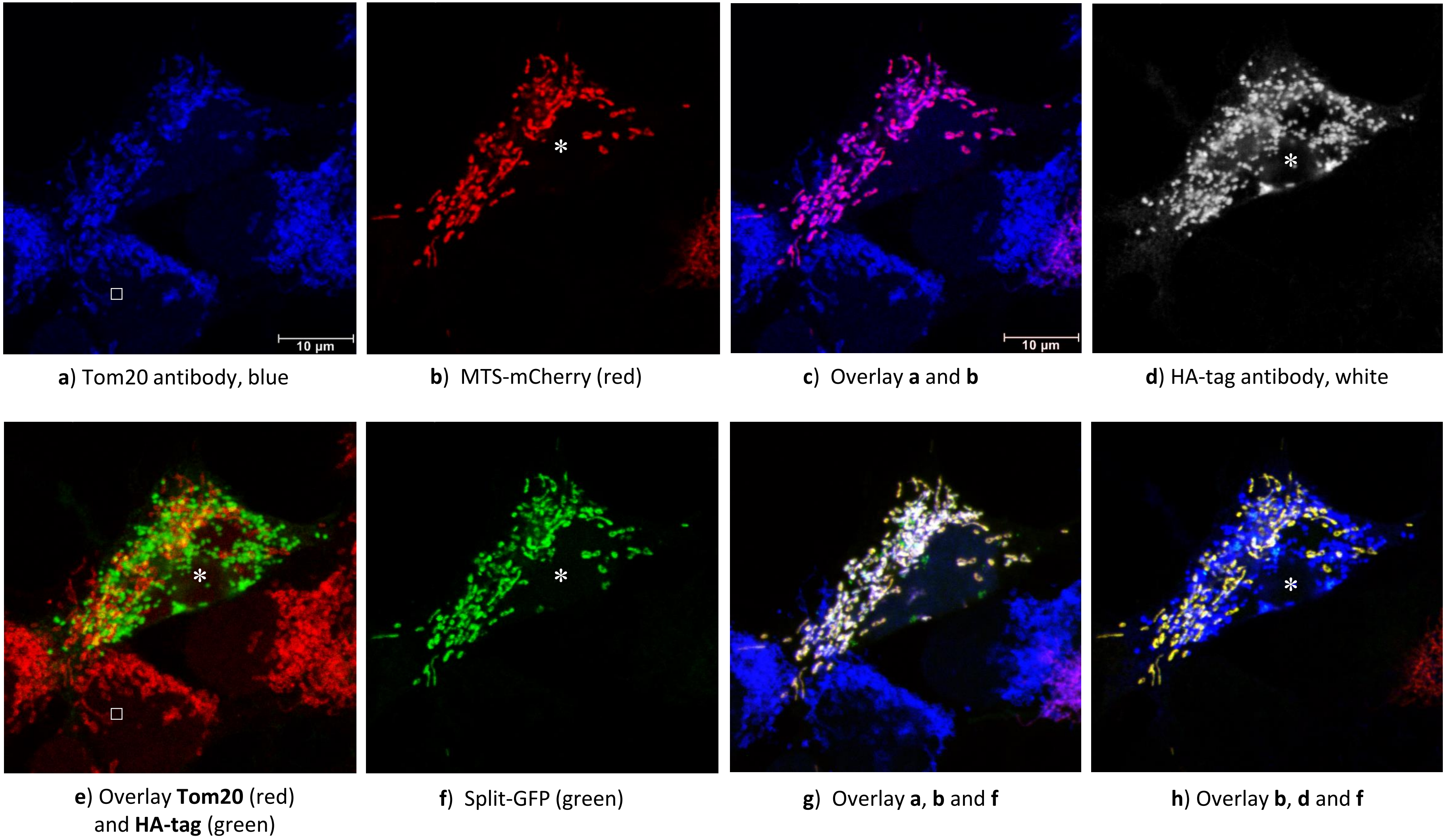

Supplementary Figure 10C. **Association of ATZ with mitochondria assessed by confocal microscopy**. HEK293 cells were co-transfected with pATZ-BFP-HA-GFP_{11} and pMTS-mCherry-$GFP_{1\text{-}10}$. **a** – anti-TOM20 antibodies, blue; **b** – mCherry, red; **c** – overlay of a and b; **d** – anti-HA-tag antibodies, white; **e** – overlay of Tom20 (red) and HA-tag (green); **f** – split-GFP, green ; **g** – overlay of a, b, and f (overlay of TOM20, mCherry, and split-GFP will result in a white signal); **h** – overlay of b, d (in blue), and f (overlay of mCherry, anti-HA-tag, and split-GFP will result in a white signal). □ - non-transfected WT cell; ∗ - cell transfected with pATZ-BFP-HA-GFP_{11} and pMTS-mCherry-$GFP_{1\text{-}10}$.

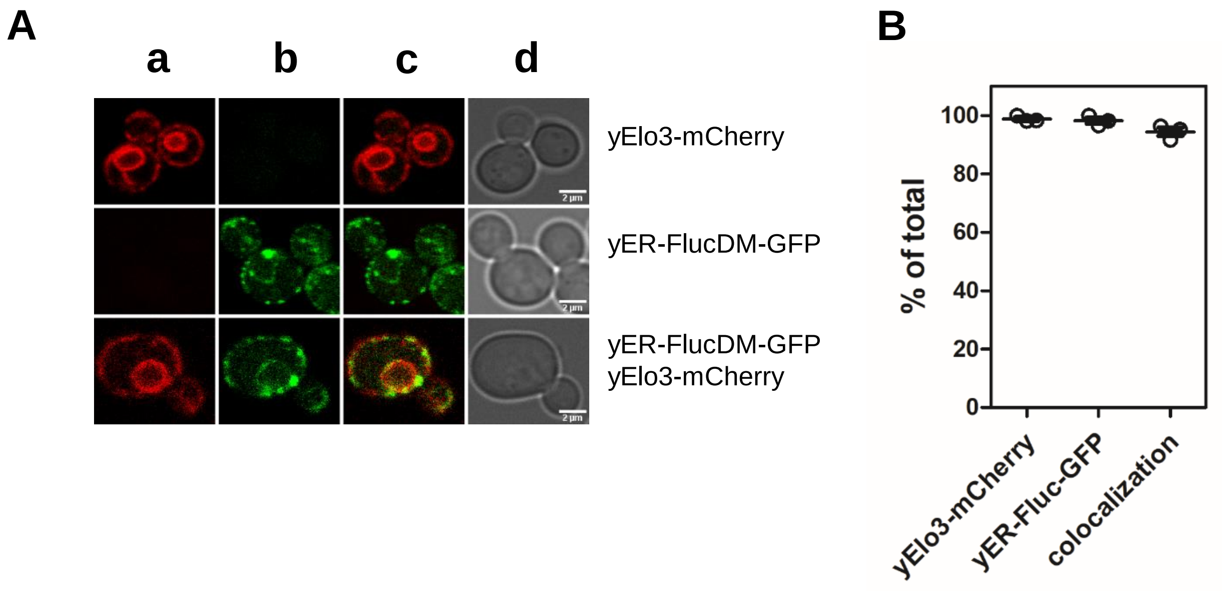

Supplementary Figure 11. A) Representative confocal images showing colocalization of yER-FlucDM-GFP with yElo3-mCherry (ER marker). a – mCherry, red; b – GFP, green, c – overlay a) and b), colocalized signal is shown in yellow; d – bright field. Scale bar = 2µm. **B**) Quantification of percentage of cells transfected with both yER-Fluc-GFP and yElo3-mCherry showing expression of indicated marker and colocalization in ER. Median ± interquartile range. Total of 156 cells were counted from three independent experiments.

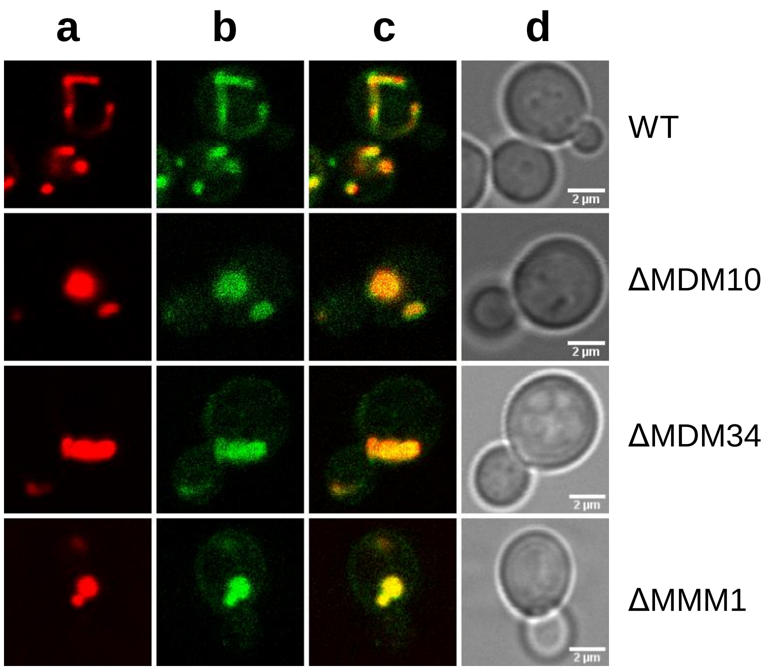

Supplementary Figure 12. Identification of mitochondria in yeast *Saccharomyces cerevisiae* WT, ΔMDM10, ΔMDM34, and ΔMMM1 deletion mutants using MitoTracker Green. Representative confocal images showing colocalization of MTS-mCherry with mitochondrial marker MitoTracker Green. **a** – yMTS-mCherry, red; **b** – MitoTracker, green; **c** – overlay a) and b), co-localized signal is shown in yellow, **d** – bright field. Scale bar = 2µm.

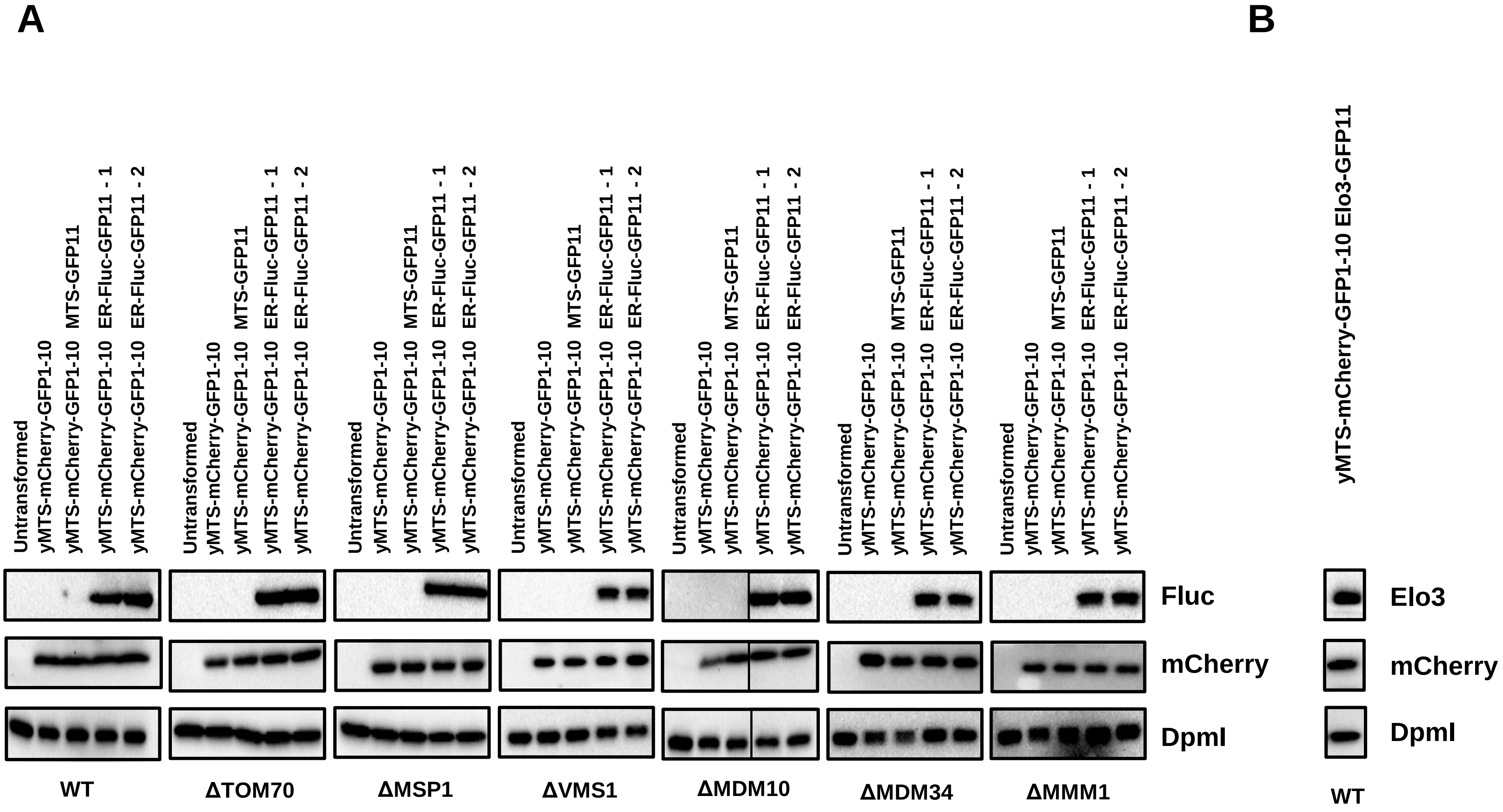

Supplementary Figure 13. **A**) Western blot demonstrating expression of yER-FlucDM-GFP$_{11}$ and yMTS-mCherry-GFP$_{1\text{-}10}$ in WT and deletion mutants. DpmI was used as loading control. **B**) Western blot demonstrating expression of yElo3-GFP$_{11}$ and yMTS-mCherry-GFP$_{1\text{-}10}$ in WT. DpmI was used as loading control. See Supplementary Fig. 20A-D for uncropped gel scans.

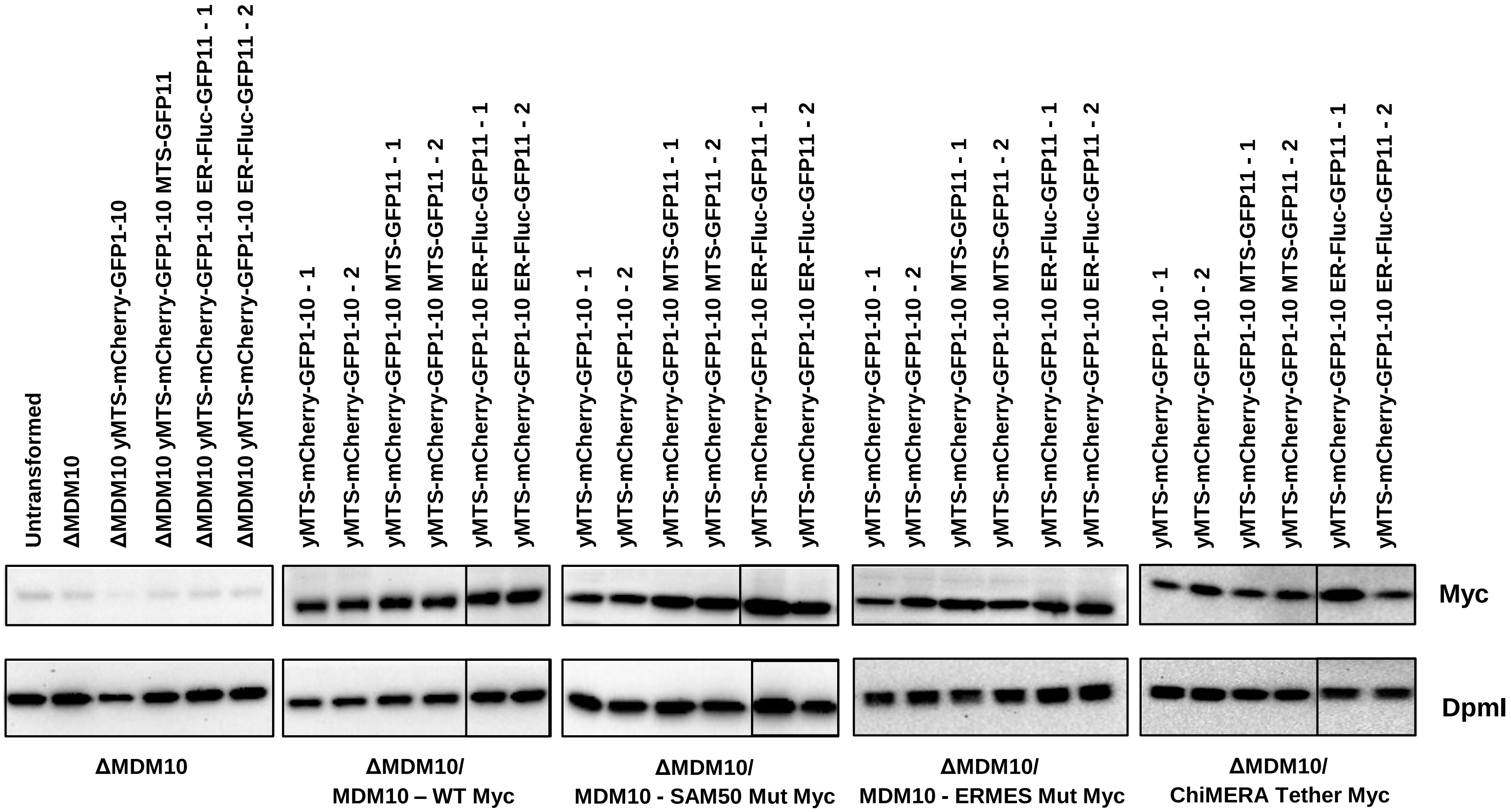

Supplementary Figure 14. Western blot demonstrating expression of complemented plasmids in MDM10 deletion mutant. Anti-myc antibody was used to detect the complementing myc-tagged proteins. DpmI was used as loading control. See Supplementary Fig. 21A, B for uncropped gel scans.

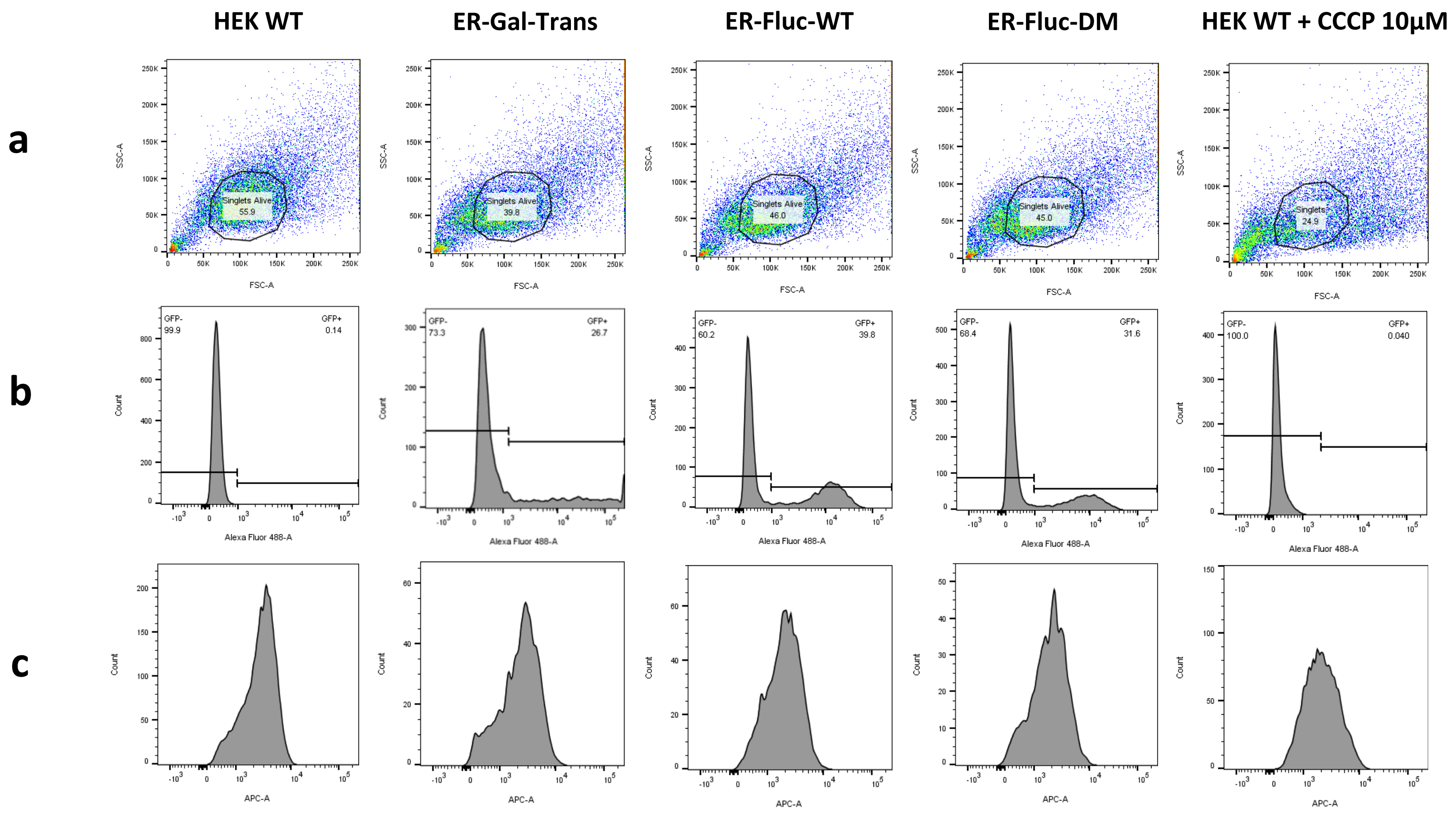

d

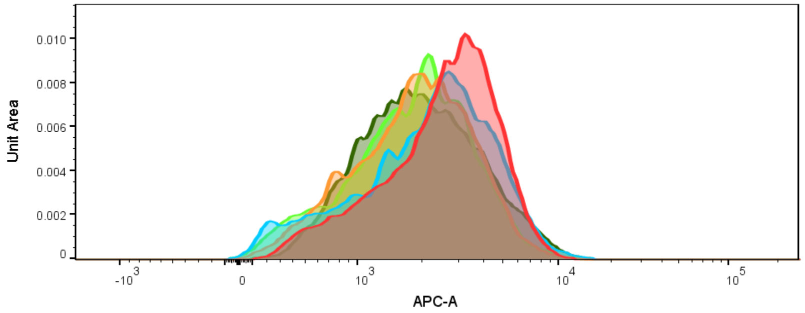

e

	Sample	Subpopulation	Mitotracker fluo, (APC-A, median)
red	HEK WT (-contr)	GFP-	2761
blue	ER-Gal-Trans	GFP+	2364
orange	ER-Fluc-WT	GFP+	1942
green	ER-Fluc-DM	GFP+	1939
dark green	HEK WT+CCCP 10μM (+contr)	GFP-	1957

Supplementary Figure 15A. FACS analysis. Gating strategy for HEK293 cells transfected with ER-Gal-Trans, ER-FlucWT or ER-FlucDM, fused with eGFP and treated with Mitotracker DR for determination of mitomass. a) Selected single cells. b) Selected GFP-negative control cells and GFP-positive transfected cells. c) Mitotracker DR fluorescence (channel APC). d) Overlay of Mitotracker peaks. e) Representative final data.

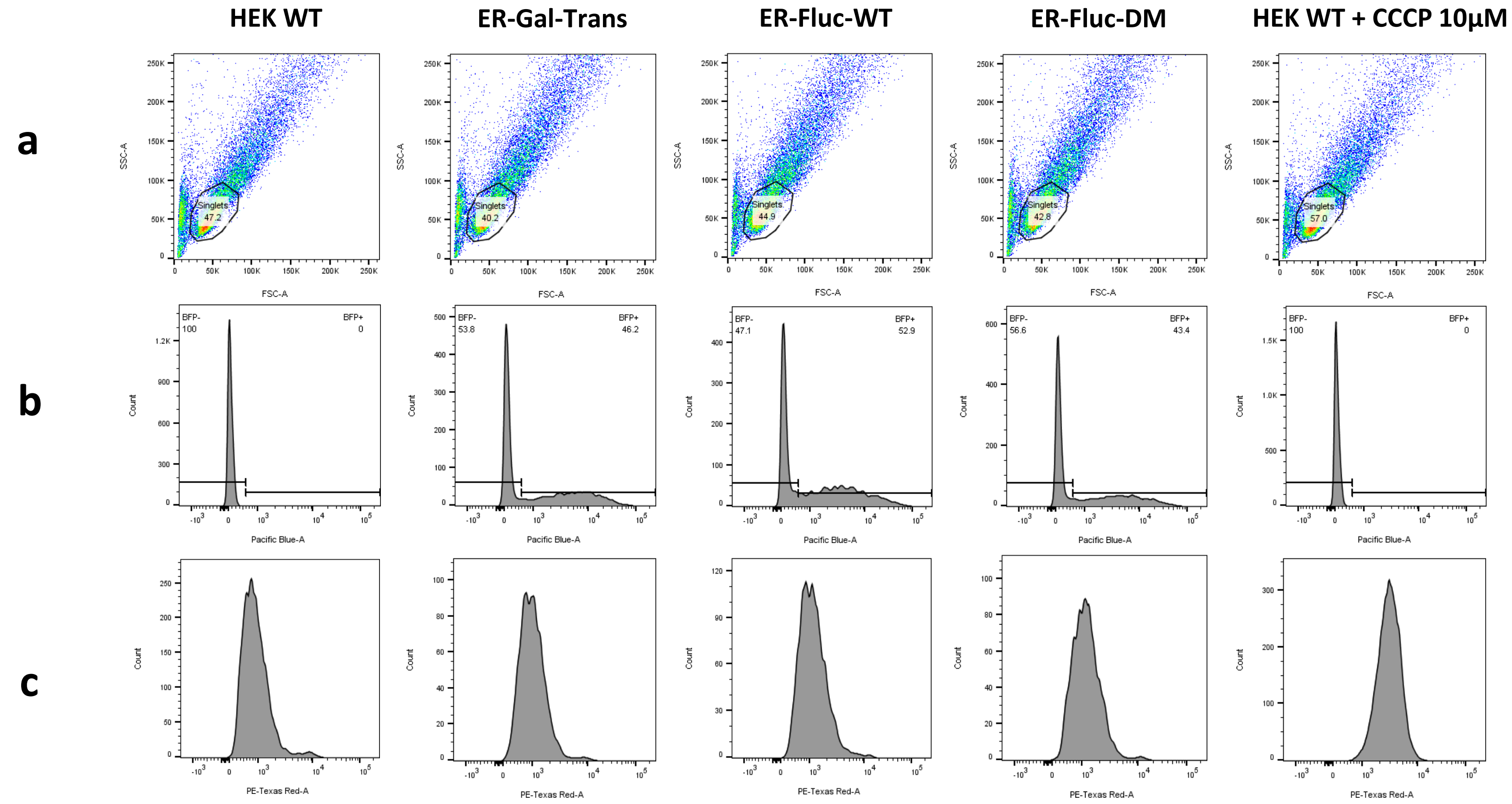

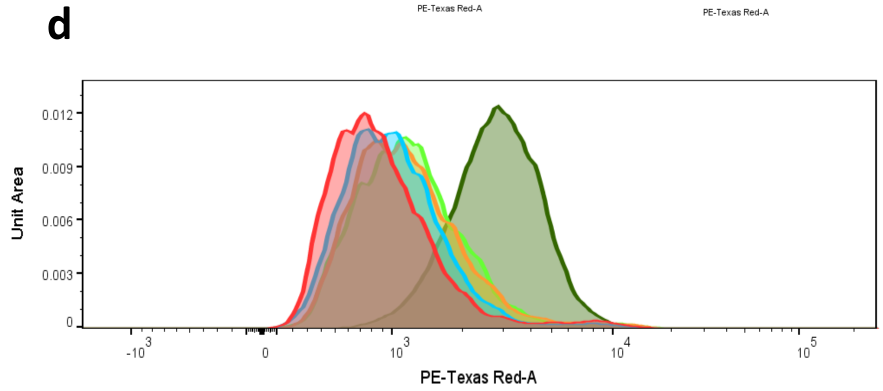

e

	Sample	Subpopulation	MitoSOX Red, (Texas Red-A, median)
	HEK WT (-contr)	BFP-	837
	ER-Gal-Trans	BFP+	980
	ER-Fluc-WT	BFP+	1101
	ER-Fluc-DM	BFP+	1161
	HEK WT+CCCP 10μM (+contr)	BFP-	2882

Supplementary Figure 15B. FACS analysis. Gating strategy for HEK293 cells transfected with ER-Gal-Trans, ER-FlucWT or ER-FlucDM, cotransfected with pBFP-Tag and treated with MitoSOX Red. a) Selected single cells. b) Selected BFP-negative control cells and BFP-positive transfected cells. c) MitoSOX fluorescence (channel Texas Red). d) Overlay of MitoSOX peaks. e) Representative final data.

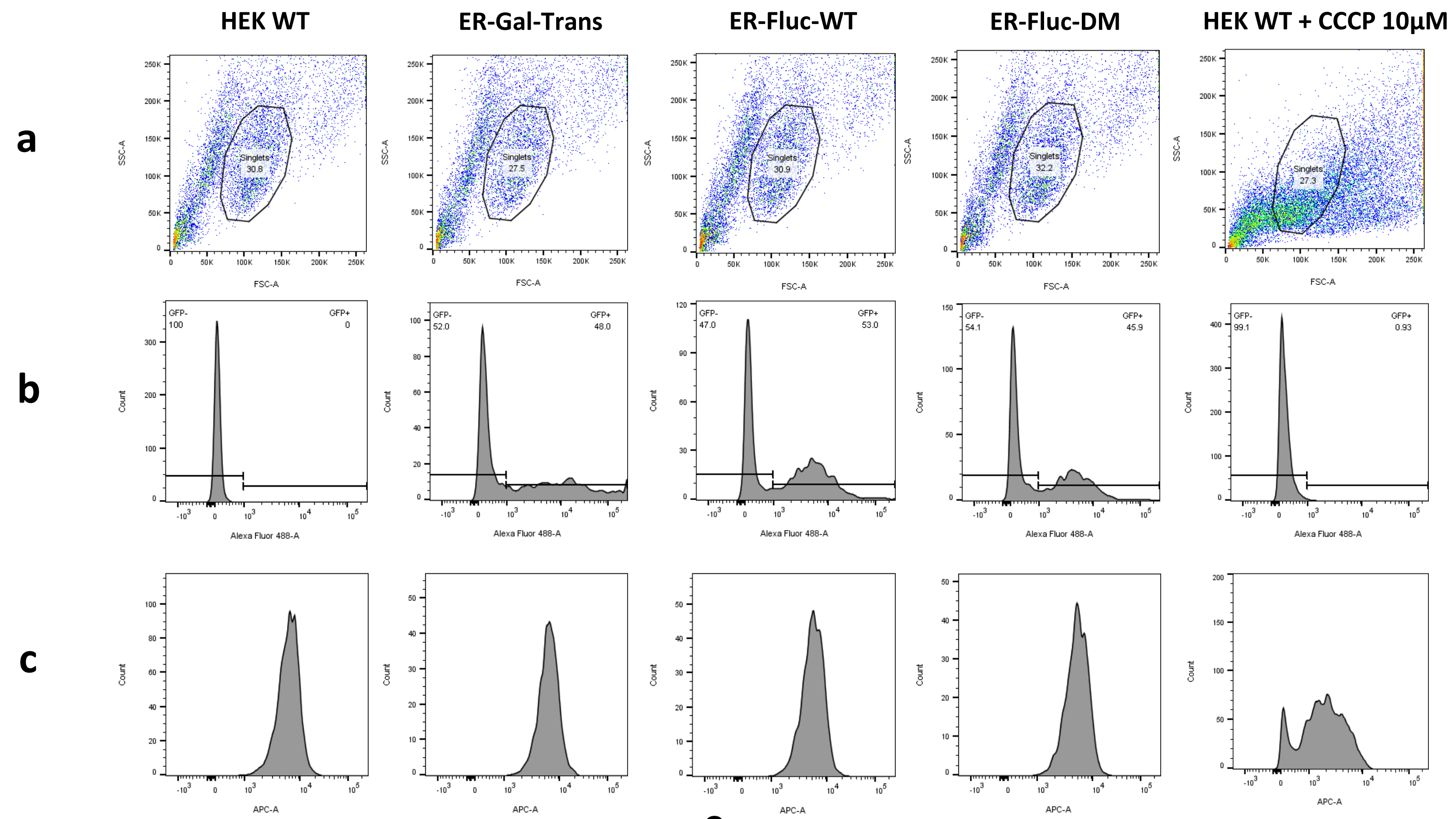

d

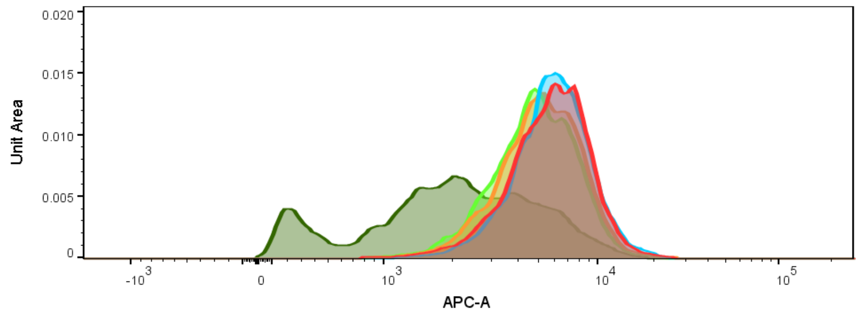

e

	Sample	Subpopulation	DilC1(5) fluo, (APC-A, median)
	HEK WT (-contr)	GFP-	6002
	ER-Gal-Trans	GFP+	6041
	ER-Fluc-WT	GFP+	5302
	ER-Fluc-DM	GFP+	4976
	HEK WT+CCCP 10μM (+contr)	GFP-	2136

Supplementary Figure 15C. FACS analysis. Gating strategy for HEK293 cells transfected with ER-Gal-Trans, ER-FlucWT or ER-FlucDM, fused with eGFP and treated with mito-membrane potential indicator DilC1(5). a) Selected single cells. b) Selected GFP-negative control cells and GFP-positive transfected cells. c) DilC1(5) fluorescence (channel APC). d) Overlay of DilC1(5) peaks. e) Representative final data.

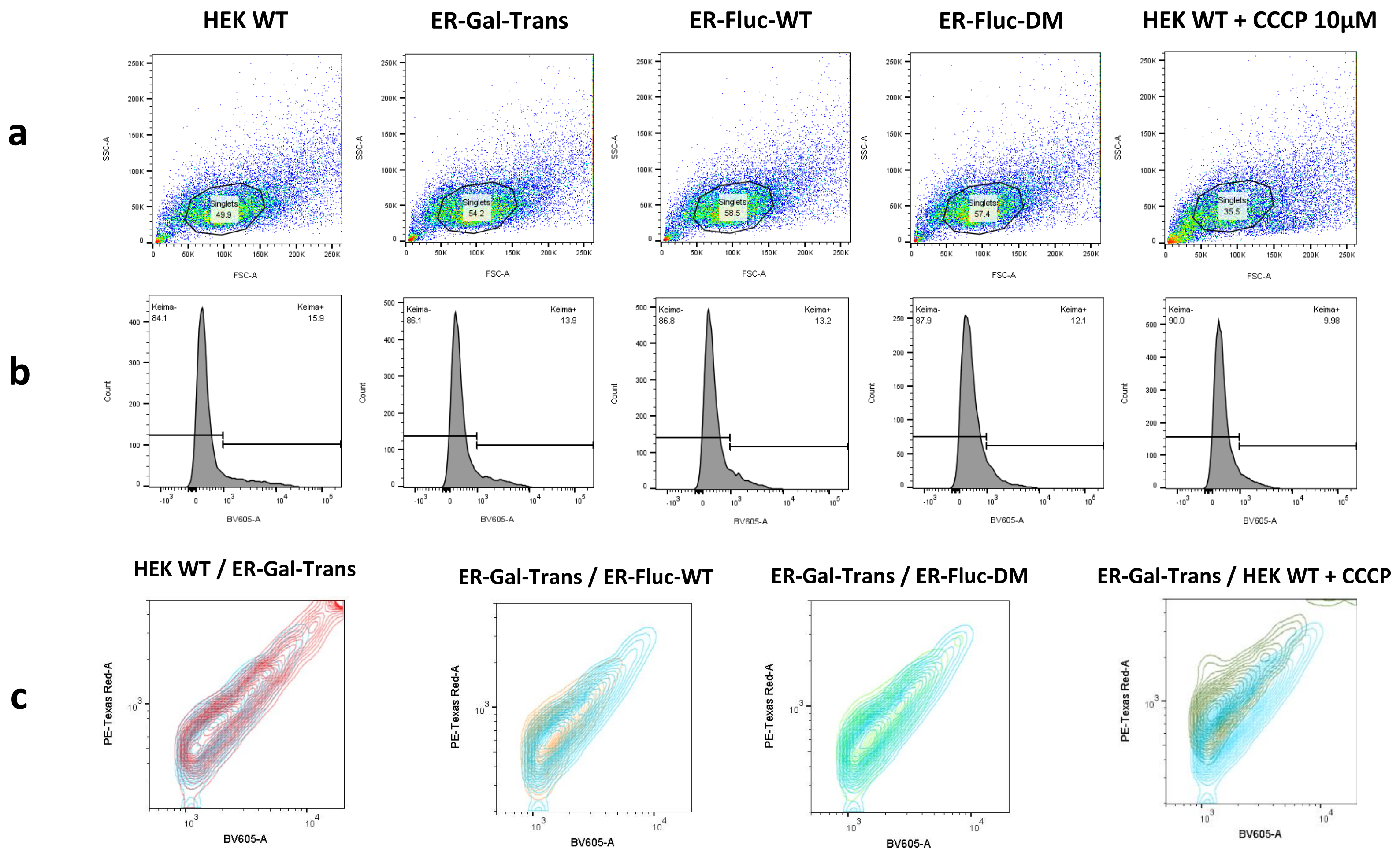

d

	Sample	Subpopulation	mKeima, neutral pH (BV605, median)	mKeima, low pH (Texas Red, median)	Mitophagy intensity coefficient (mKeima, ratio low pH / neutral pH)
(red)	HEK WT (-contr)	mKeima+	2608	949	0.363
(blue)	ER-Gal-Trans	mKeima+	1932	758	0.392
(orange)	ER-Fluc-WT	mKeima+	1479	695	0.469
(green)	ER-Fluc-DM	mKeima+	1579	733	0.464
(dark green)	HEK WT+CCCP 10µM (+contr)	mKeima+	1438	1017	0.707

Supplementary Figure 15D. FACS analysis. Gating strategy for HEK293 cells transfected with ER-Gal-Trans, ER-FlucWT or ER-FlucDM, cotransfected with mKeima Red. a) Selected single cells. b) Selected Keima-positive transfected cells. c) Overlay of mKeima fluorescence at neutral pH and low pH. e) Representative final data.

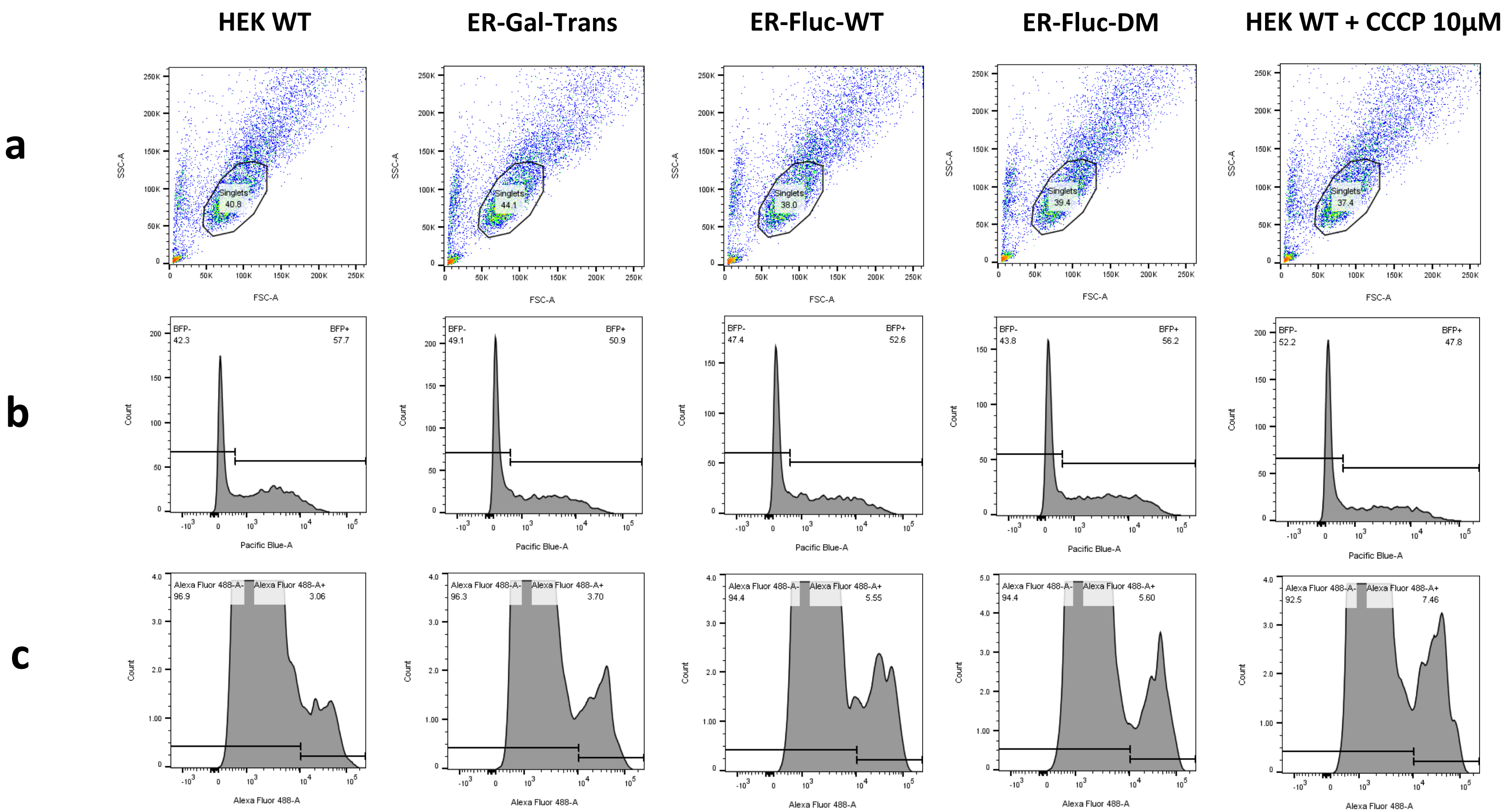

d

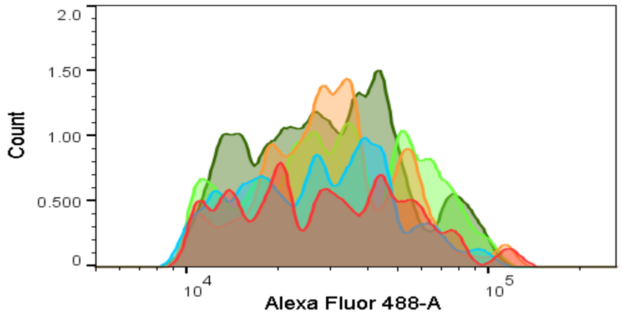

e

	Sample	Subpopulation	% of the Caspase 3/7 positive cells
	HEK WT (-contr)	Caspase positive	3.06
	ER-Gal-Trans	Caspase positive	3.70
	ER-Fluc-WT	Caspase positive	5.55
	ER-Fluc-DM	Caspase positive	5.80
	HEK WT+CCCP 10μM (+contr)	Caspase positive	7.46

Supplementary Figure 15E. **FACS analysis. Gating strategy for HEK293 cells transfected with ER-Gal-Trans, ER-FlucWT or ER-FlucDM, cotransfected with pBFP-Tag and treated with CellEvent Caspase-3/7 Green Assay**. a) Selected single cells. b) Selected BFP-positive transfected cells. c) CellEvent Caspase-3/7 Green fluorescence (channel AlexaFluor 488). d) Overlay of CellEvent Caspase-3/7 Green peaks. e) Representative final data showing Caspase 3/7 activated cells in %.

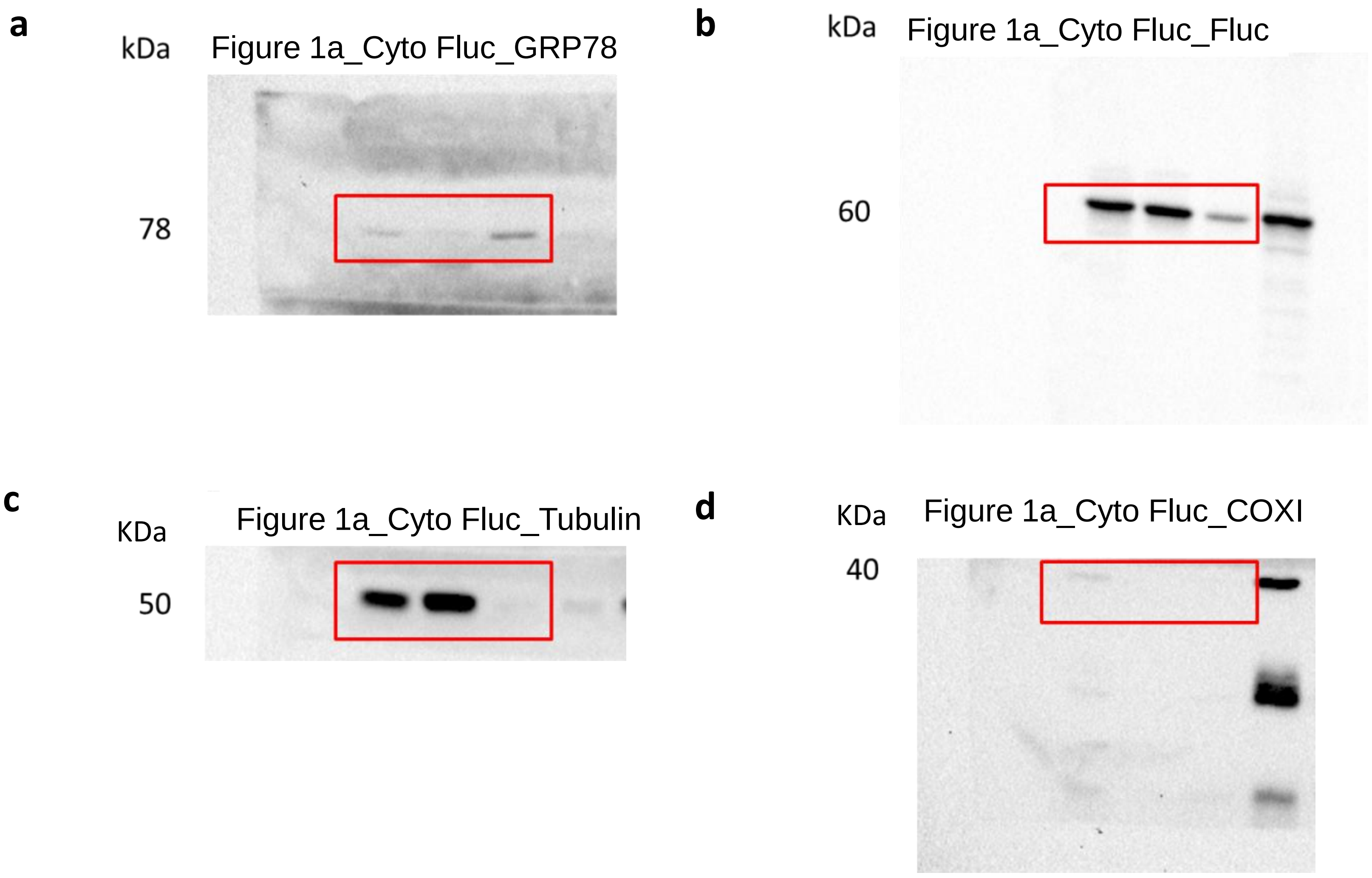

Supplementary Figure 16A. Uncropped Western blots used for Fig. 1a: HEK 293 cells transfected with cyto-Fluc. Red frames show the protein bands used in the figure. Due to similar protein size, sample replicates were run on two different gels, blotted, membranes cut and incubated with different antibodies: a) GRP78, b) Fluc, c) Tubulin, and d) COXI. GRP78, Tubulin and COXI were from the same gel.

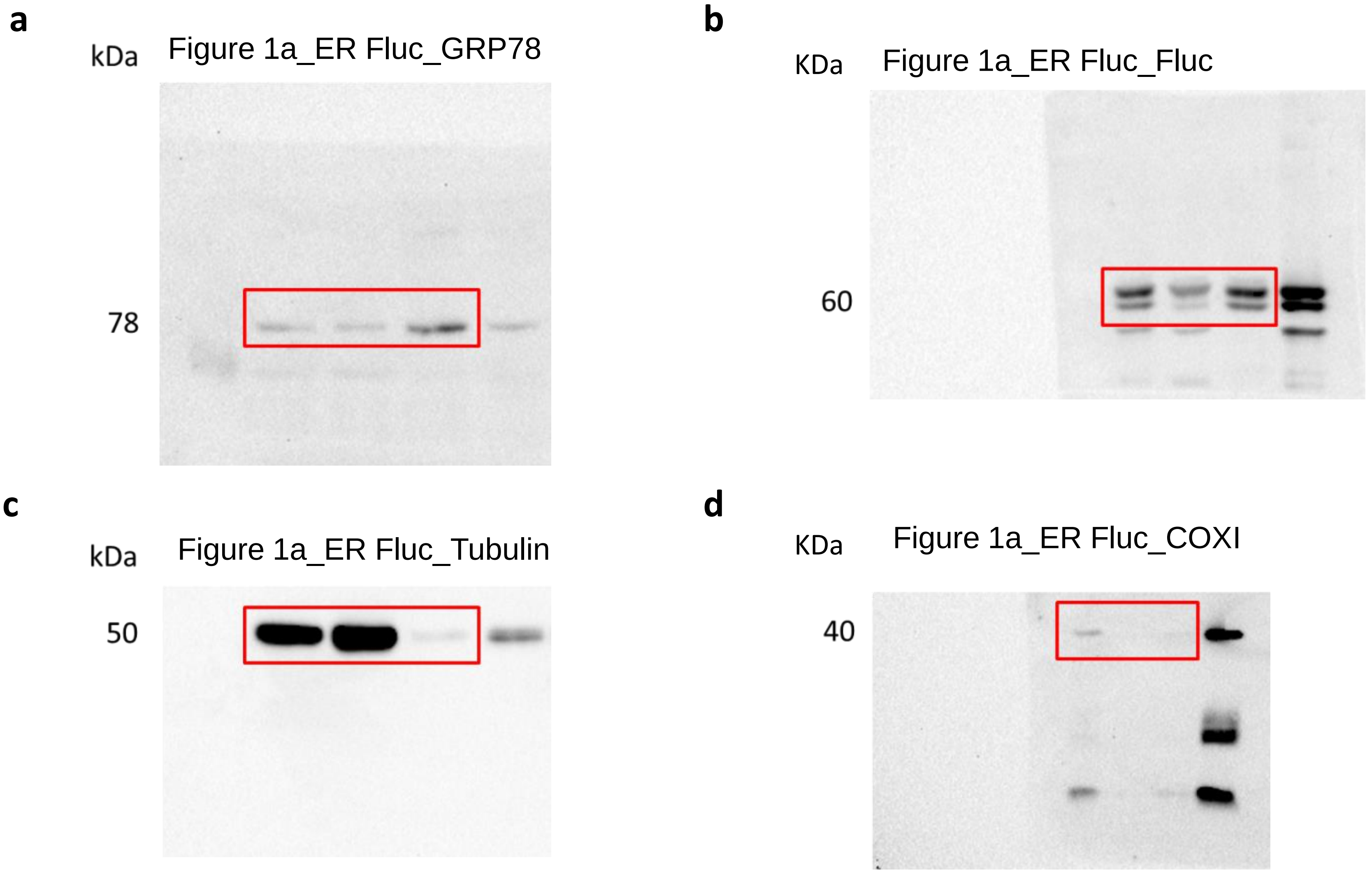

Supplementary Figure 16B. Uncropped Western blots used for Fig. 1a: HEK 293 cells transfected with ER-Fluc. Red frames show the protein bands used in the figure. Due to similar protein size, sample replicates were run on two different gels, blotted, membranes cut and incubated with different antibodies: a) GRP78, b) Fluc, c) Tubulin, and d) COXI. GRP78 and Tubulin were from the same gel, Fluc and COXI were from the same gel.

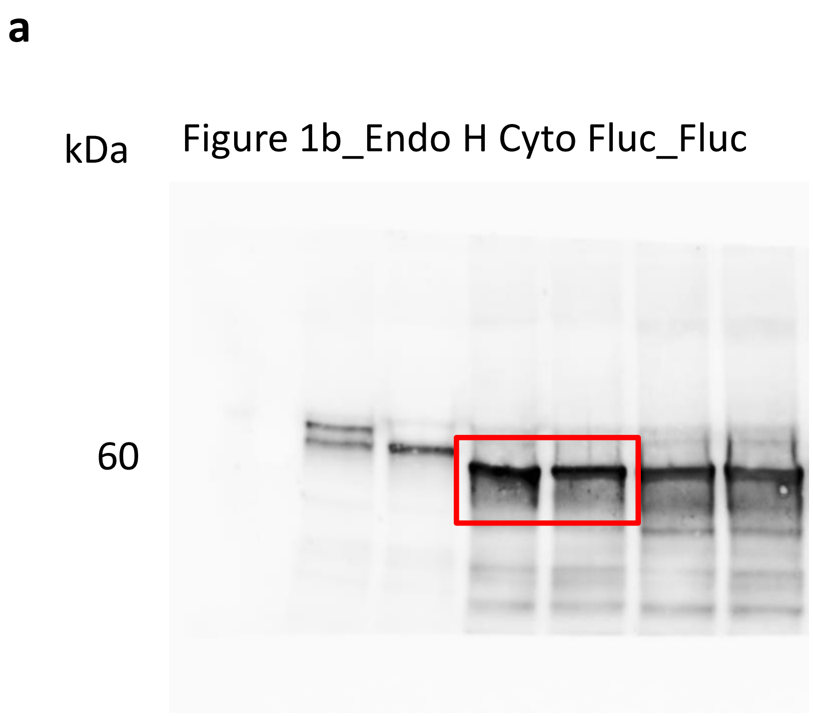

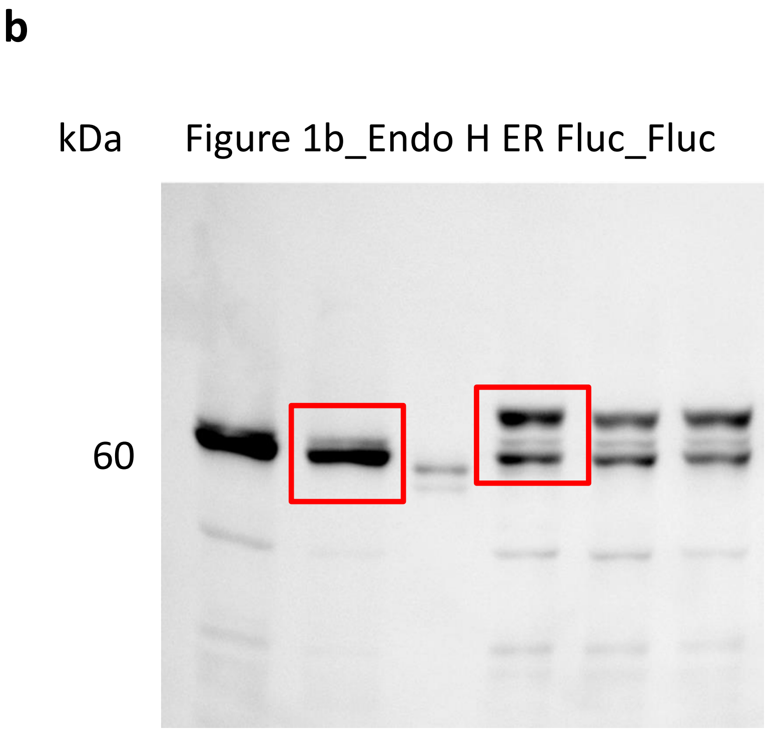

Supplementary Figure 16C. Uncropped Western blots used for Fig. 1b: HEK 293 cells transfected with cyto-Fluc and ER-Fluc. Red frames show the protein bands used in the figure. Samples were run on two different gels as indicated, blotted, membranes cut and incubated with Fluc antibody a), b).

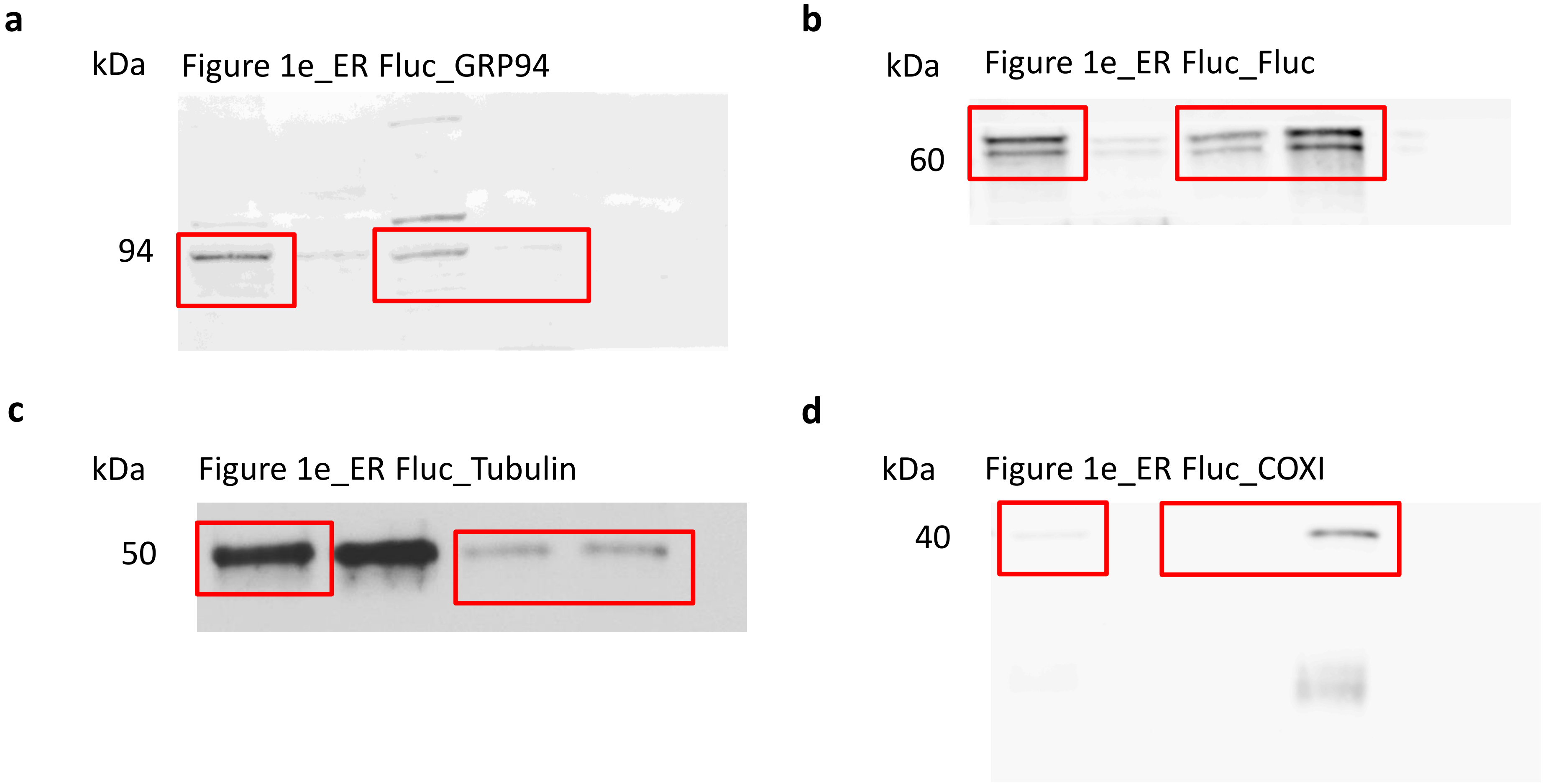

Supplementary Figure 16D. Uncropped Western blots used for Fig. 1e: HEK 293 cells transfected with ER-Fluc. Red frames show the protein bands used in the figure. Samples were run on the same gel, blotted, membrane cut and incubated with different antibodies: a) GRP94, b) Fluc, c) Tubulin, and d) COXI.

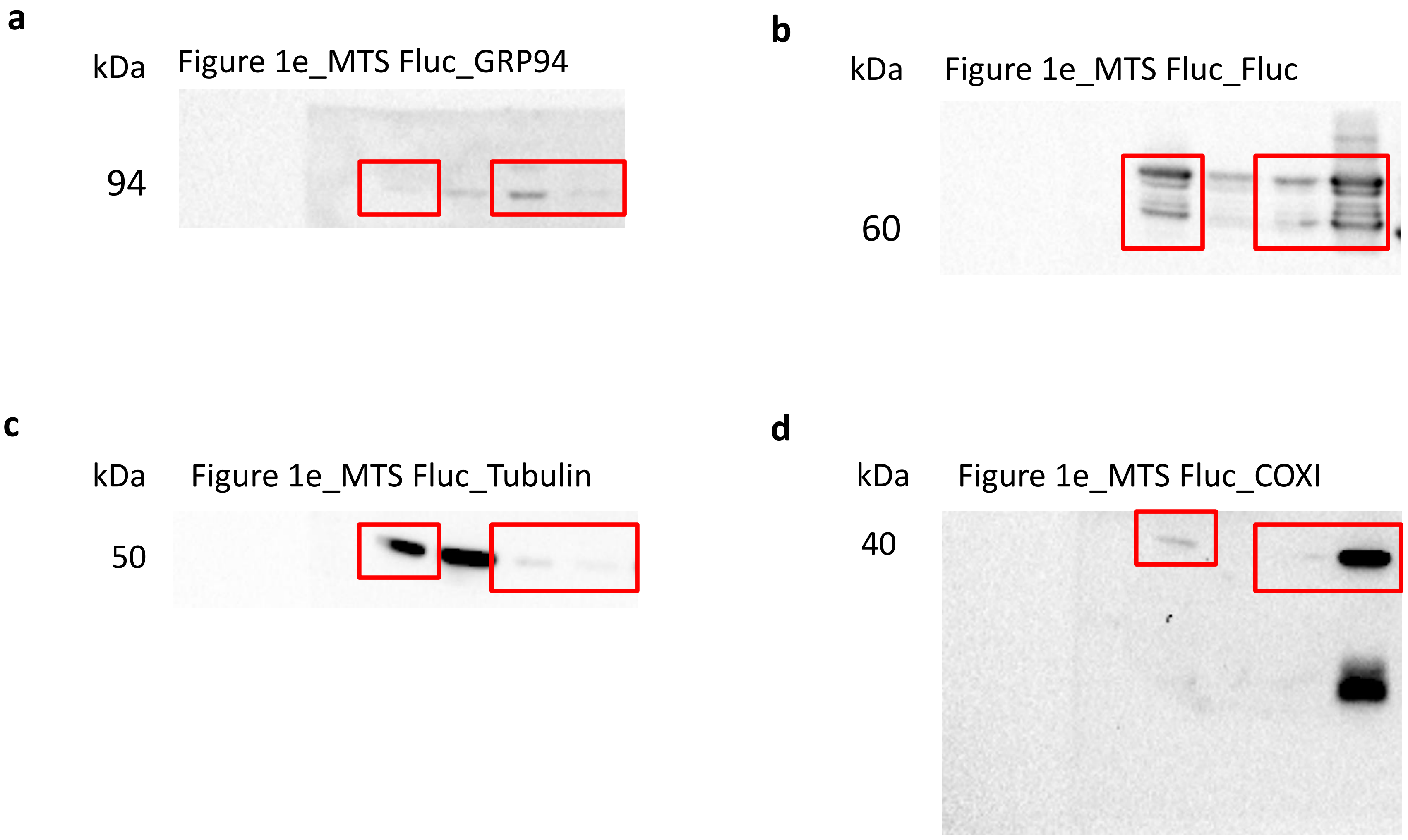

Supplementary Figure 16E. Uncropped Western blots used for Fig. 1e: HEK 293 cells transfected with MTS-Fluc. Red frames show the protein bands used in the figure. Samples were run on the same gel, blotted, membrane cut and incubated with different antibodies: a) GRP94, b) Fluc, c) Tubulin, and d) COXI.

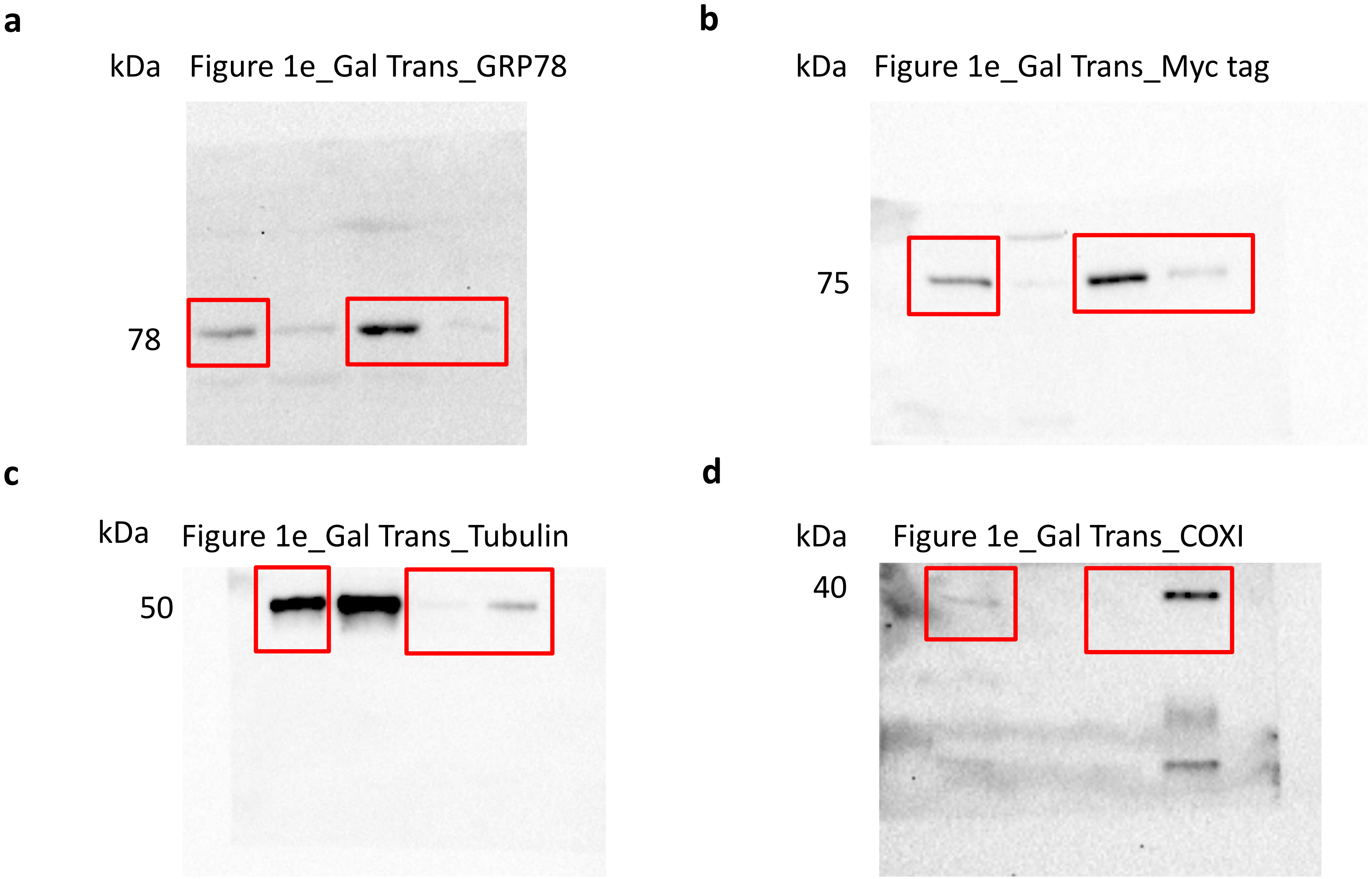

Supplementary Figure 16F. Uncropped Western blots used for Fig. 1e: HEK 293 cells transfected with Gal-Trans. Red frames show the protein bands used in the figure. Due to similar protein size, samples replicates were run on two different gels, blotted, membranes cut and incubated with different antibodies: a) GRP78, b) Myc-tag, c) Tubulin, and d) COXI. GRP78, Tubulin and COXI were from the same gel.

a b

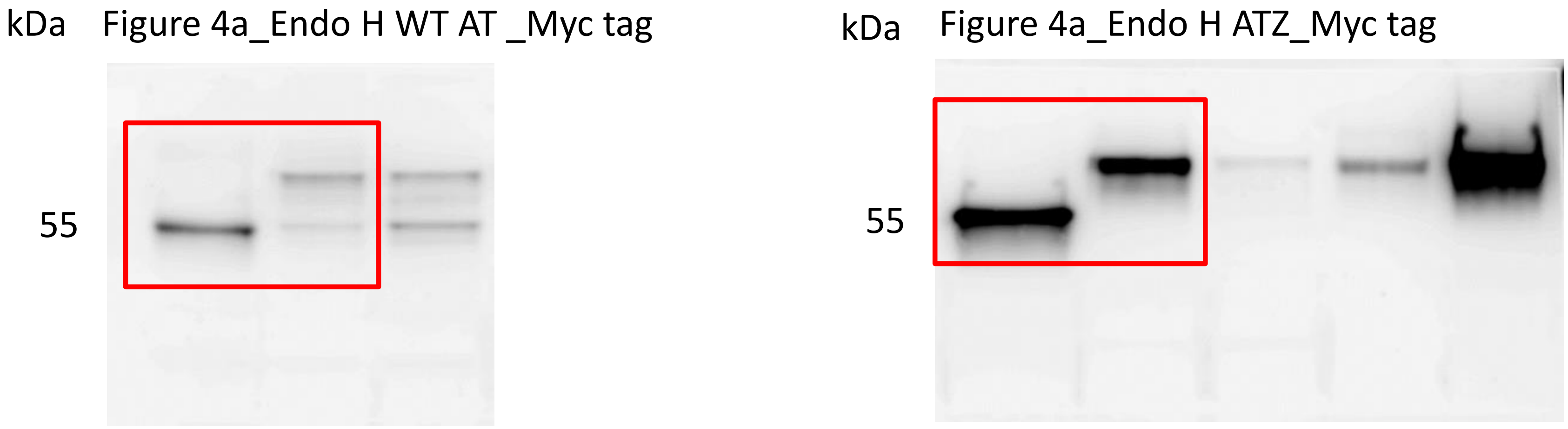

Supplementary Figure 17A. Uncropped Western blots used for Fig. 4a: HEK 293 cells transfected with WT and MUT α-1-antitrypsin. Red frames show the protein bands used in the figure. Samples were run on two different gels as indicated, blotted, membranes cut and incubated with Myc-tag antibody a), b).

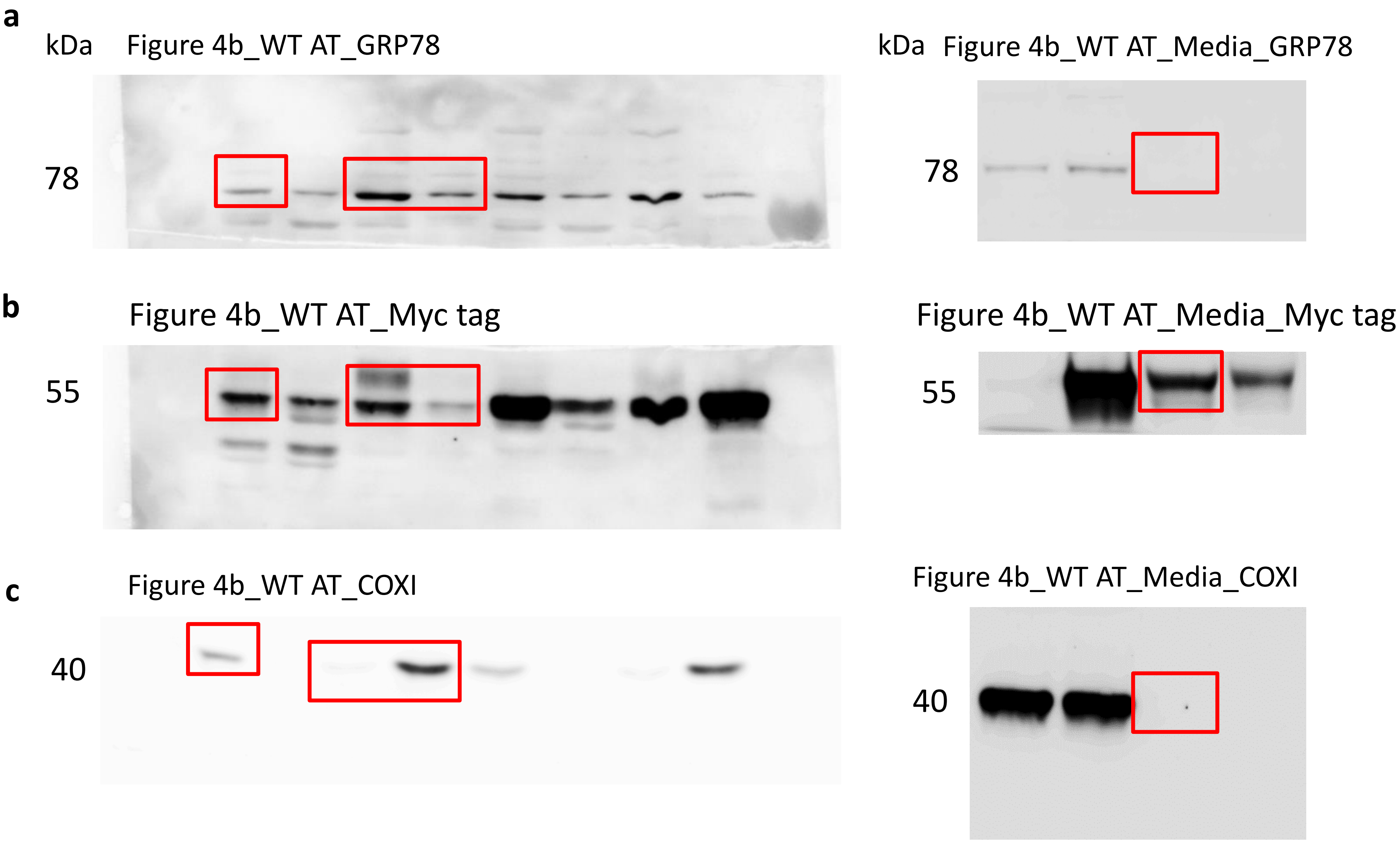

Supplementary Figure 17B. Uncropped Western blots used for Fig. 4b: HEK 293 cells transfected with WT α-1-antitrypsin. Red frames show the protein bands used in the figure. Samples and media were run on two different gels, blotted, membranes cut and incubated with different antibodies: a) GRP78, b) Myc-tag, and c) COXI. GRP78, Myc-tag and COXI of WT α-1-antitrypsin were from the same gel. GRP78, Myc-tag and COXI of media were from the same gel.

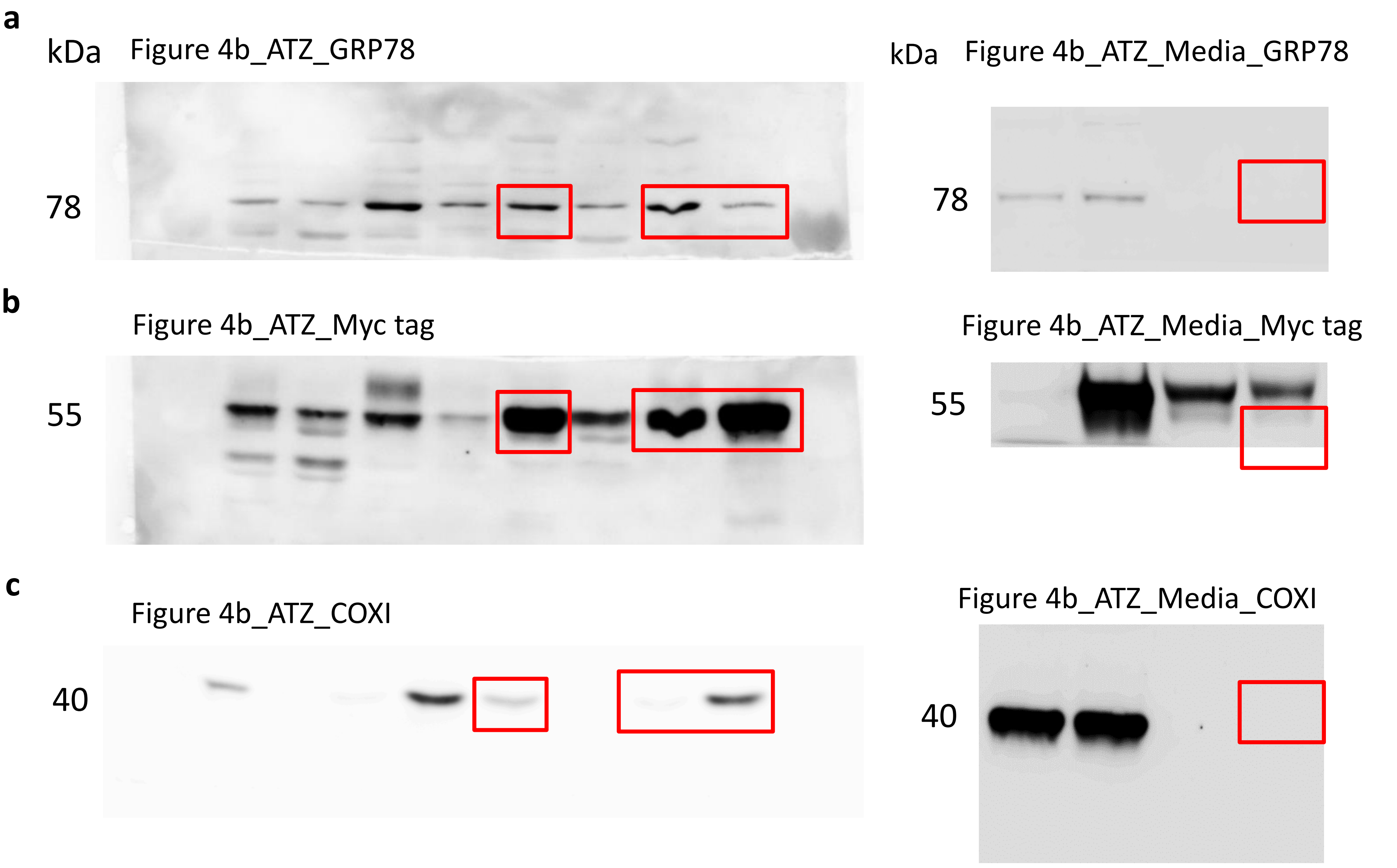

Supplementary Figure 17C. Uncropped Western blots used for Fig. 4b: HEK 293 cells transfected with MUT α-1-antitrypsin. Red frames show the protein bands used in the figure. Samples and media were run on two different gels, blotted, membranes cut and incubated with different antibodies: a) GRP78, b) Myc-tag, and c) COXI. GRP78, Myc-tag and COXI of MUT α-1-antitrypsin were from the same gel. GRP78, Myc-tag and COXI of media were from the same gel.

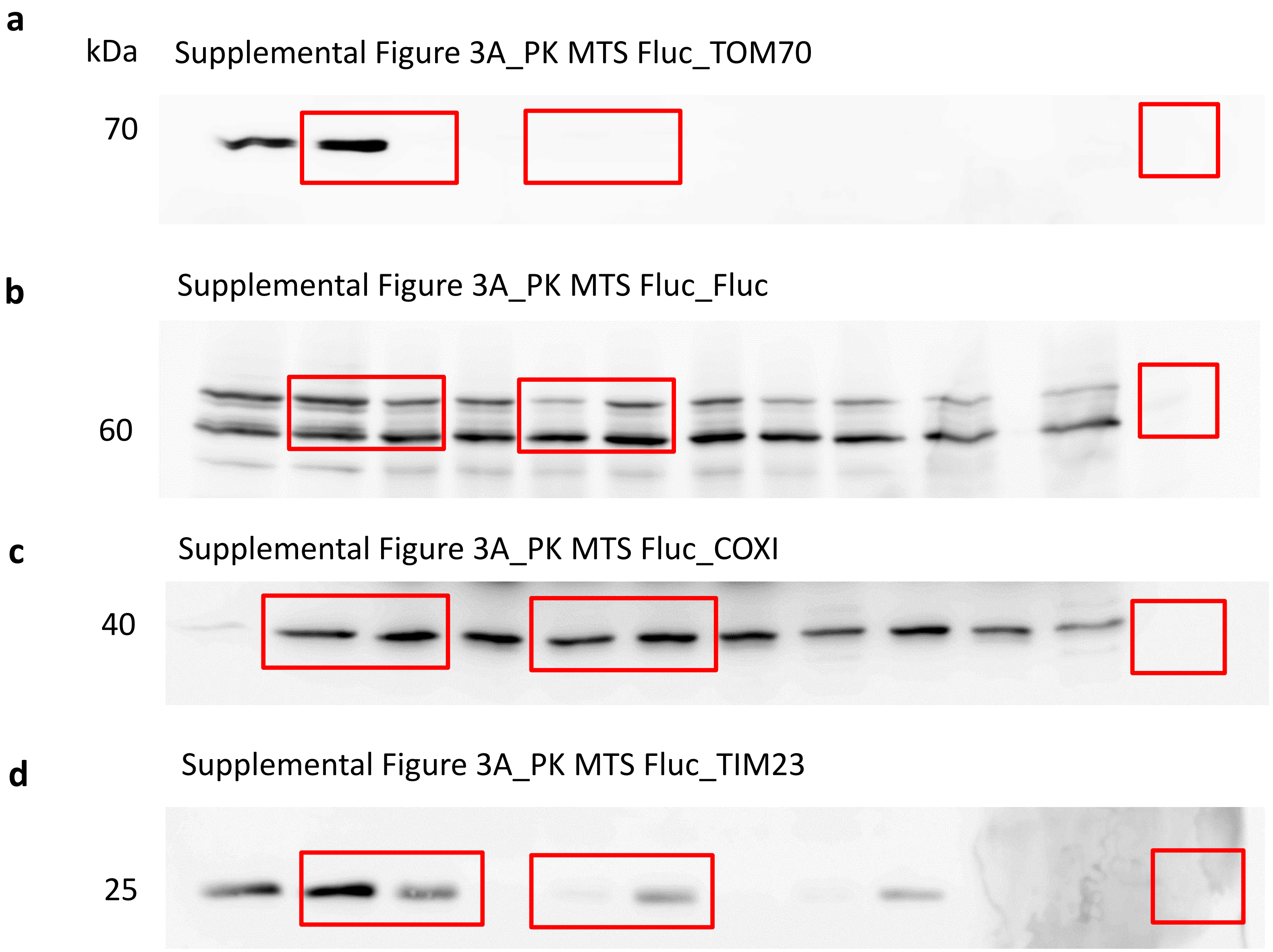

Supplementary Figure 18A. Uncropped Western blots used for Supplemental Fig. 3A: HEK 293 cells transfected with MTS-Fluc. Red frames show the protein bands used in the figure. Due to similar protein size, samples replicates were run on two different gels, blotted, membranes cut and incubated with different antibodies: a) TOM70, b) Fluc, c) COXI, and d) TIM23. Fluc and TIM23 were from the same gel, TOM70 and COXI were from the same gel.

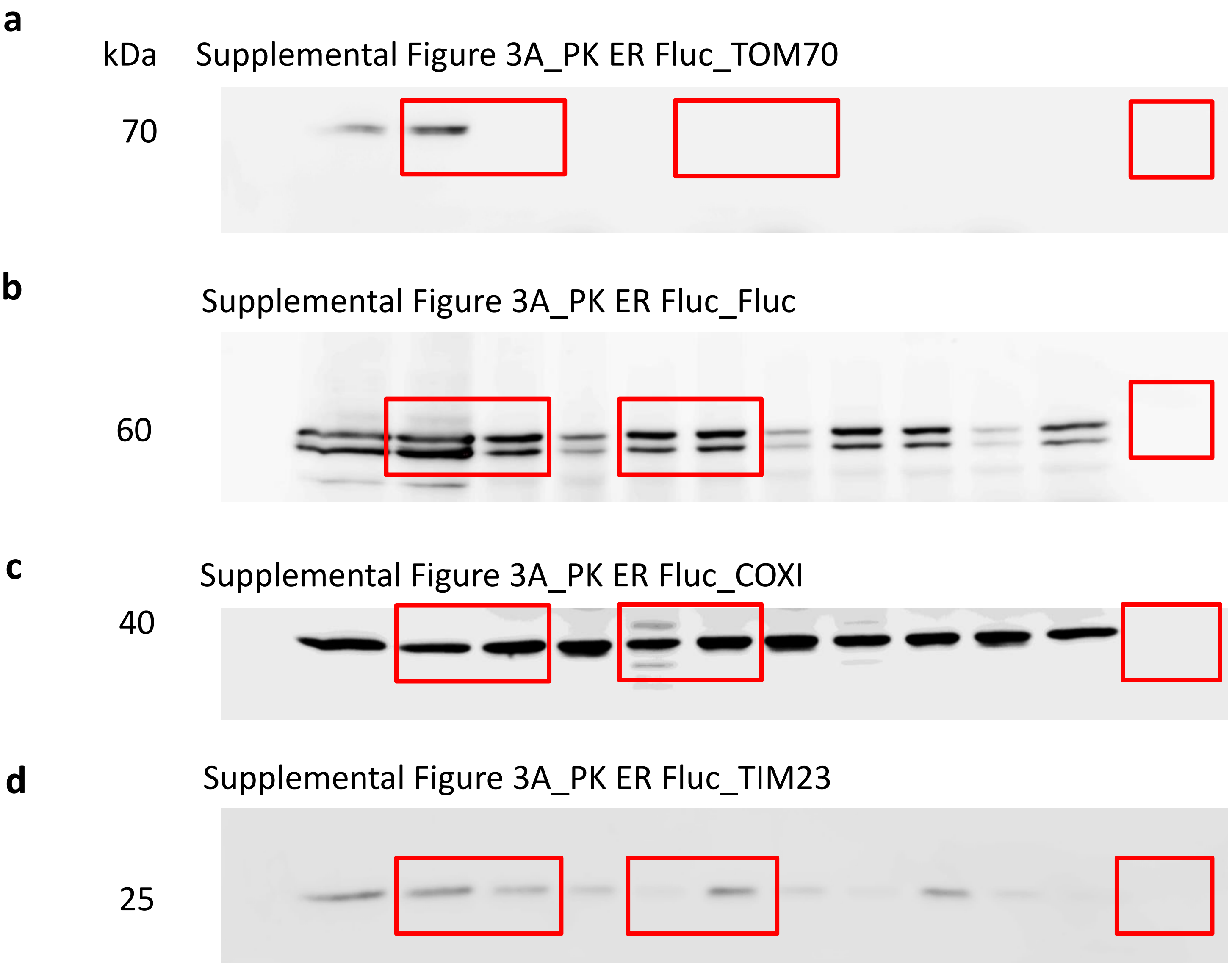

Supplementary Figure 18B. Uncropped Western blots used for Supplemental Fig. 3A: HEK 293 cells transfected with ER-Fluc. Red frames show the protein bands used in the figure. Due to similar protein size, samples replicates were run on different gels, membranes cut and incubated with different antibodies: a) TOM70, b) Fluc, c) COXI, and d) TIM23. Fluc and TIM23 were from the same gel. TOM70 and COXI were from the same gel.

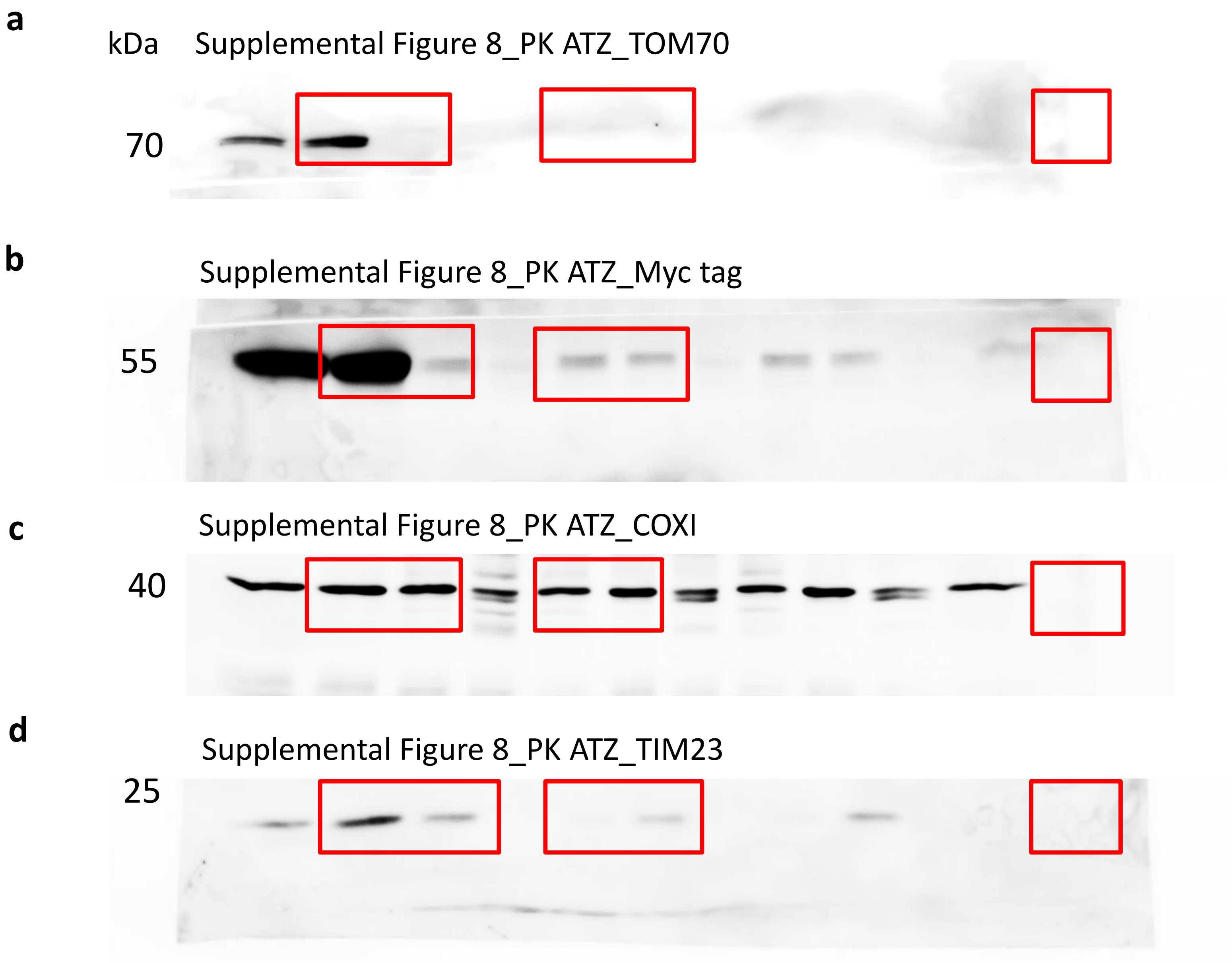

Supplementary Figure 19. Uncropped Western blots used for Supplementar Fig. 8: HEK 293 cells transfected with WT α-1-antitrypsin. Red frames show the protein bands used in the figure. Samples were run on the same gel, blotted, membrane cut and incubated with different antibodies: a) TOM70, b) Myc-tag, c) COXI, and d) TIM23.

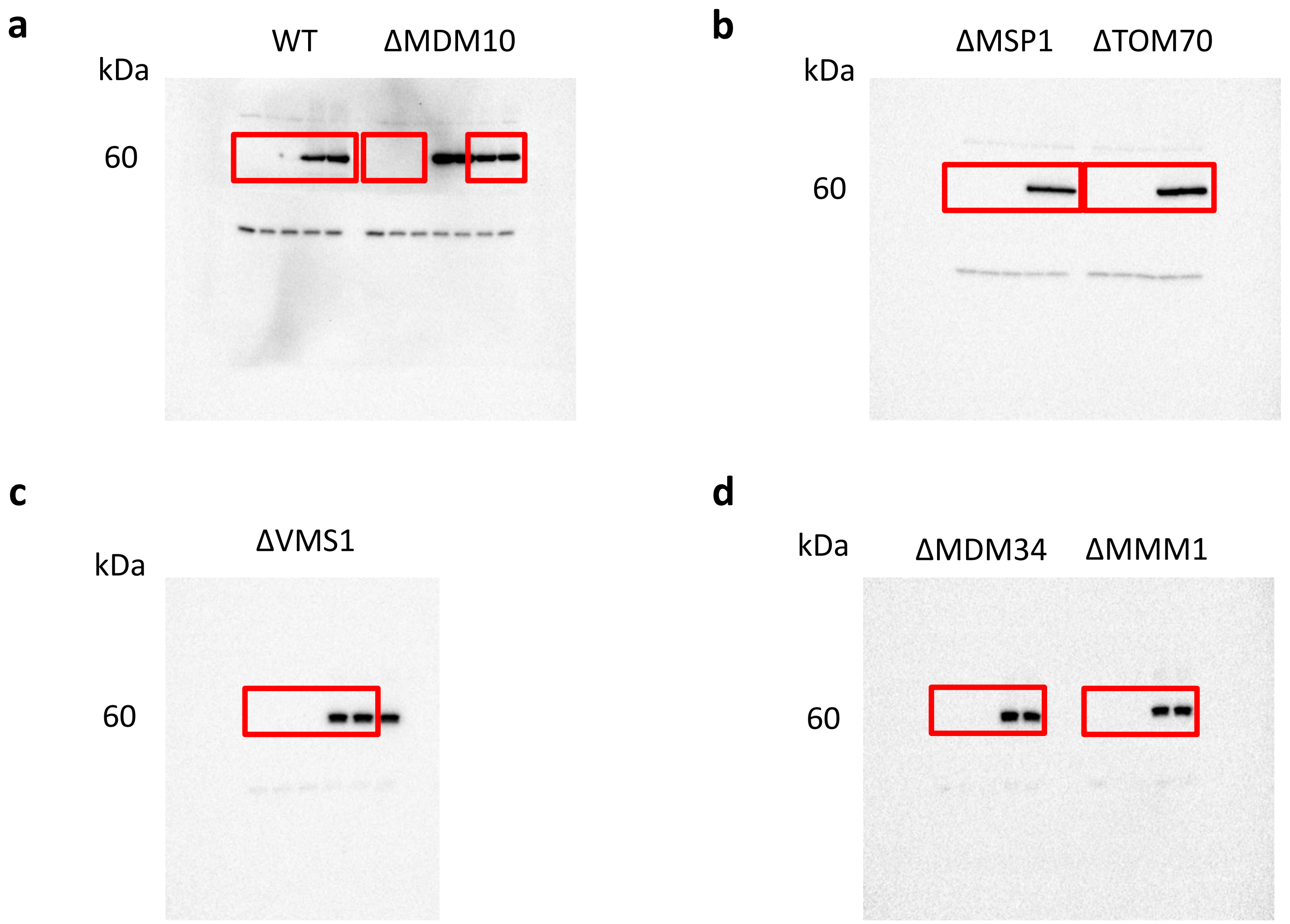

Supplementary Figure 20A. Uncropped Western blots used for Supplemental Fig. 12A. Fluc detection in yeast WT and deletion mutants expressing yER-FlucDM-GFP_{11} and yMTS-mCherry-$GFP_{1\text{-}10}$. Red frames show the protein bands used in the figure. Samples were run on 4 different gels, blotted, and membranes incubated with HA-tag antibody for Fluc detection. Note: The lower band in a) and b) is DpmI.

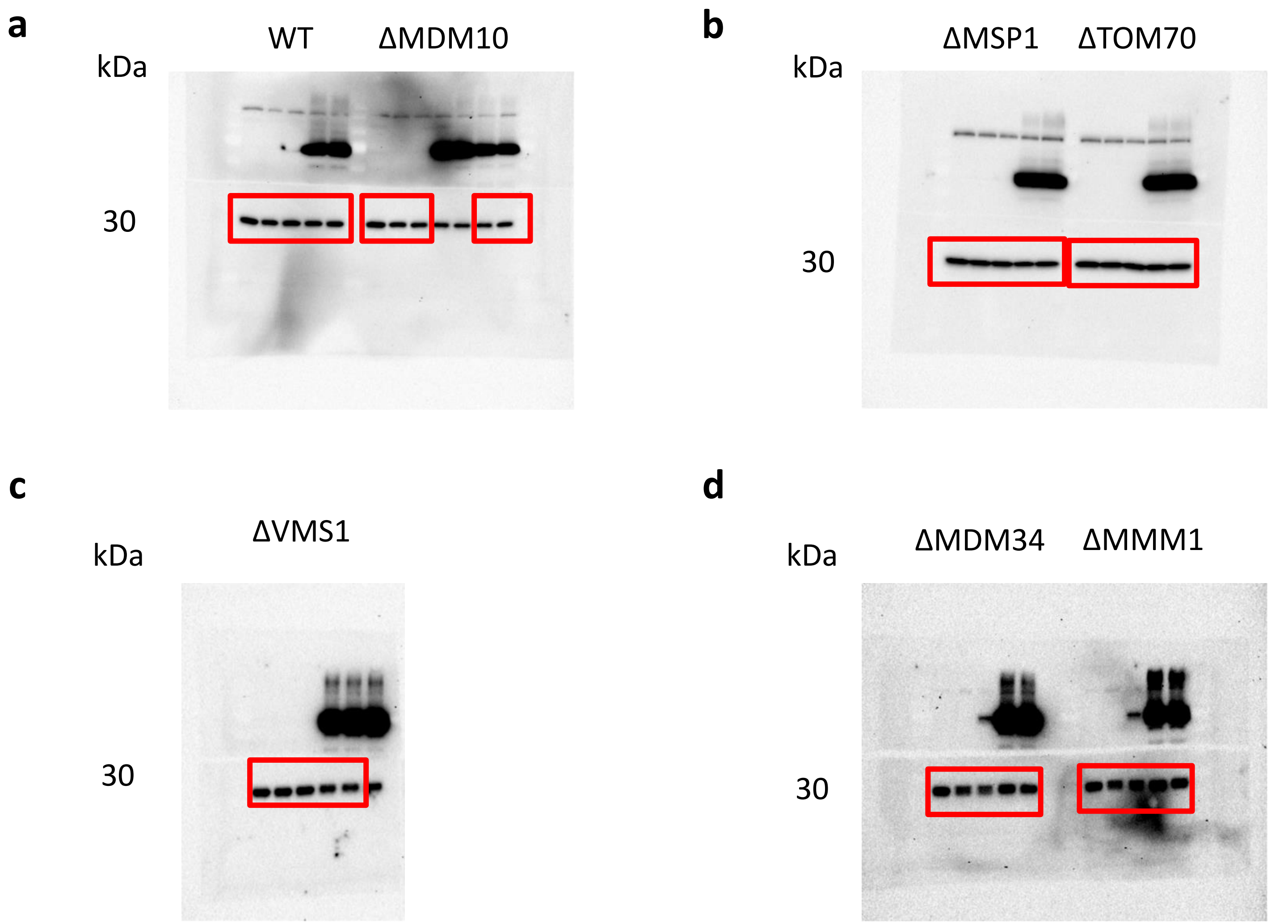

Supplementary Figure 20B. Uncropped Western blots used for Supplemental Fig. 12A. DpmI detection in yeast WT and deletion mutants expressing yER-FlucDM-GFP$_{11}$ and yMTS-mCherry-GFP$_{1\text{-}10}$. Red frames show the protein bands used in the figure. Samples were run on 4 different gels, blotted, membranes cut and incubated with DpmI antibody. Note: The upper band in a), b), c) and d) is Fluc. (There is some spill over of Fluc into neighboring wells for d)).

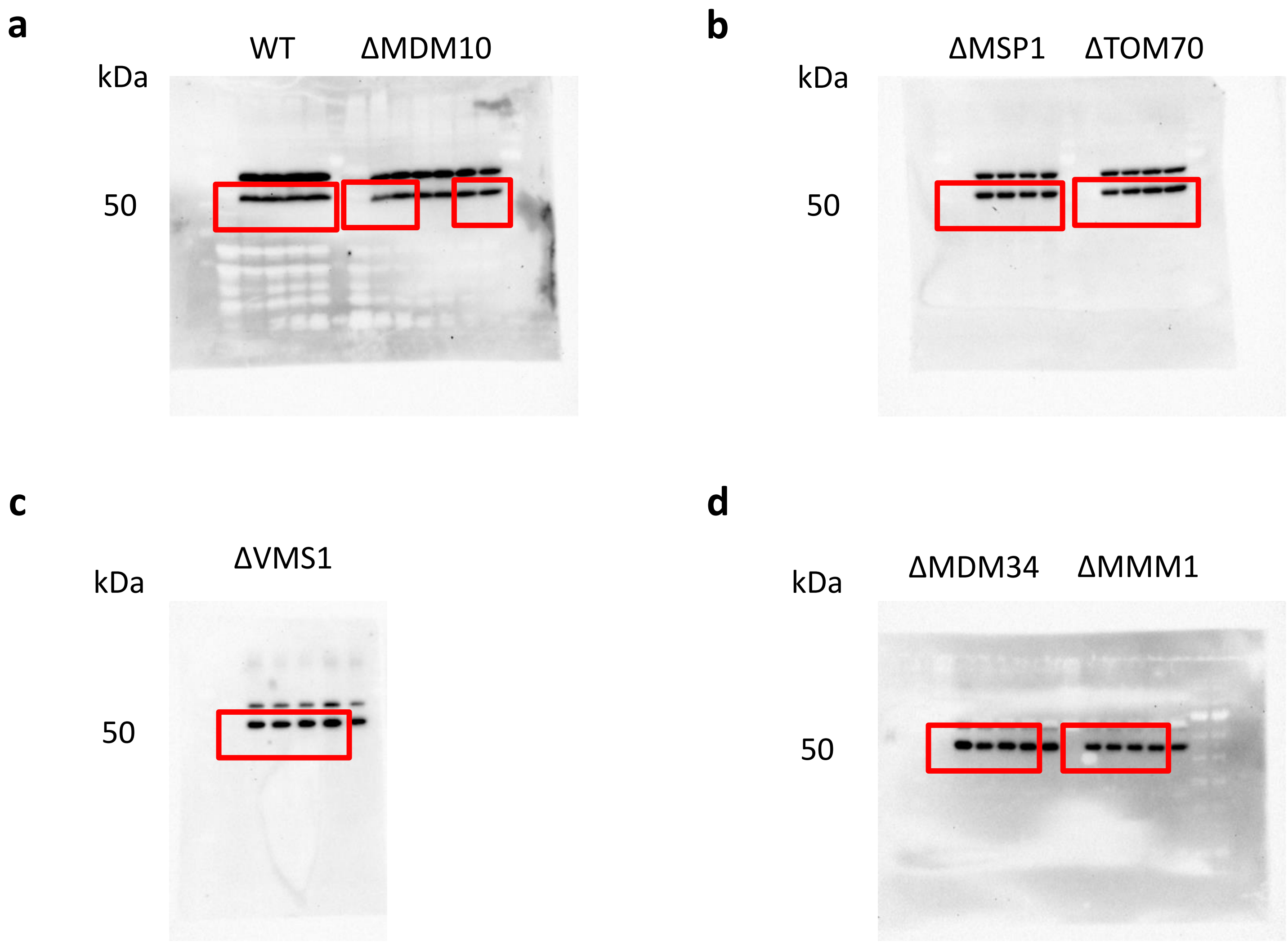

Supplementary Figure 20C. Uncropped Western blots used for Supplemental Fig. 12A. mCherry detection in yeast WT and deletion mutants expressing yER-FlucDM-GFP_{11} and yMTS-mCherry-GFP_{1-10}. Red frames show the protein bands used in the figure. Samples were run on 4 different gels, blotted, and membranes incubated with mCherry antibody.

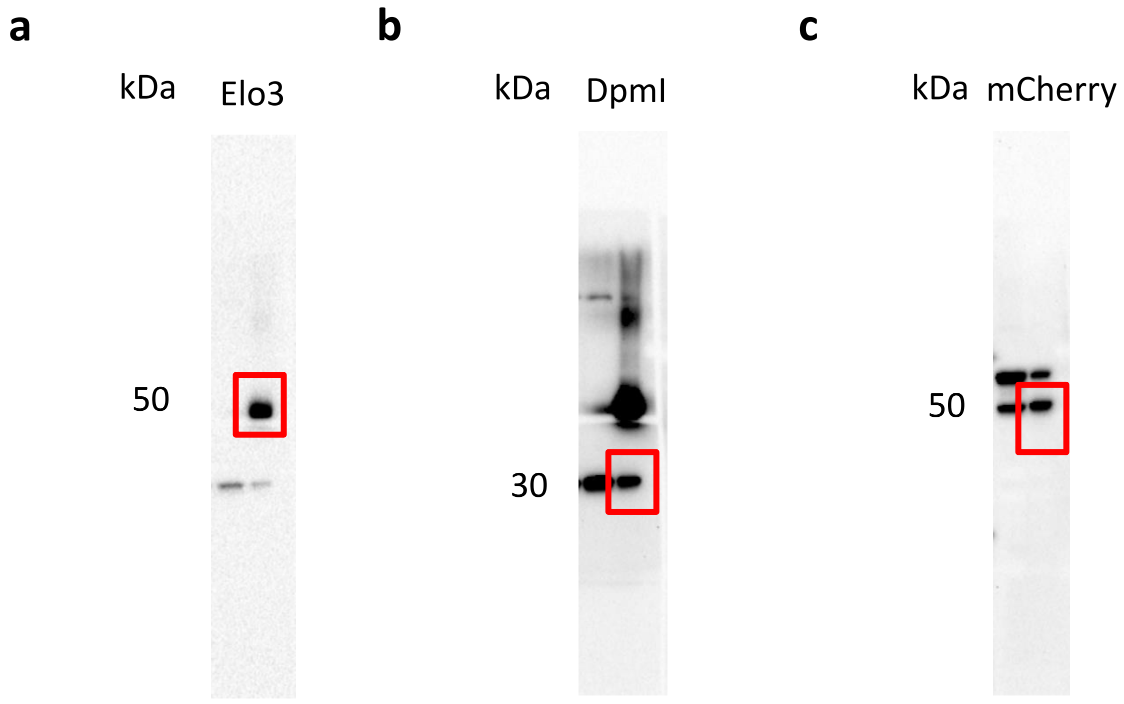

Supplementary Figure 20D. Uncropped Western blots used for Supplemental Fig. 12B. Detection of proteins in yeast WT and deletion mutants expressing yElo3-GFP$_{11}$ and yMTS-mCherry-GFP$_{1\text{-}10}$. Red frames show the protein bands used in the figure. Samples were run on the same gel, blotted, membrane cut and incubated with different antibodies: a) HA-tag for Elo3 detection, b) DpmI and c) mCherry. Note: The lower band in a) is DpmI. The higher band in b) is Elo3.

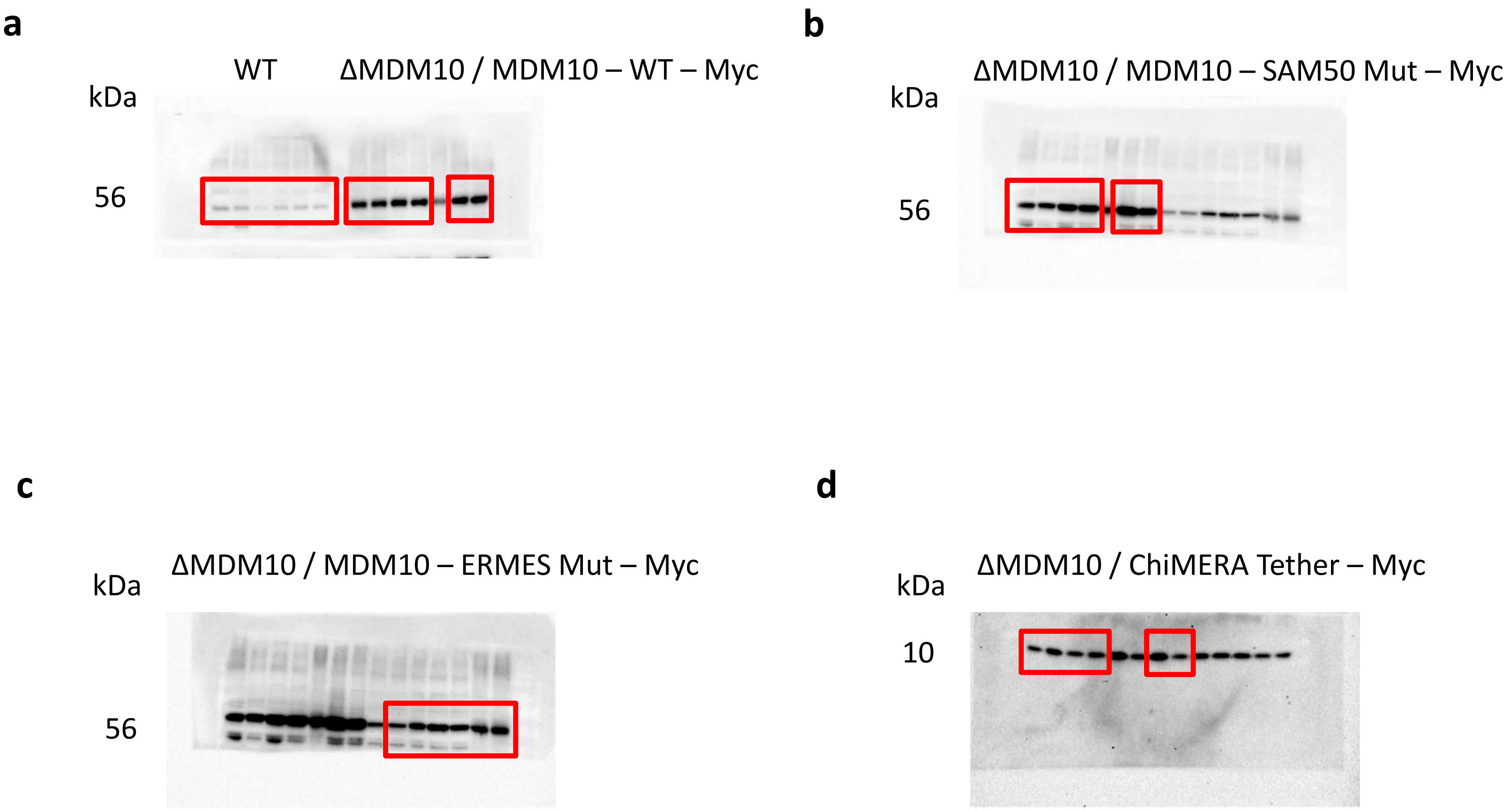

Supplementary Figure 21A. Uncropped Western blots used for Supplemental Fig. 13. Detection of indicated complementing Myc-tagged proteins in yeast WT and MDM10 deletion mutants expressing yER-FlucDM-GFP_{11} and yMTS-mCherry-$GFP_{1\text{-}10}$. Red frames show the protein bands used in the figure. Samples were run on 4 different gels, blotted, membranes cut and incubated with Myc-tag antibody.

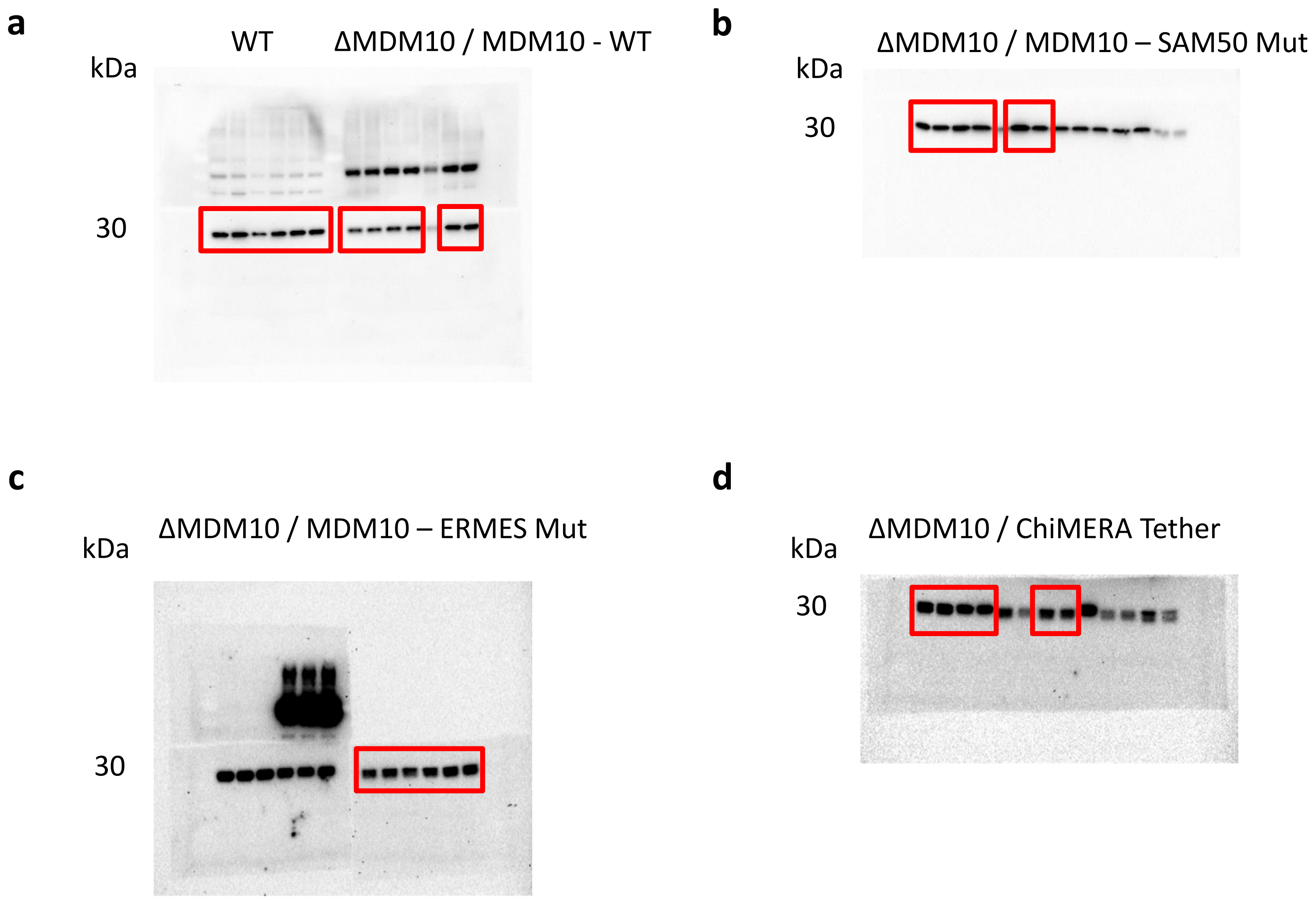

Supplementary Figure 21B. Uncropped Western blots used for Supplemental Fig. 13. DpmI detection in yeast WT and MDM10 deletion mutants expressing yER-FlucDM-GFP_{11} and yMTS-mCherry-$GFP_{1\text{-}10}$. Red frames show the protein bands used in the figure. Samples were run on 4 different gels, blotted, membranes cut and incubated with DpmI antibody.

Supplementary Table 1: List of plasmids used in this study for transfection of HEK293 cells.

Plasmid	Addgene ID	Reporter	Tag	Plasmid description
phFluc-WT	177714	Firefly luciferase (WT)	-	Cytosolic firefly luciferase reporter previously described in Shcherbakov et al. 2019
pPLN-hFluc-WT-KDEL	177715	Firefly luciferase (WT)	-	Firefly luciferase reporter fused to a prolactin signal sequence for ER targeting and KDEL for ER retention
pMTS-hFluc	177716	Firefly luciferase (WT)	-	Firefly luciferase reporter fused to a mitochondria signal sequence for mitochondrial targeting
pAT-WT-Myc	177717	Alpha-1-antitrypsin (WT)	Myc	ER protein alpha-1-antitrypsin (WT), myc tagged
pATZ-Myc	177718	Alpha-1-antitrypsin (MUT)	Myc	ER protein alpha-1-antitrypsin containing E342K mutation (ATZ mutant), myc tagged
pAT-WT-HA-GFP_{11}	177719	Alpha-1-antitrypsin (WT); GFP_{11}	HA	ER protein alpha-1-antitrypsin (WT) fused to an HA tag and the eleventh β-strand of GFP
pATZ-HA-GFP_{11}	177720	Alpha-1-antitrypsin (MUT); GFP_{11}	HA	ER protein alpha-1-antitrypsin containing E342K mutation (ATZ mutant) fused to an HA tag and the eleventh β-strand of GFP
pATZ-BFP-HA-GFP_{11}	177721	Alpha-1-antitrypsin (MUT); BFP; GFP_{11}	HA	ER protein alpha-1-antitrypsin containing E342K mutation (ATZ mutant) fused to blue fluorescence protein (BFP) tag protein, an HA tag and the eleventh β-strand of GFP
pPLN-hFluc-WT-HA-GFP_{11}-KDEL	177722	Firefly luciferase (WT); GFP_{11}	HA	Firefly luciferase reporter fused to a prolactin signal sequence for ER targeting and KDEL for ER retention with an HA tag and the eleventh β-strand of GFP added to the C-terminus
pPLN-hFluc-DM-HA-GFP_{11}-KDEL	177723	Firefly luciferase (DM); GFP_{11}	HA	DM Firefly luciferase reporter fused to a prolactin signal sequence for ER targeting and KDEL for ER retention with an HA tag and the eleventh β-strand of GFP added to the C-terminus
pGal-Trans-Myc-HA-GFP_{11}-KDEL	177724	Collagen beta (1-O) galactosyltransferase; GFP_{11}	Myc and HA	ER protein collagen beta (1-O) galactosyltransferase with a C-terminal Myc tag, an HA tag and the eleventh β-strand of GFP
pMTS-mCherry-GFP_{1-10}	-	mCherry; GFP_{1-10}	-	mCherry and the 1-10th β-strands of GFP (GFP_{1-10}) fused to a mitochondria signal sequence for mitochondrial targeting
pMTS-HA-GFP_{11}	177725	GFP_{11}	HA	Eleventh β-strand of GFP (GFP_{11}) fused to a mitochondria signal sequence for mitochondrial targeting, HA tagged
pMTS-hFluc-HA-GFP_{11}	177726	Firefly luciferase (WT); GFP_{11}	HA	Firefly luciferase reporter fused to a mitochondria signal sequence for mitochondrial targeting with an HA tag and the eleventh β-strand of GFP added to the C-terminus
pPLN-hFluc-WT-eGFP-KDEL	177727	Firefly luciferase (WT); eGFP	-	Firefly luciferase reporter fused to a prolactin signal sequence for ER targeting and KDEL for ER retention and fused to eGFP at the C-terminus
pPLN-hFluc-DM-eGFP-KDEL	177728	Firefly luciferase (DM); eGFP	-	DM Firefly luciferase reporter fused to a prolactin signal sequence for ER targeting and KDEL for ER retention and fused to eGFP at the C-terminus
pGal-Trans-Myc-—eGFP-KDEL	177729	Collagen beta (1-O) galactosyltransferase; eGFP	Myc	ER protein collagen beta (1-O) galactosyltransferase fused to eGFP at the C-terminus

Supplementary Table 2: List of plasmids used in this study for yeast transformation.

Plasmid	Addgene ID	Insert Gene	Source
p403 – TDH3-His	-	-	Addgene
p405 – TDH3-Leu	-	-	Addgene
p406 – TDH3-Ura	-	-	Addgene
pM - Met	-	-	Addgene
p403 – TDH3-MTS-mCherry-GFP_{1-10}-His	177730	MTS-mCherry-GFP_{1-10}	This Study
p406 – TDH3-ER-FlucDM-GFP_{11}-Ura	177731	KAR2SS-FlucDM-HA-GFP_{11}-HDEL	This Study
p406 – TDH3-ER-FlucDM-GFP-Ura	177732	KAR2SS-FlucDM-GFP-HDEL	This Study
p406 – TDH3-MTS-GFP_{11}-Ura	177733	MTS-GFP_{11}	This Study
p406 – TDH3-Elo3-GFP_{11}-Ura	177734	Elo3-GFP_{11}	This Study
p1k-Elo3-mCherry-Trp	-	Elo3-mCherry	Addgene
p1k-Elo3-mCherry-His	-	Elo3-mCherry	This Study
pM – TDH3-MDM10 WT-Myc-Met	-	MDM10 WT-Myc	This Study
pM – TDH3-MDM10 Y73,75A-Myc-Met (MDM10 SAM50 Mut)	-	MDM10 Y73,75A-Myc	This Study
pM – TDH3-MDM10 Y293,F298A-Myc-Met (MDM10 ERMES Mut)	-	MDM10 Y293,F298A-Myc	This Study
pM – TDH3-ChiMERA Tether-Myc-Met (ChiMERA Tether)	-	ChiMERA Tether	Kornmann *et al*., 2009

Supplementary Table 3: List of yeast strains used in this study.

Strain	Genetic Background	Selection Media	Source
BY4741	*MATa his3Δ1 leu2Δ0 met15Δ0 ura3Δ0*	YPD/SD complete	Euroscarf
yMTS-mCherry-GFP$_{1-10}$	*MATa leu2Δ0 met15Δ0 ura3Δ0; pTDH-MTS-mCherry-GFP$_{1-10}$-HIS3*	SD His$^-$	This Study
yElo3-GFP$_{11}$;<br>yMTS-mCherry-GFP$_{1-10}$	*MATa leu2Δ0 met15Δ0; pTDH-Elo3-GFP$_{11}$-HDEL-URA3; pTDH-MTS-mCherry-GFP$_{1-10}$-HIS3*	SD Ura$^-$ His$^-$	This Study
yMTS-GFP$_{11}$;<br>yMTS-mCherry-GFP$_{1-10}$	*MATa leu2Δ0 met15Δ0; pTDH-MTS-GFP$_{11}$-URA3; pTDH-MTS-mCherry-GFP$_{1-10}$-HIS3*	SD Ura$^-$ His$^-$	This Study
yER-FlucDM-GFP$_{11}$;<br>yMTS-mCherry-GFP$_{1-10}$	*MATa leu2Δ0 met15Δ0; pTDH-KAR2SS-FlucDM-HA-GFP$_{11}$-HDEL-URA3; pTDH-MTS-mCherry-GFP$_{1-10}$-HIS3*	SD Ura$^-$ His$^-$	This Study
ΔMSP1<br>yMTS-mCherry-GFP$_{1-10}$	*MATa met15Δ0 ura3Δ0; ΔMSP1::LEU2; pTDH-MTS-mCherry-GFP$_{1-10}$-HIS3*	SD Leu$^-$ His$^-$	This Study
ΔMSP1<br>yMTS-GFP$_{11}$;<br>yMTS-mCherry-GFP$_{1-10}$	*MATa met15Δ0; ΔMSP1::LEU2; pTDH-MTS-GFP$_{11}$-URA3; pTDH-MTS-mCherry-GFP$_{1-10}$-HIS3*	SD Leu$^-$ Ura$^-$ His$^-$	This Study
ΔMSP1<br>yER-FlucDM-GFP$_{11}$;<br>yMTS-mCherry-GFP$_{1-10}$	*MATa met15Δ0; ΔMSP1::LEU2; pTDH-KAR2SS-FlucDM-HA-GFP$_{11}$-HDEL-URA3; pTDH-MTS-mCherry-GFP$_{1-10}$-HIS3*	SD Leu$^-$ Ura$^-$ His$^-$	This Study
ΔTOM70<br>yMTS-mCherry-GFP$_{1-10}$	*MATa met15Δ0 ura3Δ0; ΔTOM70::LEU2; pTDH-MTS-mCherry-GFP$_{1-10}$-HIS3*	SD Leu$^-$ His$^-$	This Study
ΔTOM70<br>yMTS-GFP$_{11}$;<br>yMTS-mCherry-GFP$_{1-10}$	*MATa met15Δ0; ΔTOM70::LEU2; pTDH-MTS-GFP$_{11}$-URA3; pTDH-MTS-mCherry-GFP$_{1-10}$-HIS3*	SD Leu$^-$ Ura$^-$ His$^-$	This Study
ΔTOM70<br>yER-FlucDM-GFP$_{11}$;<br>yMTS-mCherry-GFP$_{1-10}$	*MATa met15Δ0; ΔTOM70::LEU2; pTDH-KAR2SS-FlucDM-HA-GFP$_{11}$-HDEL-URA3; pTDH-MTS-mCherry-GFP$_{1-10}$-HIS3*	SD Leu$^-$ Ura$^-$ His$^-$	This Study
ΔVMS1<br>yMTS-mCherry-GFP$_{1-10}$	*MATa met15Δ0 ura3Δ0; ΔVMS1::LEU2; pTDH-MTS-mCherry-GFP$_{1-10}$-HIS3*	SD Leu$^-$ His$^-$	This Study
ΔVMS1<br>yMTS-GFP$_{11}$;<br>yMTS-mCherry-GFP$_{1-10}$	*MATa met15Δ0; ΔVMS1::LEU2; pTDH-MTS-GFP$_{11}$-URA3; pTDH-MTS-mCherry-GFP$_{1-10}$-HIS3*	SD Leu$^-$ Ura$^-$ His$^-$	This Study
ΔVMS1<br>yER-FlucDM-GFP$_{11}$;<br>yMTS-mCherry-GFP$_{1-10}$	*MATa met15Δ0; ΔVMS1::LEU2; pTDH-KAR2SS-FlucDM-HA-GFP$_{11}$-HDEL-URA3; pTDH-MTS-mCherry-GFP$_{1-10}$-HIS3*	SD Leu$^-$ Ura$^-$ His$^-$	This Study
ΔMDM10<br>yMTS-mCherry-GFP$_{1-10}$	*MATa met15Δ0 ura3Δ0; ΔMDM10::LEU2; pTDH-MTS-mCherry-GFP$_{1-10}$-HIS3*	SD Leu$^-$ His$^-$	This Study
ΔMDM10<br>yMTS-GFP$_{11}$;<br>yMTS-mCherry-GFP$_{1-10}$	*MATa met15Δ0; ΔMDM10::LEU2; pTDH-MTS-GFP$_{11}$-URA3; pTDH-MTS-mCherry-GFP$_{1-10}$-HIS3*	SD Leu$^-$ Ura$^-$ His$^-$	This Study
ΔMDM10<br>yER-FlucDM-GFP$_{11}$;<br>yMTS-mCherry-GFP$_{1-10}$	*MATa met15Δ0; ΔMDM::LEU2; pTDH-KAR2SS-FlucDM-HA-GFP$_{11}$-HDEL-URA3; pTDH-MTS-mCherry-GFP$_{1-10}$-HIS3*	SD Leu$^-$ Ura$^-$ His$^-$	This Study
ΔMDM34<br>yMTS-mCherry-GFP$_{1-10}$	*MATa met15Δ0 ura3Δ0; ΔMDM34::LEU2; pTDH-MTS-mCherry-GFP$_{1-10}$-HIS3*	SD Leu$^-$ His$^-$	This Study
ΔMDM34<br>yMTS-GFP$_{11}$;<br>yMTS-mCherry-GFP$_{1-10}$	*MATa met15Δ0; ΔMDM34::LEU2; pTDH-MTS-GFP$_{11}$-URA3; pTDH-MTS-mCherry-GFP$_{1-10}$-HIS3*	SD Leu$^-$ Ura$^-$ His$^-$	This Study
ΔMDM34<br>yER-FlucDM-GFP$_{11}$;<br>yMTS-mCherry-GFP$_{1-10}$	*MATa met15Δ0; ΔMDM34::LEU2; pTDH-KAR2SS-FlucDM-HA-GFP$_{11}$-HDEL-URA3; pTDH-MTS-mCherry-GFP$_{1-10}$-HIS3*	SD Leu$^-$ Ura$^-$ His$^-$	This Study
ΔMMM1<br>yMTS-mCherry-GFP$_{1-10}$	*MATa met15Δ0 ura3Δ0; ΔMMM1::LEU2; pTDH-MTS-mCherry-GFP$_{1-10}$-HIS3*	SD Leu$^-$ His$^-$	This Study
ΔMMM1<br>yMTS-GFP$_{11}$;<br>yMTS-mCherry-GFP$_{1-10}$	*MATa met15Δ0; ΔMMM1::LEU2; pTDH-MTS-GFP$_{11}$-URA3; pTDH-MTS-mCherry-GFP$_{1-10}$-HIS3*	SD Leu$^-$ Ura$^-$ His$^-$	This Study
ΔMMM1<br>yER-FlucDM-GFP$_{11}$;<br>yMTS-mCherry-GFP$_{1-10}$	*MATa met15Δ0; ΔMMM1::LEU2; pTDH-KAR2SS-FlucDM-HA-GFP$_{11}$-HDEL-URA3; pTDH-MTS-mCherry-GFP$_{1-10}$-HIS3*	SD Leu$^-$ Ura$^-$ His$^-$	This Study
yER-FlucDM-GFP	*MATa his3Δ1 leu2Δ0 met15Δ0; pTDH-KAR2SS-FlucDM-HA-GFP-HDEL-URA3*	SD Ura$^-$	This Study
yElo3-mCherry	*MATa leu2Δ0 met15Δ0 ura3Δ0; Elo3-mCherry-HIS3*	SD His$^-$	This Study
yER-FlucDM-GFP;<br>yElo3-mCherry	*MATa leu2Δ0 met15Δ0; pTDH-KAR2SS-FlucDM-GFP-HDEL-URA3; Elo3-mCherry-HIS3*	SD Ura$^-$ His$^-$	This Study
ΔMDM10<br>yMTS-mCherry-GFP$_{1-10}$;<br>MDM10 WT-Myc	*MATa ura3Δ0; ΔMDM10::LEU2; pTDH-MTS-mCherry-GFP$_{1-10}$-HIS3; pTDH-MDM10WT-Myc-MET15*	SD Leu$^-$ His$^-$ Met$^-$	This Study
ΔMDM10<br>yMTS-GFP$_{11}$;<br>yMTS-mCherry-GFP$_{1-10}$;<br>MDM10 WT-Myc	*MATa; ΔMDM10::LEU2; pTDH-MTS-GFP$_{11}$-URA3; pTDH-MTS-mCherry-GFP$_{1-10}$-HIS3; pTDH-MDM10WT-Myc-MET15*	SD Leu$^-$ Ura$^-$ His$^-$ Met$^-$	This Study
ΔMDM10<br>yER-FlucDM-GFP$_{11}$;<br>yMTS-mCherry-GFP$_{1-10}$;<br>MDM10 WT-Myc	*MATa; ΔMDM10::LEU2; pTDH-KAR2SS-FlucDM-HA-GFP$_{11}$-HDEL-URA3; pTDH-MTS-mCherry-GFP$_{1-10}$-HIS3; pTDH-MDM10WT-Myc-MET15*	SD Leu$^-$ Ura$^-$ His$^-$ Met$^-$	This Study
