# Supplementary material for: ER-misfolded proteins become sequestered with mitochondria and impair mitochondrial function
Source: Commun Biol. 2021 Dec 2;4:1350. doi: 10.1038/s42003-021-02873-w (PMC8640021; doi:10.1038/s42003-021-02873-w)
Supplement: Supplementary file 2 — Description of Additional Supplementary Files [file 42003_2021_2873_MOESM2_ESM.pdf]

Description of Additional Supplementary Files

File name: Supplementary Data

Description: The Supplementary data consists of two Excel files. The first contains the raw data used to draw charts in the main figures. The second file contains row data used to create charts for Supplementary figures.
