# Supplementary material for: ER-misfolded proteins become sequestered with mitochondria and impair mitochondrial function
Source: Commun Biol. 2021 Dec 2;4:1350. doi: 10.1038/s42003-021-02873-w (PMC8640021; doi:10.1038/s42003-021-02873-w)
Supplement: Supplementary file 4 — Reporting Summary [file 42003_2021_2873_MOESM4_ESM.pdf]

nature portfolio

Corresponding author(s): Prof. Dr. Erik C Böttger

Last updated by author(s): 10/18/2021

Reporting Summary

Nature Portfolio wishes to improve the reproducibility of the work that we publish. This form provides structure for consistency and transparency in reporting. For further information on Nature Portfolio policies, see our Editorial Policies and the Editorial Policy Checklist.

Statistics

For all statistical analyses, confirm that the following items are present in the figure legend, table legend, main text, or Methods section.

n/a	Confirmed	
☐	☒	The exact sample size (*n*) for each experimental group/condition, given as a discrete number and unit of measurement
☐	☒	A statement on whether measurements were taken from distinct samples or whether the same sample was measured repeatedly
☐	☒	The statistical test(s) used AND whether they are one- or two-sided<br>*Only common tests should be described solely by name; describe more complex techniques in the Methods section.*
☐	☒	A description of all covariates tested
☐	☒	A description of any assumptions or corrections, such as tests of normality and adjustment for multiple comparisons
☐	☒	A full description of the statistical parameters including central tendency (e.g. means) or other basic estimates (e.g. regression coefficient) AND variation (e.g. standard deviation) or associated estimates of uncertainty (e.g. confidence intervals)
☐	☒	For null hypothesis testing, the test statistic (e.g. *F*, *t*, *r*) with confidence intervals, effect sizes, degrees of freedom and *P* value noted<br>*Give P values as exact values whenever suitable.*
☒	☐	For Bayesian analysis, information on the choice of priors and Markov chain Monte Carlo settings
☒	☐	For hierarchical and complex designs, identification of the appropriate level for tests and full reporting of outcomes
☒	☐	Estimates of effect sizes (e.g. Cohen's *d*, Pearson's *r*), indicating how they were calculated

Our web collection on statistics for biologists contains articles on many of the points above.

Software and code

Policy information about availability of computer code

Data collection	Data was not collected using any code in this study.
Data analysis	There was no original custom code used in this analysis.

For manuscripts utilizing custom algorithms or software that are central to the research but not yet described in published literature, software must be made available to editors and reviewers. We strongly encourage code deposition in a community repository (e.g. GitHub). See the Nature Portfolio guidelines for submitting code & software for further information.

Data

Policy information about availability of data

All manuscripts must include a data availability statement. This statement should provide the following information, where applicable:

- Accession codes, unique identifiers, or web links for publicly available datasets
- A description of any restrictions on data availability
- For clinical datasets or third party data, please ensure that the statement adheres to our policy

Raw data are provided in Supplementary Data consisting of 2 Excel files. The first one contains the row data used for generating the main figures' charts. The second one contains the row data used for generating charts for Supplementary figures.

Field-specific reporting

Please select the one below that is the best fit for your research. If you are not sure, read the appropriate sections before making your selection.

☒ Life sciences ☐ Behavioural & social sciences ☐ Ecological, evolutionary & environmental sciences

For a reference copy of the document with all sections, see nature.com/documents/nr-reporting-summary-flat.pdf

Life sciences study design

All studies must disclose on these points even when the disclosure is negative.

Sample size	The number of samples was sufficient for adequate statistical analysis.
Data exclusions	No data were excluded from analysis.
Replication	Findings were reproducible across the experiments. Experiments were repeated independently at least 3 times.
Randomization	Randomization is not relevant to our study design.
Blinding	Blinding was not necessary in this study.

Reporting for specific materials, systems and methods

We require information from authors about some types of materials, experimental systems and methods used in many studies. Here, indicate whether each material, system or method listed is relevant to your study. If you are not sure if a list item applies to your research, read the appropriate section before selecting a response.

Materials & experimental systems

n/a	Involved in the study	
☐	☒	Antibodies
☐	☒	Eukaryotic cell lines
☒	☐	Palaeontology and archaeology
☒	☐	Animals and other organisms
☒	☐	Human research participants
☒	☐	Clinical data
☒	☐	Dual use research of concern

Methods

n/a	Involved in the study	
☒	☐	ChIP-seq
☐	☒	Flow cytometry
☒	☐	MRI-based neuroimaging

Antibodies

Antibodies used	polyclonal anti-Myc (Abcam ab9106), rabbit polyclonal anti-Firefly luciferase (Abcam ab21176), rabbit polyclonal anti-Tubulin (Abcam ab6046), Total OXPHOS antibody cocktail (Abcam ab110413), anti-GRP94 (Abcam ab3674), anti-GRP78 (Abcam ab21685), anti-TOM70 (Abcam 89624), anti-TIM23 (Abcam 116329), HA-tag antibodies (ab137838, rabbit) and/or anti-TOM20 antibodies (ab56783, mouse), anti-mouse (ab175661, Alexa-405), anti-rabbit (ab97077, Cy5).
Validation	All antibodies used have been validated by the supplier for use in Western blotting and/or fluorescent microscopy.

Eukaryotic cell lines

Policy information about cell lines

Cell line source(s)	ATCC cell collection library.
Authentication	Cell lines were authenticated by supplier.
Mycoplasma contamination	Cell lines were not contaminated with Mycoplasma.
Commonly misidentified lines (See ICLAC register)	No

Flow Cytometry

Plots

Confirm that:

☒ The axis labels state the marker and fluorochrome used (e.g. CD4-FITC).

☒ The axis scales are clearly visible. Include numbers along axes only for bottom left plot of group (a 'group' is an analysis of identical markers).

☒ All plots are contour plots with outliers or pseudocolor plots.

☒ A numerical value for number of cells or percentage (with statistics) is provided.

Methodology

Sample preparation	Sample preparation were described in the manuscript's material and methods part.
Instrument	The instruments are identified in the manuscript.
Software	FlowJo
Cell population abundance	Cells were not sorted and abundance was never the limiting factor.
Gating strategy	We applied forward and side scatter parameters (FSC, SSC) to exclude cell debris and doublets. Typically, up to 30% of events were excluded to remove events that could not be assigned as alive single cells. No additional gating was applied.

☒ Tick this box to confirm that a figure exemplifying the gating strategy is provided in the Supplementary Information.
